# Rational Design of *N*‐2‐(7‐Chloroquinolin‐4‐ylamino)ethyl Amides Active Against Chloroquine‐Resistant *Plasmodium falciparum*

**DOI:** 10.1002/ardp.70350

**Published:** 2026-10-04

**Authors:** Everton P. Silva, Camila S. Barbosa, Anna C. C. Aguiar, Ramon G. Oliveira, Thiago A. M. Brito, Marcelo F. R. Silva, Helivaldo D. S. Souza, Petrônio F. Athayde‐Filho, Gabriela F. Fiss

**Affiliations:** ^1^ Laboratório de Pesquisa em Bioenergia e Síntese Orgânica (LPBS), Department of Chemistry Universidade Federal da Paraíba (UFPB) João Pessoa Brazil; ^2^ Department of Microbiology, Immunology and Parasitology Universidade Federal de São Paulo (UNIFESP) São Paulo Brazil; ^3^ Institute of Chemistry Universidade Estadual de Campinas (UNICAMP) Campinas Brazil; ^4^ Laboratório Multiusuário de Caracterização e Análises (LMCA), Instituto de Pesquisa em Fármacos e Medicamentos (IPeFarM), UFPB João Pessoa Brazil

**Keywords:** antiplasmodial activity, cytotoxicity, resistance index, selectivity index, structure–activity relationship (SAR)

## Abstract

Motivated by the antimalarial potential demonstrated by benzamide derivatives of 4‐alkylamino‐7‐chloroquinolines against chloroquine‐resistant *Plasmodium falciparum*, we designed and synthesized a rational series of *N*‐2‐(7‐chloroquinolin‐4‐ylamino)ethyl amides to investigate the effects of electron‐withdrawing and donating substituents on antiplasmodial and cytotoxicity properties, from prediction to in vitro assays. From an initial screening of 22 chloroquine (CQ) analogs, 16 compounds with two distal amino groups in the alkyl linker showed IC_50_ values below 1000 nM against the CQ‐sensitive 3D7 strain. Next, their activity against the CQ‐resistant K1 strain, in silico toxicity, predicted using the pkCSM program, and in vitro cytotoxicity on HepG2 cells were evaluated. Benzamides containing electron‐withdrawing chlorine and nitro groups showed the most promising profiles, with IC_50_ values against the CQ‐resistant K1 strain (IC_50_^K1^, 55–72 nM) and resistance index (RI, 0.7–1.9) values outperforming CQ (IC_50_^K1^ = 141 nM, RI = 7.1). Although predicted to be hepatotoxic, these compounds were not cytotoxic to HepG2 cells at the tested concentrations, showing CC_50_ values above 12,500 nM and selectivity indices greater than 115. Compared with CQ, a 3D qualitative SAR model indicated that increased lipophilicity around the distal benzamide moiety enhances antiplasmodial activity against the K1 strain, contributing to the observed potency.

## Graphical abstract

Of 22 chloroquine (CQ) analogs, 16 showed IC_50_^3D7^ values of 27–555 nM; their activity against the CQ‐resistant K1 strain and in silico/vitro hepatotoxicity were evaluated. Benzamides with chlorine and nitro groups were most active, showing IC_50_^K1^ and RI values lower than CQ and cytotoxic concentrations well above inhibitory concentrations.

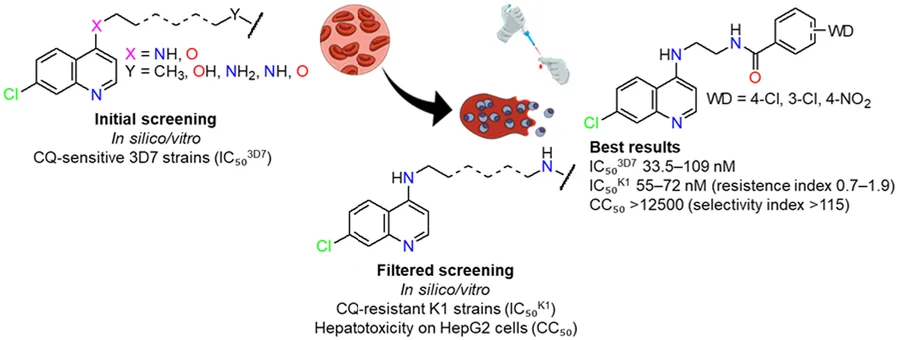

## 1 Introduction

Six different species of *Plasmodium* can be transmitted from an infected female *Anopheles* mosquito to humans: *P. knowlesi*, *P. malariae*, *P. ovale curtisi*, *P. ovale wallikeri* and *P. vivax* [1, 2]. According to the World Malaria Report 2025 [3], the estimated number of clinical malaria cases and deaths is 282 million cases and 610 thousand deaths in 2024—approximately 9 million more cases than the previous year. The African region continues to bear the greatest burden, with 11 countries accounting for about two‐thirds of cases and deaths worldwide. Despite progress in reducing the malaria mortality rate, we are still far from achieving the goals of the Global Transition Strategy (GTS), established in 2000. In an attempt to control malaria, including the use of quinine, a natural alkaloid extracted from the bark of trees of the genus *Cinchona*, native to the Andes Mountains, extensive research on antimalarial drugs in the 1930s resulted in quinacrine **I** and chloroquine (CQ) **II** (Figure 1). For over 40 years, the antimalarial properties of CQ have been well tolerated by most people, compared with other synthetic quinoline derivatives. Unfortunately, CQ‐resistant parasites have spread rapidly in recent decades, reducing its effectiveness, and other 7‐chloroquinolines, such as hydroxychloroquine **III**, amodiaquine **IV**, and piperaquine **V** (Figure 1), including in combination therapies, have been used in the prophylaxis and treatment of malaria [4, 5, 6, 7].

**Figure 1:**
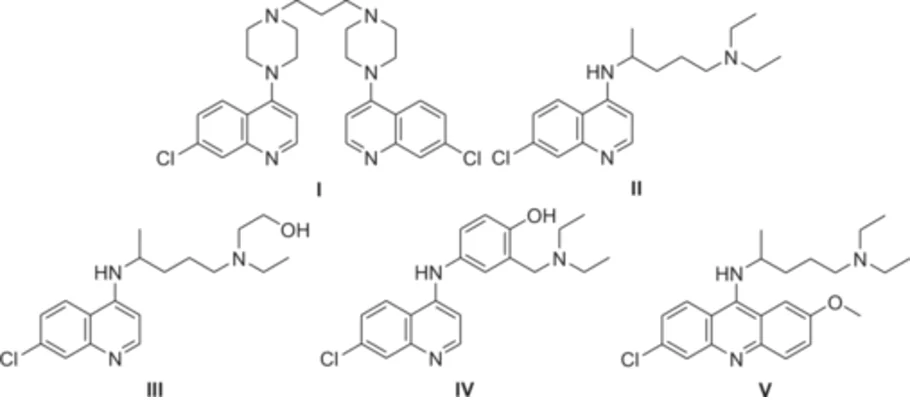
7‐Chloroquinolines as antimalarial drugs: chloroquine **I**, hydroxychloroquine **II**, amodiaquine I**II**, piperaquine I**V**, quinacrine **V**.

Artemisinin‐based combination therapies are the recommended first‐line treatments for malaria caused by *P. plasmodium*, the most common and lethal form. However, more than 17 years ago, the first signs of *P. falciparum* resistance to these combination therapies were observed along the Thai border [8], highlighting the urgency need to search for alternative antimalarial agents. In this regard, benzamide derivatives of 4‐alkylamino‐7‐chloroquinolines have demonstrated promising activity against CQ‐resistant *P. falciparum* [9, 10]. Among them, a series of 3‐substituted CQ derivatives was evaluated against CQ‐sensitive (3D7) and CQ‐resistant (Dd2) strains of *P. falciparum*. The aroylation of 3‐aminochloroquines revealed that *para*‐substituted 3‐benzamidochloroquines **VI** (Figure 2) resulted in the lowest 50% inhibitory concentration (IC_50_) values against the 3D7 strain, and 3‐*para*‐chlorobenzamidochloroquine was selected for potential use in combination therapy with CQ, resulting in a lower IC_50_ [9]. In another work, the antiplasmodial activity against 3D7 and Dd2 strains was evaluated for 4‐butylamino‐7‐chloroquinoline derivatives. In the same study, benzamide derivatives of 4‐butylamino‐7‐chloroquinolines **VII** (Figure 2) showed significant activity against the 3D7 strain [10]. Alternatively, 2‐(7‐chloroquinolin‐4‐ylamino)ethyl benzoates **VIII** (Figure 2) were evaluated in vivo against the CQ‐sensitive (ANKA) *P. berghei* strain, a parasite that causes malaria in rodents [11].

**Figure 2:**
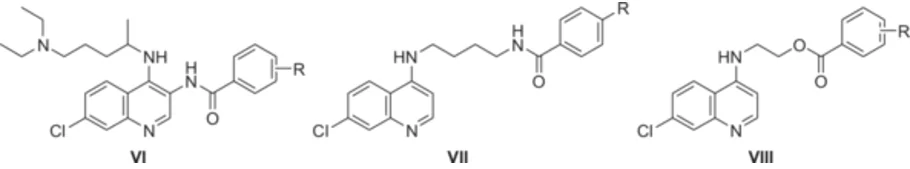
Promising 4‐alkylamino‐7‐chloroquinolines as antiplasmodial agents: 3‐benzamidochloroquines **VI**, benzamide derivatives of 4‐butylamino‐7‐chloroquinolines **VII**, 2‐(7‐chloroquinolin‐4‐ylamino)ethyl benzoates **VIII**.

Our research group has been dedicated to covering quinoline derivatives at the molecular and supramolecular levels [12], searching for cleaner approaches, as well as bioactive quinolines that are less toxic to the environment. Some results confirm the potential bioactivities of quinolines, such as antileishmanial [13, 14], antimicrobial [15], and larvicidal activity against *Aedes aegypti* [16]. Given that it is well known that alkyldiamino and chlorine groups at the C4 and C7 positions of quinoline, respectively, are essential for antiplasmodial activity [17, 18], as well as the antimalarial potential demonstrated by benzamide derivatives of 4‐alkylamino‐7‐chloroquinolines against CQ‐resistant *P. falciparum*, we rationally designed a series of *N*‐2‐(7‐chloroquinolin‐4‐ylamino)ethyl amides to investigate how electron‐withdrawing and donating substituents affect antiplasmodial and cytotoxicity properties, from prediction to in vitro assays.

## 2 Results and Discussion

### 2.1 Chemistry

The synthetic design for obtaining of *N*‐2‐(7‐chloroquinolin‐4‐ylamino)ethyl amides and their derivatives is shown in Scheme 1. 4‐Alkoxy/alkylamino‐7‐chloroquinolines (**2–9**) were prepared from the nucleophilic aromatic substitution (S_N_Ar) reaction of 4,7‐dichloroquinoline with an appropriate alcohol/amine in yields ranging from 81% to 100% [15]. Among them, considering the most promising antiplasmodial profile for precursor **6**, this was selected to react with different acyl chlorides, while compounds **11–12** were synthesized for bioactivity comparison purposes. Thus, after the necessary conditions and reaction times (see Scheme 1), *N*‐2‐(7‐chloroquinolin‐4‐ylamino)ethyl amides **10a–l** and 2‐(7‐chloroquinolin‐4‐ylamino/oxy)ethyl benzoates **11–12** were obtained after filtration with distilled water, without the need for an additional purification step such as liquid‐liquid extraction and/or column chromatography.

**Scheme 1:**
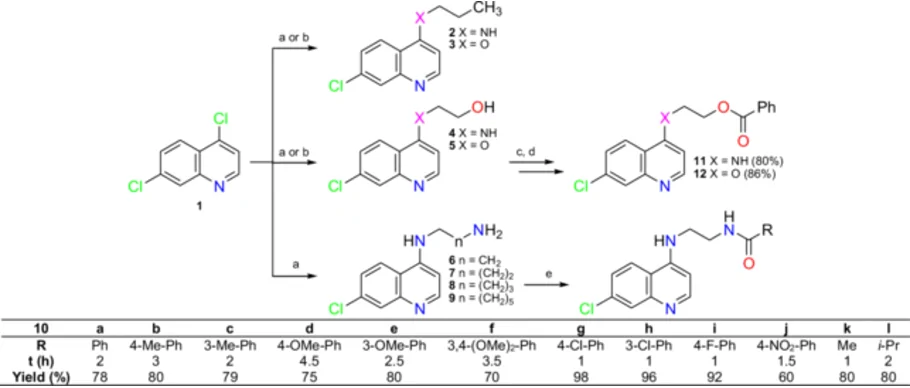
Reagents and reactional conditions to obtain *N*‐2‐(7‐chloroquinolin‐4‐ylamino)ethyl amides and their derivatives. X = NH, a: amine, 80°C, 4 h (for propylamine, 50°C for 12 h); X = O, b: alcohol, *t*‐BuOK, 100°C, 8 h; c: SOCl_2_, DCM, 25°C, 6 h; d: potassium benzoate, DMF, 100°C, 2 h; n = CH_2_, e: acyl chloride, TEA, DMF, 0°C–25°C.

The newly synthesized compounds (**10b–f**, **10h–i**, **10k–l**, **11–12**) were characterized by spectroscopic techniques of infrared (IR), and ^1^H and ^13^C nuclear magnetic resonance (NMR). Furthermore, high‐resolution mass spectrometry (HRMS) analyses confirmed the exact masses of compounds with errors of less than 5 ppm. Regarding the ^1^H NMR spectra of the compounds, the main signal that confirms the formation of products is the decrease/absence of the signal corresponding to the NH_2_/OH group belonging to precursors. According to the ^13^C NMR spectra, the main signal confirming the formation of products is the signal corresponding to the carbonyl carbon at 165.3–176.9 ppm. Finally, in the IR spectra, the most significant changes compared with their respective precursors were the appearance of stretching related to C═O at 1624–1716 cm^–1^, an increase in symmetric/asymmetric stretching and in‐plane/out‐of‐plane angular deformations related to C–N/O at 1300–1022 cm^–1^, and a decrease/absence of a broad band related to H–N/O at 3363–3209 cm^–1^.

### 2.2 Pharmacology

#### 2.2.1 In Silico/Vitro Antiplasmodial Evaluation

For 4‐alkoxy/alkylamino‐7‐chloroquinolines (**2–9**), *N*‐2‐(7‐chloroquinolin‐4‐ylamino)ethyl amides (**10a–l**), 2‐(7‐chloroquinolin‐4‐ylamino/oxy)ethyl benzoates (**11–12**) and CQ, antiplasmodial activity against 3D7 and K1 strains was evaluated from in silico data, using the AmIActive program [19], and *in vitro* assays. *In silico/vitro* results are available in Table 1.

**Table 1:** *In silico/vitro* antiplasmodial data of 4‐alkoxy/alkylamino‐7‐chloroquinolines (**2–9**), *N*‐2‐(7‐chloroquinolin‐4‐ylamino)ethyl amides (**10a–l**), 2‐(7‐chloroquinolin‐4‐ylamino/oxy)ethyl benzoates (**11–12**) and CQ.

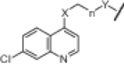					3D7 (CQ‐sensitive)		K1 (CQ‐resistant)		RI (IC_50_^K1^/IC_50_^3D7^)
Compd.	X	n	Y	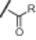	In silico	In vitro	In silico	In vitro	
					Probability active (%)	IC_50_ ± SD (nM)	Probability active (%)	IC_50_ ± SD (nM)	
**2**	NH	CH_2_	CH_3_	—	0.36^a^	> 1000	0.42^a^	—	—
**3**	O	CH_2_	CH_3_	—	0.41^a^	> 1000	0.41^a^	—	—
**4**	NH	CH_2_	OH	—	0.32^a^	> 1000	0.40^a^	—	—
**5**	O	CH_2_	OH	—	0.33^a^	> 1000	0.43^a^	—	—
**6**	NH	CH_2_	NH_2_	—	0.39^a^	29 ± 11	0.45^a^	95 ± 9	3.3
**7**	NH	(CH_2_)_2_	NH_2_	—	0.36^a^	27 ± 9	0.43^a^	206 ± 86	7.6
**8**	NH	(CH_2_)_3_	NH_2_	—	0.33^a^	72 ± 4	0.44^a^	137 ± 46	1.9
**9**	NH	(CH_2_)_5_	NH_2_	—	0.33^a^	206 ± 51	0.44^a^	119 ± 41	0.6
**10a**	NH	CH_2_	NH	Ph	0.35^a^	344 ± 143	0.31^a^	176 ± 50	0.5
**10b**	NH	CH_2_	NH	4‐Me‐Ph	0.35^a^	207 ± 98	0.31^a^	237 ± 126	1.1
**10c**	NH	CH_2_	NH	3‐Me‐Ph	0.41^b^	152 ± 66	0.34^b^	78 ± 21	0.5
**10d**	NH	CH_2_	NH	4‐OMe‐Ph	0.36^b^	208 ± 74	0.31^a^	96 ± 26	0.5
**10e**	NH	CH_2_	NH	3‐OMe‐Ph	0.39^b^	219 ± 76	0.33^b^	108 ± 11	0.5
**10f**	NH	CH_2_	NH	3,4‐(OMe)_2_‐Ph	0.31^b^	210 ± 35	0.29^b^	160.5 ± 0.7	0.8
**10g**	NH	CH_2_	NH	4‐Cl‐Ph	0.32^a^	33.5 ± 0.7	0.34^a^	62 ± 15	1.9
**10h**	NH	CH_2_	NH	3‐Cl‐Ph	0.37^a^	109 ± 47	0.34^a^	72 ± 23	0.7
**10i**	NH	CH_2_	NH	4‐F‐Ph	0.37^a^	258 ± 95	0.32^a^	150 ± 50	0.6
**10j**	NH	CH_2_	NH	4‐NO_2_‐Ph	0.32^b^	53 ± 22	0.28^b^	55 ± 17	1.0
**10k**	NH	CH_2_	NH	*i*‐Pr	0.32^a^	247 ± 90	0.31^a^	143 ± 23	0.6
**10l**	NH	CH_2_	NH	Me	0.35^a^	555 ± 169	0.31^a^	183 ± 6	0.3
**11**	NH	CH_2_	O	Ph	0.38^a^	> 1000	0.34^a^	—	—
**12**	O	CH_2_	O	Ph	0.43^a^	> 1000	0.39^a^	—	—
Chloroquine (CQ)					0.48^a^	20 ± 6	0.51^a^	141 ± 54	7.1

Abbreviations: RI, resistance index; SD, standard deviation.

^a^ Reliable.

^b^ Unreliable. IC_50_: 50% inhibitory concentration.

The prediction of antiplasmodial activity was performed for comparison with experimental data. AmIActive is a large‐scale QSAR based on target fishing and polypharmacology, which provides information on the predictive bioactivity of molecules, as well as their probability. If a molecule is considered outside the applicability domain of a given model, the model's prediction for that molecule should not be considered reliable, regardless of the predicted outcome or its probability. According to Table 1, compounds **10c–f** and **10j** are outside the applicability domain. Although the predictions for some compounds, including CQ, are considered reliable, they indicate a low probability of activity; nevertheless, these data may be used to expand and improve the model database.

Considering the IC_50_ value of CQ in the nanomolar range, an IC_50_ cutoff value of 1000 nM was established against the CQ‐sensitive (3D7) strain. Thus, 16 compounds showed IC_50_^3D7^ values below 1000 nM, ranging from 27 to 555 nM, and were also evaluated against the CQ‐resistant (K1) strain. The impact of the introduced variations was rationalized through a structure–activity relationship (SAR) analysis. According to Table 1, the presence of methyl (compounds **2–3**), hydroxyl (compounds **4–5**), or benzoyl (compounds **11–12**) groups in the terminal side chain did not favor antiplasmodial activity (IC_50_^3D7^ > 1000 nM). On the other hand, compounds **6–9**, which have an amino group in the terminal side chain, showed significant antiplasmodial activity (IC_50_^3D7^ 27–206 nM). Egan et al. had already established that, for 7‐chloroquinolines, an alkyldiamino group at the C4 position of the quinoline is essential for antiplasmodial activity [17, 18]. Regarding side chain length, a chain of 2 (compound **6**) to 3 (compound **7**) carbons increased antiplasmodial activity. In a previous systematic study [20], side chain lengths ≤ 3 or ≥ 10 carbons were identified as determinants of activity against a CQ‐resistant *P. falciparum* strain through SAR and two‐dimensional structures analyses. Furthermore, benzamide derivatives of 4‐butylamino‐7‐chloroquinolines (see **VII**, Figure 2), derivatives of the **10** series, showed higher IC_50_^3D7^ values (650–720 nM) for a chain of 4 carbons [10].

Considering that compound **6** presented a lower IC_50_^K1^ value than compound **7**, as well as a greater resistance index (RI), it was selected to react with different acyl chlorides, leading to the formation of *N*‐2‐(7‐chloroquinolin‐4‐ylamino)ethyl amides (**10a–l**) to investigate the antiplasmodial effect of electron‐withdrawing and donating substituents. The electron‐withdrawing chlorine and nitro groups (compounds **10g**, **10h**, and **10j**) resulted in the lowest IC_50_^3D7^ (33.5–109 nM) and IC_50_^K1^ (55–72 nM) values, especially for the *para*‐substituted compounds (**10g** and **10j**), which corroborates what has been recently described in the literature [9] (see **VI**, Figure 2). Finally, compounds **10k–l** did not show improvement in antiplasmodial activity when aryl groups were replaced by alkyl groups.

Regarding the RI parameter, ratios lower than fivefold between CQ‐sensitive and CQ‐resistant strains are more promising for advancing the development pipeline [21]. All compounds evaluated against the K1 strain showed inhibitory effects comparable to those of the 3D7 strain (IC_50_^K1^ values ranging from 55 to 237 nM), with the exception of compound **7**, which was sevenfold less potent against the resistant strain (RI = 7.6), indicating cross‐resistance to CQ (RI = 7.1). The remaining compounds exhibited RI values ranging from 0.3 to 3.3, including the most active compounds **10g**, **10h**, and **10j** (RI: 0.7–1.9).

#### 2.2.2 In Silico/Vitro Toxicity Evaluation

Since 4‐alkoxy/alkylamino‐7‐chloroquinolines (**2–6**) and *N*‐2‐(7‐chloroquinolin‐4‐ylamino)ethyl amides (**10a–l**) showed significant antiplasmodial activity (IC_50_^3D7^ 27–555 nM), their predictive toxicity was calculated using the pkCSM program [22], and in vitro cytotoxicity was evaluated in the human hepatocellular carcinoma cell line HepG2. In silico/vitro data are available in Table 2.

**Table 2:** *In silico/vitro* toxicity data of 4‐alkoxy/alkylamino‐7‐chloroquinolines (**6–9**) and *N*‐2‐(7‐chloroquinolin‐4‐ylamino)ethyl amides (**10a–l**).

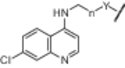				In silico toxicity			In vitro		
Compd.	n	Y	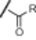	AMES^a^	Hepato	Skin sensitisation	IC_50_^3D7^±SD (nM)	CC_50_ HepG2 (nM)	SI (CC_50_^HepG2^/IC_50_^3D7^)
**6**	CH_2_	NH_2_	—	No	No	No	29 ± 11	> 50000	> 1744
**7**	(CH_2_)_2_	NH_2_	—	No	No	No	27 ± 9	> 50000	> 1852
**8**	(CH_2_)_3_	NH_2_	—	No	Yes	No	72 ± 4	> 25000	> 347
**9**	(CH_2_)_5_	NH_2_	—	No	Yes	No	206 ± 51	> 50000	> 243
**10a**	CH_2_	NH	Ph	No	Yes	No	344 ± 143	> 50000	> 145
**10b**	CH_2_	NH	4‐Me‐Ph	No	Yes	No	207 ± 98	> 12500	> 60
**10c**	CH_2_	NH	3‐Me‐Ph	No	Yes	No	152 ± 66	> 12500	> 82
**10d**	CH_2_	NH	4‐OMe‐Ph	No	Yes	No	208 ± 74	> 25000	> 120
**10e**	CH_2_	NH	3‐OMe‐Ph	No	Yes	No	219 ± 76	> 50000	> 228
**10f**	CH_2_	NH	3,4‐(OMe)_2_‐Ph	No	Yes	No	210 ± 35	> 50000	> 238
**10g**	CH_2_	NH	4‐Cl‐Ph	No	Yes	No	33.5 ± 0.7	> 12500	> 373
**10h**	CH_2_	NH	3‐Cl‐Ph	No	Yes	No	109 ± 47	> 12500	> 115
**10i**	CH_2_	NH	4‐F‐Ph	No	Yes	No	258 ± 95	> 50000	> 194
**10j**	CH_2_	NH	4‐NO_2_‐Ph	Yes	Yes	No	53 ± 22	> 12500	> 236
**10k**	CH_2_	NH	*i*‐Pr	Yes	No	No	247 ± 90	> 100000	> 405
**10l**	CH_2_	NH	Me	No	No	No	555 ± 169	> 100000	> 180

Abbreviations: CC_50_, 50% cytotoxicity concentration; IC_50_, 50% inhibitory concentration; SD, standard deviation; SI, selectivity index.

^a^ Mutagenic potential using bacteria.

The pkCSM program predicts the pharmacokinetic properties of small molecules using graph‐based signatures. The AMES test is a predictive method used to assess the mutagenic potential of a compound using bacteria. This predictive model was developed based on the results of over 8000 compounds. A compound is classified as potentially hepatotoxic if it has the potential to cause at least one pathological or physiological event strongly associated with liver dysfunction. The hepatotoxicity prediction model was developed using data on hepatic side effects observed in humans for 531 compounds. Skin sensitisation predicts whether a compound may induce allergic contact dermatitis. This predictor was developed using data from 254 compounds associated with skin sensitisation. According to Table 2, with the exception of compounds **10j–k**, the remaining compounds have no mutagenic potential. On the other hand, except for compounds **6–7** and **10k–l**, the remaining compounds are potentially hepatotoxic. Furthermore, none of the compounds has the potential to cause skin sensitization.

Although some compounds are predicted to be hepatotoxic, none was cytotoxic to HepG2 cells at the tested concentrations, exhibiting a selectivity index (SI) greater than 60‐fold. Consistently, compounds predicted to be non‐hepatotoxic were the least cytotoxic to HepG2 cells, reaching 50% cytotoxicity concentration (CC_50_) values above 10,000 nM. With regard to the compounds (**10 g**, **10h**, and **10j**) with the most promising antiplasmodial activity, they also showed a favorable hepatotoxicity profile, with cytotoxic concentrations well above the inhibitory concentrations.

### 2.3 3D Qualitative SAR Model

For a better understanding of the preliminary SAR analysis of the most promising compounds (**10g**, **10h**, and **10j**) compared with CQ, a qualitative SAR model was built using the Activity Atlas module available in Forge v10.6.0 [23]. The ligand‐based pharmacophore hypothesis (Figure 3A) was used to align a dataset of *N*‐2‐(7‐chloroquinolin‐4‐ylamino)ethyl amides (Figure 3B). Regarding the most active molecules, the proposed model highlights regions considered critical for the observed potency and SAR cliffs, indicating that increased lipophilicity (green areas) around the distal benzamide moiety favors antiplasmodial activity. The importance of two distal amino groups in the alkyl linker is evidenced by the positive electrostatic regions (red areas), which contribute to potency (Figure 2C).

**Figure 3:**
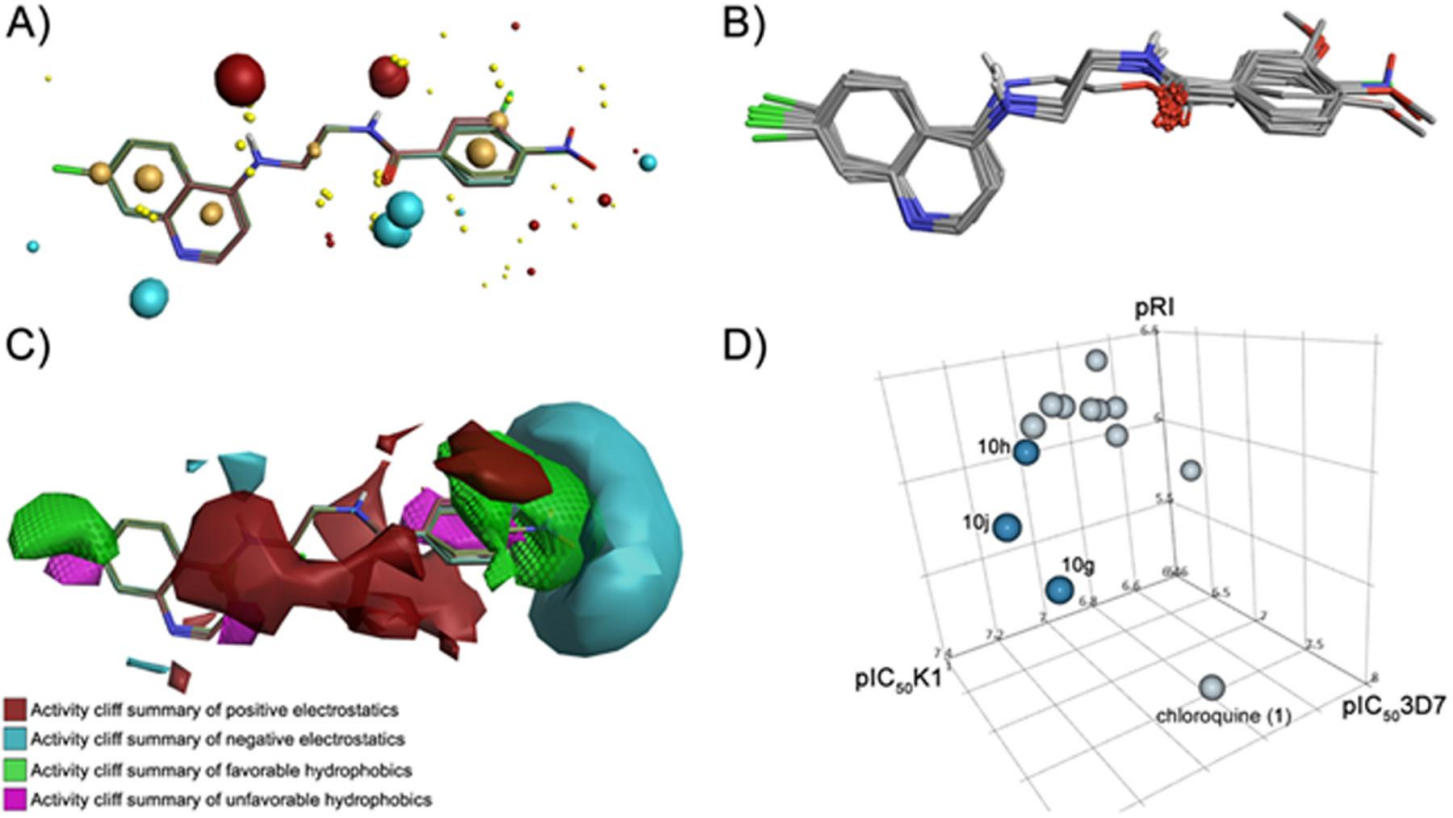
(A) Ligand‐based pharmacophore hypothesis, (B) Alignment of *N*‐2‐(7‐chloroquinolin‐4‐ylamino)ethyl amides, (C) Activity Atlas, (D) Scatter plot of potency as a function of inhibitory concentrations (–log IC_50_) and resistance indices (–log RI).

As discussed earlier, one of the characteristics of these compounds (**10g**, **10h**, and **10j**), compared with CQ, is their ability to maintain antiplasmodial activity even in the CQ‐resistant (K1) strain, as indicated by the IC_50_^K1^ and RI values (Table 1). A scatter plot of pRI and pIC_50_ (3D7 and K1) values shows that compounds **10g**, **10h**, and **10j** stand out from their derivatives and CQ (Figure 2D), exhibiting greater pRI and pIC_50_ values.

## 3 Conclusion

Concerned about the high capacity of *P. falciparum* to develop resistance to antimalarial drugs, we investigated the influence of 22 CQ analogs on their antiplasmodial and cytotoxicity properties. To compose the series, 12 *N*‐2‐(7‐chloroquinolin‐4‐ylamino)ethyl amides were obtained through a cleaner approach, with yields of 60%–98%. After a filtered screening, 16 compounds with two distal amino groups in the alkyl linker showed submicromolar activity against the CQ‐sensitive 3D7 strain, and their activity against the CQ‐resistant K1 strain as well as their and in silico/vitro toxicity were evaluated. The best results were obtained for benzamides containing electron‐withdrawing chlorine and nitro groups (**10g**, **10h**, and **10j**), which showed lower resistance indices than CQ. Despite the predicted hepatotoxic potential, these compounds were not cytotoxic to HepG2 cells at the tested concentrations, exhibiting cytotoxic concentrations well above their inhibitory concentrations. A 3D qualitative SAR model corroborated our findings, indicating that increased lipophilicity around the distal benzamide moiety enhances antiplasmodial activity against the K1 strain, thereby overcoming CQ‐resistant *P. falciparum*. Further investigations into safety profiles, along with a more detailed SAR analysis, may help guide future *in vivo* studies and clinical trials of these promising antiplasmodial candidates.

## 4 Experimental

### 4.1 Chemistry

#### 4.1.1 General

All common reagents and solvents were purchased from commercial suppliers and used without further purification. Compounds were synthesized at the Laboratório de Pesquisa em Bioenergia e Síntese Orgânica (LPBS‐UFPB), and melting points were measured in triplicate using a Microquímica MQAPF‐301 instrument (Palhoça, Brazil), and a Fisatom 431D instrument (São Paulo, Brazil). ^1^H and ^13^C NMR spectra were acquired at the Laboratório Multiusuário de Caracterização e Análises (LMCA‐UFPB) using Bruker Ascend (Coventry, UK) and Bruker (Billerica, MA, USA) spectrometers at 400 and 500 MHz for ^1^H, respectively, using 5 mm tubes at 298 K. IR spectra were obtained at the Laboratório de Síntese Orgânica Medicinal (LASOM‐UFPB) using a Shimadzu IRPrestige‐21 spectrometer (Kyoto, Japan) with KBr pellets. HRMS analyses were performed at LMCA‐UFPB using a Shimadzu HPLC (Kyoto, Japan) coupled to a Bruker MicrOTOF II spectrometer (Billerica, MA, USA) (capillary 4500 V, vaporizer temperature 200°C), and an Orbitrap Exploris 120 Mass Spectrometer (Waltham, MA, USA) (capillary 3500 V, vaporizer temperature 350°C), both with an electrospray ionization (ESI) source and positive mode.

#### 4.1.2 Experimental Procedures for Synthesis of 4‐Alkoxy/Alkylamino‐7‐Chloroquinolines (2–9)

According to methods previously described by Fiss et al. [15], compounds **2–9** were obtained in yields ranging from 81% to 100%, whose data were previously described by our research group.

#### 4.1.3 General Procedure for Synthesis of *N*‐2‐(7‐chloroquinolin‐4‐ylamino)ethyl Amides (10a–l)

Acyl chloride (1.2 mmol) was added dropwise to a stirring mixture of *N*‐(2‐aminoethyl)‐7‐chloroquinolin‐4‐amine (**6**, 1 mmol) and triethylamine (TEA, 1.2 mmol) in anhydrous dimethylformamide (DMF, 1 mL) at 0°C. Next, the reactional mixture was maintained under magnetic stirring at 25°C for 1–4.5 h, which was monitored by thin‐layer chromatography using methanol in ethyl acetate (MeOH in AcOEt) as eluent. Afterward, the mixture was added to a beaker containing a saturated sodium bicarbonate solution (10 mL) and left in a freezer until freezing point. Then, the precipitate was filtered with excess ice water under reduced pressure. Compounds **10a–l** were obtained in yields of 60%–98%. Full NMR, IR, and HRMS spectra for newly synthesized compounds (**10b–f**, **10h–i**, **10k–l**) are available in Supporting Information. All compounds are > 95% pure by HPLC analysis.

*N*‐[2‐(7‐Chloroquinolin‐4‐ylamino)ethyl]benzamide (**10a**) in 78% yield; TLC: (20% MeOH in AcOEt, *R*_f_): 0.5 (UV, I_2_); white solid; mp 307°C–309°C (lit [24]. mp 300°C); ^1^H NMR (DMSO‐*d*_6_, 400 MHz): *δ* 8.63 (t, *J* = 5.5 Hz, 1H, NH), 8.42 (d, *J* = 5.4 Hz, 1H, H_Ar_), 8.20 (d, *J* = 9.0 Hz, 1H, H_Ar_), 7.86–7.84 (m, 2H, H_Ar_), 7.79 (d, *J* = 2.2 Hz, 1H, H_Ar_), 7.53–7.51 (m, 1H, H_Ar_), 7.48–7.44 (m, 3H, H_Ar_), 7.40 (t, *J* = 5.3 Hz, 1H, NH), 6.63 (d, *J* = 5.4 Hz, 1H, H_Ar_), 3.57 (q, *J* = 6.1 Hz, 2H, CH_2_), 3.49 (q, *J* = 5.9 Hz, 2H, CH_2_) ppm; ^13^C NMR (DMSO‐*d*_6_, 101 MHz): δ 166.7 (C═O), 151.7 (C_Ar_), 149.9 (C_Ar_), 148.9 (C_Ar_), 134.2 (C_Ar_), 133.2 (C_Ar_), 131.0 (C_Ar_), 128.1 (C_Ar_), 127.4 (C_Ar_), 127.0 (C_Ar_), 123.9 (C_Ar_), 123.6 (C_Ar_), 117.3 (C_Ar_), 98.5 (C_Ar_), 42.0 (CH_2_), 37.8 (CH_2_) ppm; HRMS (ESI‐TOF) *m/z*: [M + H]^+^ calcd for C_18_H_17_ClN_3_O: 326.1055; found 326.1046.

*N*‐[2‐(7‐Chloroquinolin‐4‐ylamino)ethyl]‐4‐methylbenzamide (**10b**) in 80% yield; TLC: (20% MeOH in AcOEt, *R*_f_): 0.53 (UV, I_2_); white solid; mp 293°C–295°C; ^1^H NMR (DMSO‐*d*_6_, 400 MHz): δ 8.54 (t, *J* = 5.7 Hz, 1H, NH), 8.41 (d, *J* = 5.4 Hz, 1H, H_Ar_), 8.18 (d, *J* = 9.0 Hz, 1H, H_Ar_), 7.79 (d, *J* = 2.2 Hz, 1H, H_Ar_), 7.75 (d, *J* = 8.3 Hz, 2H, H_Ar_), 7.44 (dd, *J* = 9.0, 2.3 Hz, 1H, H_Ar_), 7.38 (t, *J* = 5.6 Hz, 1H, NH), 7.26 (d, *J* = 8.2 Hz, 2H, H_Ar_), 6.62 (d, *J* = 5.5 Hz, 1H, H_Ar_), 3.57 (q, *J* = 6.1 Hz, 2H, CH_2_), 3.50–3.46 (m, 2H, CH_2_), 2.34 (s, 3H, CH_3_) ppm; ^13^C NMR (DMSO‐*d*_6_, 101 MHz): *δ* 166.9 (C═O), 151.7 (C_Ar_), 150.1 (C_Ar_), 148.9 (C_Ar_), 141.0 (C_Ar_), 133.4 (C_Ar_), 131.5 (C_Ar_), 128.7 (C_Ar_), 127.3 (C_Ar_), 127.0 (C_Ar_), 124.0 (C_Ar_), 123.7 (C_Ar_), 117.3 (C_Ar_), 98.6 (C_Ar_), 42.2 (CH_2_), 37.9 (CH_2_), 20.8 (CH_3_) ppm; IR (KBr): ν 3309, 3209 (N–H), 3059, 3035 (H_Ar_), 2974, 2935 (H_alkanic_), 1647 (C═O), 1612 (C═N), 1581, 1539 (C═C), 1296, 1222, 1165, 1138, 1080 (C–N), 852, 794, 740 (Ar) cm^−1^; HRMS (ESI‐TOF) *m/z*: [M + H]^+^ calcd for C_19_H_19_ClN_3_O: 340.1211; found 340.1222.

*N*‐[2‐(7‐Chloroquinolin‐4‐ylamino)ethyl]‐3‐methylbenzamide (**10c**) in 79% yield; TLC: (20% MeOH in AcOEt, *R*_f_): 0.53 (UV, I_2_); white solid; mp 250°C–253°C; ^1^H NMR (DMSO‐*d*H NMR (DMSO‐ 400 MHz): *δ* 8.57 (t, *J* = 5.5 Hz, 1H, NH), 8.42 (d, *J* = 5.4 Hz, 1H, H_Ar_), 8.20 (d, *J* = 9.0 Hz, 1H, H_Ar_), 7.79 (d, *J* = 2.2 Hz, 1H, H_Ar_), 7.66 (s, 1H, H_Ar_), 7.65–7.63 (m, 1H, H_Ar_), 7.44 (dd, *J* = 9.0, 2.3 Hz, 1H, H_Ar_), 7.38 (t, *J* = 6.0 Hz, 1H, NH), 7.34–7.33 (m, 2H, H_Ar_), 6.62 (d, *J* = 5.4 Hz, 1H, H_Ar_), 3.57 (q, *J* = 6.1 Hz, 2H, CH_2_), 3.50–3.46 (m, 2H, CH_2_), 2.35 (s, 3H, CH_3_) ppm; ^13^C NMR (DMSO‐*d*_6_, 101 MHz): *δ* 167.0 (C═O), 151.7 (C_Ar_), 150.0 (C_Ar_), 148.9 (C_Ar_), 137.4 (C_Ar_), 134.3 (C_Ar_), 133.3 (C_Ar_), 131.5 (C_Ar_), 128.0 (C_Ar_), 127.5 (C_Ar_), 127.3 (C_Ar_), 124.1 (C_Ar_), 124.0 (C_Ar_), 123.6 (C_Ar_), 117.3 (C_Ar_), 98.5 (C_Ar_), 42.1 (CH_2_), 40.1 (CH_2_), 20.7 (CH_3_) ppm; IR (KBr): ν 3356, 3197 (N–H), 3043 (H_Ar_), 1643 (C═O), 1612 (C═N), 1577, 1533, 1485, 1454 (C═C), 1296, 1226 (C–N), 817, 732 (Ar) cm^−1^; HRMS (ESI‐TOF) *m/z*: [M + H]^+^ calcd for C_19_H_19_ClN_3_O: 340.1211; found 340.1216.

*N*‐[2‐(7‐Chloroquinolin‐4‐ylamino)ethyl]‐4‐methoxybenzamide (**10d**) in 75% yield; TLC: (20% MeOH in AcOEt, *R*_f_): 0.43 (UV, I_2_); white solid; mp 267°C–270°C; ^1^H NMR (DMSO‐*d*_6_, 400 MHz): *δ* 8.48 (t, *J* = 5.5 Hz, 1H, NH), 8.41 (d, *J* = 5.4 Hz, 1H, H_Ar_), 8.19 (d, *J* = 9.0 Hz, 1H, H_Ar_), 7.83 (d, *J* = 8.9 Hz, 2H, H_Ar_), 7.79 (d, *J* = 2.2 Hz, 1H, H_Ar_), 7.44 (dd, *J* = 9.0, 2.3 Hz, 1H, H_Ar_), 7.39 (t, *J* = 5.2 Hz, 1H, NH), 6.99 (d, *J* = 8.9 Hz, 2H, H_Ar_), 6.62 (d, *J* = 5.5 Hz, 1H, H_Ar_), 3.81 (s, 3H, CH_3_), 3.56 (q, *J* = 6.1 Hz, 2H, CH_2_), 3.49–3.43 (m, 2H, CH_2_) ppm; ^13^C NMR (DMSO‐*d*_6_, 101 MHz): *δ* 166.4 (C═O), 161.5 (C_Ar_), 151.7 (C_Ar_), 150.0 (C_Ar_), 148.9 (C_Ar_), 133.2 (C_Ar_), 128.8 (C_Ar_), 127.3 (C_Ar_), 126.5 (C_Ar_), 124.0 (C_Ar_), 123.6 (C_Ar_), 117.3 (C_Ar_), 113.4 (C_Ar_), 98.5 (C_Ar_), 55.2 (CH_3_), 42.2 (CH_2_), 37.8 (CH_2_) ppm; IR (KBr): ν 3325, 3197 (N–H), 3059, 3016 (H_Ar_), 2962, 2935 (H_alkanic_), 1639 (C═O), 1608 (C═N), 1577, 1539, 1504, 1454 (C = C), 1300, 1257, 1222, 1180, 1165, 1134 (C–N/O), 848, 802, 759 (Ar) cm^−1^; HRMS (ESI‐TOF) *m/z*: [M + H]^+^ calcd for C_19_H_19_ClN_3_O_2_: 356.1160; found 356.1170.

*N*‐[2‐(7‐Chloroquinolin‐4‐ylamino)ethyl]‐3‐methoxybenzamide (**10e**) in 80% yield; TLC: (20% MeOH in AcOEt, *R*_f_): 0.46 (UV, I_2_); white solid; mp 260°C–263°C; ^1^H NMR (DMSO‐*d*_6_, 400 MHz): *δ* 8.60 (t, *J* = 5.3 Hz, 1H, NH), 8.42 (d, *J* = 5.4 Hz, 1H, H_Ar_), 8.20 (d, *J* = 9.0 Hz, 1H, H_Ar_), 7.79 (d, *J* = 2.1 Hz, 1H, H_Ar_), 7.46–7.42 (m, 2H, H_Ar_), 7.39–7.35 (m, 3H, H_Ar_, NH), 7.09 (dd, *J* = 7.9, 1.9 Hz, 1H, H_Ar_), 6.63 (d, *J* = 5.4 Hz, 1H, H_Ar_), 3.80 (s, 3H, CH_3_), 3.57 (q, *J* = 6.0 Hz, 2H, CH_2_), 3.51–3.45 (m, 2H, CH_2_) ppm; ^13^C NMR (DMSO‐*d*_6_, 101 MHz): δ 166.6 (C═O), 159.1 (C_Ar_), 151.7 (C_Ar_), 150.0 (C_Ar_), 148.9 (C_Ar_), 135.7 (C_Ar_), 133.2 (C_Ar_), 129.2 (C_Ar_), 127.3 (C_Ar_), 123.9 (C_Ar_), 123.6 (C_Ar_), 119.2 (C_Ar_), 117.3 (C_Ar_), 116.8 (C_Ar_), 112.4 (C_Ar_), 98.5 (C_Ar_), 55.1 (CH_3_), 40.0 (CH_2_), 37.9 (CH_2_) ppm; IR (KBr): ν 3344, 3186 (N–H), 2935 (H_alkanic_), 1647 (C═O), 1577, 1535, 1485, 1454 (C═C), 1296, 1226, 1134, 1053 (C–N/O), 817, 740 (Ar) cm^−1^; HRMS (ESI‐TOF) *m/z*: [M + H]^+^ calcd for C_19_H_19_ClN_3_O_2_: 356.1160; found 356.1148.

*N*‐[2‐(7‐Chloroquinolin‐4‐ylamino)ethyl]‐3,4‐dimethoxybenzamide (**10 f**) in 70% yield; TLC: (20% MeOH in AcOEt, *R*_f_): 0.4 (UV, I_2_); white solid; mp 249°C–251°C; ^1^H NMR (DMSO‐*d*_6_, 400 MHz): δ 8.60 (t, *J* = 5.6 Hz, 1H, NH), 8.41 (d, *J* = 5.4 Hz, 1H, H_Ar_), 8.20 (d, *J* = 9.1 Hz, 1H, H_Ar_), 7.79 (d, *J* = 2.2 Hz, 1H, H_Ar_), 7.51–7.47 (m, 2H, H_Ar_), 7.45–7.44 (m, 2H, H_Ar_, NH), 7.02 (d, *J* = 8.5 Hz, 1H, H_Ar_), 6.65 (d, *J* = 5.5 Hz, 1H, H_Ar_), 3.80 (s, 3H, CH_3_), 3.79 (s, 3H, CH_3_), 3.54 (q, *J* = 6.2 Hz, 2H, CH_2_), 3.46 (q, *J* = 6.4 Hz, 2H, CH_2_) ppm; ^13^C NMR (DMSO‐*d*_6_, 101 MHz): *δ* 166.5 (C═O), 151.9 (C_Ar_), 151.3 (C_Ar_), 150.1 (C_Ar_), 149.0 (C_Ar_), 148.2 (C_Ar_), 133.4 (C_Ar_), 127.5 (C_Ar_), 126.6 (C_Ar_), 124.2 (C_Ar_), 123.9 (C_Ar_), 120.4 (C_Ar_), 117.4 (C_Ar_), 110.9 (C_Ar_), 110.6 (C_Ar_), 98.7 (C_Ar_), 55.6 (CH_3_), 55.5 (CH_3_), 42.1 (CH_2_), 37.9 (CH_2_) ppm; IR (KBr): ν 3290, 3209 (N–H), 3066 (H_Ar_), 2931 (H_alkanic_), 1643 (C═O), 1577, 1543, 1508, 1458 (C = C), 1300, 1273, 1222, 1188, 1134, 1022 (C–N/O), 817, 767 (Ar) cm^−1^; HRMS (ESI‐TOF) *m/z*: [M + H]^+^ calcd for C_20_H_21_ClN_3_O_3_: 386.1266; found 386.1256.

4‐Chloro‐*N*‐[2‐(7‐chloroquinolin‐4‐ylamino)ethyl]benzamide (**10g**) in 98% yield; TLC: (25% MeOH in AcOEt, *R*_f_): 0.43 (UV, I_2_); white solid; mp 280°C–282°C (lit [24]. mp 235°C); ^1^H NMR (DMSO‐*d*_6_, 400 MHz): *δ* 8.70 (t, *J* = 5.5 Hz, 1H, NH), 8.41 (d, *J* = 5.4 Hz, 1H, H_Ar_), 8.19 (d, *J* = 9.0 Hz, 1H, H_Ar_), 7.87 (d, *J* = 8.6 Hz, 2H, H_Ar_), 7.79 (d, *J* = 2.2 Hz, 1H, H_Ar_), 7.53 (d, *J* = 8.7 Hz, 2H, H_Ar_), 7.44 (dd, *J* = 9.0, 2.3 Hz, 1H, H_A_), 7.37 (t, *J* = 5.4 Hz, 1H, NH), 6.61 (d, *J* = 5.4 Hz, 1H, H_Ar_), 3.57 (q, *J* = 6.0 Hz, 2H, CH_2_), 3.49 (q, *J* = 6.3 Hz, 2H, CH_2_) ppm; ^13^C NMR (DMSO‐*d*_6_, 101 MHz): *δ* 165.7 (C═O), 151.7 (C_Ar_), 149.9 (C_Ar_), 148.9 (C_Ar_), 135.9 (C_Ar_), 133.2 (C_Ar_), 133.0 (C_Ar_), 128.9 (C_Ar_), 128.2 (C_Ar_), 127.3 (C_Ar_), 123.9 (C_Ar_), 123.6 (C_Ar_), 117.3 (C_Ar_), 98.5 (C_Ar_), 41.9 (CH_2_), 37.9 (CH_2_) ppm; HRMS (ESI‐TOF) *m/z*: [M + H]^+^ calcd for C_18_H_16_Cl_2_N_3_O: 360.0665; found 360.0648.

3‐Chloro‐*N*‐[2‐(7‐chloroquinolin‐4‐ylamino)ethyl]benzamide (**10h**) in 96% yield; TLC: (25% MeOH in AcOEt, *R*_f_): 0.46 (UV, I_2_); white solid; mp 278°C–280°C; ^1^H NMR (DMSO‐*d*_6_, 500 MHz): *δ* 8.73 (t, *J* = 5.7 Hz, 1H, NH), 8.41 (d, *J* = 5.3 Hz, 1H, H_Ar_), 8.20 (d, *J* = 9.0 Hz, 1H, H_Ar_), 7.88–7.87 (m, 1H, H_Ar_), 7.81–7.79 (m, 2H, H_Ar_), 7.59 (dd, *J* = 7.5, 1.7 Hz, 1H, H_Ar_), 7.50 (t, *J* = 7.9 Hz, 1H, H_Ar_), 7.45 (dd, *J* = 9.0, 2.3 Hz, 1H, H_Ar_), 7.37 (t, *J* = 5.5 Hz, 1H, NH), 6.62 (d, *J* = 5.4 Hz, 1H, H_Ar_), 3.58–3.55 (m, 2H, CH_2_), 3.51–3.48 (m, 2H, CH_2_) ppm; ^13^C NMR (DMSO‐*d*_6_, 126 MHz): *δ* 165.3 (C═O), 151.7 (C_Ar_), 150.0 (C_Ar_), 148.9 (C_Ar_), 136.3 (C_Ar_), 133.0 (C_Ar_), 130.8 (C_Ar_), 130.1 (C_Ar_), 127.3 (C_Ar_), 126.8 (C_Ar_), 125.7 (C_Ar_), 124.0 (C_Ar_), 123.7 (C_Ar_), 117.3 (C_Ar_), 98.5 (C_Ar_), 42.8 (CH_2_), 37.9 (CH_2_) ppm; IR (KBr): ν 3363, 3194 (N–H), 3109, 3047 (H_Ar_), 2974, 2935, 2881 (H_alkanic_), 1643 (C═O), 1577, 1535, 1454 (C═C), 1296, 1265, 1226, 1165, 1138, 1114, 1083 (C–N), 848, 794, 736 (Ar) cm^−1^; HRMS (ESI‐TOF) *m/z*: [M + H]^+^ calcd for C_18_H_16_Cl_2_N_3_O: 360.0665; found 360.0656.

*N*‐[2‐(7‐Chloroquinolin‐4‐ylamino)ethyl]‐4‐fluorobenzamide (**10i**) in 92% yield; TLC: (30% MeOH in AcOEt, *R*_f_): 0.4 (UV, I_2_); white solid; mp > 300°C; ^1^H NMR (DMSO‐*d*_6_, 500 MHz, 70°C): *δ* 8.56 (s, 1H, NH), 8.42 (d, *J* = 4.9 Hz, 1H, H_Ar_), 8.19 (d, *J* = 8.9 Hz, 1H, H_Ar_), 7.93–7.91 (m, 2H, H_Ar_), 7.79 (s, 1H, H_Ar_), 7.43 (d, *J* = 8.8 Hz, 1H, H_Ar_), 7.28–7.25 (m, 3H, H_Ar_, NH), 6.61 (d, *J* = 5.0 Hz, 1H, H_Ar_), 3.59 (q, *J* = 6.6 Hz, 2H, CH_2_), 3.50 (q, *J* = 6.5 Hz, 2H, CH_2_) ppm; ^13^C NMR (DMSO‐*d*_6_, 126 MHz, 70°C): *δ* 166.5 (C═O), 164.4 (d, ^1^ *J* = 249.3 Hz, C_Ar_), 152.3 (C_Ar_), 150.6 (C_Ar_), 149.6 (C_Ar_), 133.9 (C_Ar_), 131.5 (d, ^4^ *J* = 2.9 Hz, C_Ar_), 130.3 (d, ^3^ *J* = 8.9 Hz, C_Ar_), 128.0 (C_Ar_), 124.5 (C_Ar_), 124.2 (C_Ar_), 118.0 (C_Ar_), 115.6 (d, ^2^ *J* = 21.6 Hz, C_Ar_), 99.2 (C_Ar_), 42.7 CH_2_), 38.6 (CH_2_) ppm; IR (KBr): ν 3321, 3182 (N–H), 3016 (H_Ar_), 2974, 2931 (H_alkanic_), 1647 (C═O), 1608 (C═N), 1570, 1535, 1504, 1454 (C═C), 1296, 1226, 1161 (C–N), 848, 798, 756, 682 (Ar) cm^−1^; HRMS (ESI‐TOF) *m/z*: [M + H]^+^ calcd for C_18_H_16_ClFN_3_O: 344.0960; found 344.0959.

*N*‐[2‐(7‐Chloroquinolin‐4‐ylamino)ethyl]‐4‐nitrobenzamide (**10j**) in 60% yield; TLC: (30% MeOH in AcOEt, *R*_f_): 0.56 (UV, I_2_); yellow solid; mp 243°C–245°C (lit [24]. mp 217°C); ^1^H NMR (DMSO‐*d*_6_, 400 MHz): *δ* 9.02 (t, *J* = 5.5 Hz, 1H, NH), 8.42 (d, *J* = 5.4 Hz, 1H, H_Ar_), 8.32 (d, *J* = 8.9 Hz, 2H, H_Ar_), 8.20 (d, *J* = 9.1 Hz, 1H, H_Ar_), 8.07 (d, *J* = 8.9 Hz, 2H, H_Ar_), 7.79 (d, *J* = 2.2 Hz, 1H, H_Ar_), 7.50–7.45 (m, 2H, H_A_, NH), 6.63 (d, *J* = 5.5 Hz, 1H, H_A_), 3.57 (q, *J* = 6.2 Hz, 2H, CH_2_), 3.51 (q, *J* = 5.8 Hz, 2H, CH_2_) ppm; ^13^C NMR (DMSO‐*d*_6_, 101 MHz): *δ* 165.1 (C═O), 151.9 (C_Ar_), 150.0 (C_Ar_), 149.1 (C_Ar_), 149.0 (C_Ar_), 139.9 (C_Ar_), 133.4 (C_Ar_), 128.7 (C_Ar_), 127.5 (C_Ar_), 124.1 (C_Ar_), 123.9 (C_Ar_), 123.5 (C_Ar_), 117.4 (C_Ar_), 98.6 (C_Ar_), 41.6 (CH_2_), 38.1 (CH_2_) ppm; HRMS (ESI‐TOF) *m/z*: [M + H]^+^ calcd for C_18_H_16_ClN_4_O_3_: 371.0905; found 371.0905.

*N*‐[2‐(7‐Chloroquinolin‐4‐ylamino)ethyl]acetamide (**10k**) in 80% yield; TLC: (30% MeOH in AcOEt, *R*_f_): 0.4 (UV, I_2_); white solid; mp 224°C–225°C; ^1^H NMR (DMSO‐*d*_6_, 500 MHz): *δ* 8.39 (d, *J* = 5.4 Hz, 1H, H_Ar_), 8.16 (d, *J* = 9.0 Hz, 1H, H_Ar_), 8.11 (s, 1H, NH), 7.79 (d, *J* = 2.2 Hz, 1H, H_Ar_), 7.45 (dd, *J* = 8.9, 2.2 Hz, 1H, H_Ar_), 7.40 (s, 1H, NH), 6.53 (d, *J* = 5.4 Hz, 1H, H_Ar_), 3.33–3.32 (m, 4H, 2 × CH_2_), 1.83 (s, 3H, CH_3_) ppm; ^13^C NMR (DMSO‐*d*_6_, 126 MHz): *δ* 170.2 (C═O), 151.9 (C_Ar_), 150.1 (C_Ar_), 149.0 (C_Ar_), 133.5 (C_Ar_), 127.5 (C_Ar_), 124.2 (C_Ar_), 123.9 (C_Ar_), 117.4 (C_Ar_), 98.6 (C_Ar_), 42.3 (CH_2_), 37.4 (CH_2_), 22.6 (CH_3_) ppm; IR (KBr): ν 3278, 3196 (N–H), 3035 (H_Ar_), 2927 (H_alkanic_), 1662 (C═O), 1585, 1566 (C═C), 1292, 1226, 1138 (C–N), 848, 802 (Ar) cm^−1^; HRMS (ESI‐TOF) *m/z*: [M + H]^+^ calcd for C_13_H_15_ClN_3_O: 264.08982; found 264.0895.

*N*‐[2‐(7‐Chloroquinolin‐4‐ylamino)ethyl]isobutyramide (**10l**) in 80% yield; TLC: (30% MeOH in AcOEt, *R*_f_): 0.4 (UV, I_2_); white solid; mp 234°C–236°C; ^1^H NMR (DMSO‐*d*_6_, 500 MHz): *δ* 8.40 (d, *J* = 5.4 Hz, 1H, H_Ar_), 8.16 (d, *J* = 9.0 Hz, 1H, H_Ar_), 7.98 (s, 1H, NH), 7.78 (d, *J* = 2.2 Hz, 1H, H_Ar_), 7.46 (dd, *J* = 9.0, 2.2 Hz, 1H, H_Ar_), 7.38 (s, 1H, NH), 6.56 (d, *J* = 5.5 Hz, 1H, H_Ar_), 3.33–3.32 (m, 4H, 2 × CH_2_), 2.34 (hept, *J* = 6.9 Hz, 1H, CH), 0.99 (d, *J* = 6.9 Hz, 6H, 2 × CH_3_) ppm; ^13^C NMR (DMSO‐*d*_6_, 126 MHz): *δ* 176.9 (C═O), 151.9 (C_Ar_), 150.1 (C_Ar_), 149.0 (C_Ar_), 133.4 (C_Ar_), 127.5 (C_Ar_), 124.2 (C_Ar_), 123.8 (C_Ar_), 117.4 (C_Ar_), 98.6 (C_Ar_), 42.3 (CH_2_), 37.2 (CH_2_), 34.1 (CH), 19.5 (CH_3_) ppm; IR (KBr): ν 3352, 3313 (N–H), 2974 (H_alkanic_), 1624 (C═O), 1585, 1550 (C═C), 1249, 1141, 1099 (C–N), 806, 698 (Ar) cm^−1^; HRMS (ESI‐TOF) *m/z*: [M + H]^+^ calcd for C_15_H_19_ClN_3_O: 292.1211; found 292.1224.

#### 4.1.4 Experimental Procedures for Synthesis of 2‐(7‐chloroquinolin‐4‐ylamino/oxy)ethyl Benzoates (11–12)

7‐Chloro‐*N*‐(2‐chloroethyl)quinolin‐4‐amine and 7‐chloro‐4‐(2‐chloroethoxy)quinoline were obtained in yields of 99% and 86%, respectively, according to a method previously described by Fiss et al. [16]. Compounds **11–12** were synthesized according to the following method. A mixture of potassium benzoate (1.2 mmol), 7‐chloro‐*N*‐(2‐chloroethyl)quinolin‐4‐amine or 7‐chloro‐4‐(2‐chloroethoxy)quinoline (1 mmol), and anhydrous DMF (2 mL) was stirred at 100°C for 2 h. Afterward, the reactional mixture was cooled to room temperature, added to a beaker containing ice water (10 mL), and left in a freezer until freezing point. Then, the precipitate was filtered with excess ice water. 2‐(7‐Chloroquinolin‐4‐ylamino)ethyl (**11**) and 2‐(7‐chloroquinolin‐4‐yloxy)ethyl (**12)** benzoates were obtained in yields of 80% and 86%, respectively. Full NMR, IR and HRMS spectra for compounds **11–12** are available in Supporting Information.

2‐(7‐Chloroquinolin‐4‐ylamino)ethyl benzoate (**11**) in 80% yield; white solid; mp 150°C–152°C; ^1^H NMR (DMSO‐*d*_6_, 500 MHz): *δ* 8.43 (d, *J* = 5.4 Hz, 1H, H_Ar_), 8.26 (d, *J* = 9.0 Hz, 1H, H_Ar_), 7.96 (d, *J* = 7.2 Hz, 2H, H_Ar_), 7.80 (d, *J* = 2.2 Hz, 1H, H_Ar_), 7.63 (t, *J* = 7.4 Hz, 1H, H_Ar_), 7.53 (t, *J* = 5.6 Hz, 1H, NH), 7.50 (t, *J* = 7.8 Hz, 2H, H_Ar_), 7.45 (dd, *J* = 9.0, 2.2 Hz, 1H, H_Ar_), 6.66 (d, *J* = 5.5 Hz, 1H, H_Ar_), 4.52 (t, *J* = 5.5 Hz, 2H, CH_2_), 3.72 (q, *J* = 5.5 Hz, 2H, CH_2_) ppm; ^13^C NMR (DMSO‐*d*_6_, 126 MHz): *δ* 165.8 (C═O), 151.9 (C_Ar_), 150.1 (C_Ar_), 149.0 (C_Ar_), 133.5 (C_Ar_), 133.3 (C_Ar_), 129.6 (C_Ar_), 129.2 (C_Ar_), 128.7 (C_Ar_), 127.5 (C_Ar_), 124.2 (C_Ar_), 124.0 (C_Ar_), 117.4 (C_Ar_), 98.9 (C_Ar_), 62.7 (CH_2_), 41.2 (CH_2_) ppm; IR (KBr): ν 3244 (N–H), 3062 (H_Ar_), 2962 (H_alkanic_), 1716 (C = O), 1577, 1539, 1450 (C═C), 1280, 1249, 1111, 1026 (C–N/O), 848, 709 (Ar) cm^−1^; HRMS (ESI‐TOF) *m/z*: [M + H]^+^ calcd for C_18_H_16_ClN_2_O_2_: 327.0895; found 327.0886.

2‐(7‐Chloroquinolin‐4‐yloxy)ethyl benzoate (**12**) in 86% yield; white solid; mp 121°C–123°C; ^1^H NMR (DMSO‐*d*_6_, 500 MHz): *δ* 8.77 (d, *J* = 5.2 Hz, 1H, H_Ar_), 8.13 (d, *J* = 8.9 Hz, 1H, H_Ar_), 7.99–7.98 (m, 2H, H_Ar_), 7.96–7.96 (m, 1H, H_Ar_), 7.66–7.63 (m, 1H, H_Ar_), 7.57 (dd, *J* = 8.9, 2.1 Hz, 1H, H_Ar_), 7.53–7.49 (m, 2H, H_Ar_), 7.15 (d, *J* = 5.3 Hz, 1H, H_Ar_), 4.79–4.78 (m, 2H, CH_2_), 4.65–4.63 (m, 2H, CH_2_) ppm; ^13^C NMR (DMSO‐*d*_6_, 126 MHz): *δ* 165.7 (C═O), 160.6 (C_Ar_), 153.1 (C_Ar_), 149.1 (C_Ar_), 134.5 (C_Ar_), 133.5 (C_Ar_), 129.4 (C_Ar_), 129.2 (C_Ar_), 128.8 (C_Ar_), 127.3 (C_Ar_), 126.4 (C_Ar_), 123.7 (C_Ar_), 119.3 (C_Ar_), 102.3 (C_Ar_), 67.0 (CH_2_), 62.8 (CH_2_) ppm; IR (KBr): ν 1716 (C═O), 1612 (C═N), 1573, 1496 (C═C), 1280, 1153, 1114, 1072 (C–N/O), 813, 709 (Ar) cm^−1^; HRMS (ESI‐TOF) *m/z*: [M + H]^+^ calcd for C_18_H_15_ClNO_3_: 328.0735; found 328.0725.

### 4.2 Pharmacological Assays

#### 4.2.1 In Silico/Vitro Antiplasmodial Evaluation

In silico antiplasmodial activity for 4‐alkoxy/alkylamino‐7‐chloroquinolines (**2–9**), *N*‐2‐(7‐chloroquinolin‐4‐ylamino)ethyl amides (**10a–l**), 2‐(7‐chloroquinolin‐4‐ylamino/oxy)ethyl benzoates (**11–12**) and CQ against 3D7 and K1 strains was calculated using the AmIActive program “https://amiactive.ccen.ufpb.br (accessed on 15 March 2026)” [19].

*In vitro* antiplasmodial assays of 4‐alkoxy/alkylamino‐7‐chloroquinolines (**2–9**), *N*‐2‐(7‐chloroquinolin‐4‐ylamino)ethyl amides (**10a–l**), 2‐(7‐chloroquinolin‐4‐ylamino/oxy)ethyl benzoates (**11–12**) and CQ were performed at the Department of Microbiology, Immunology and Parasitology (UNIFESP). *P. falciparum* strains 3D7 (CQ‐sensitive) and K1 (CQ‐resistant) were maintained in human O^+^ red blood cells (RBCs) and RPMI‐1640 culture medium supplemented with 2.0 g·L^–1^ NaHCO_3_, 5.98 g·L^–1^ HEPES (pH 7.4), 2.0 g·L^–1^ d‐glucose, 50 mg·L^–1^ hypoxanthine, 25 mg·L^–1^ gentamicin, and 5 g·L^–1^ AlbuMAX II. Cultures were maintained in T25 flasks with daily changes of the culture medium and incubated under a gas mixture (90% N_2_, 5% CO_2_, and 5% O_2_) at 37°C for parasite growth [25]. Before antiplasmodial evaluation, the cultures were treated with a sterile 5% (w/v) sorbitol solution to enrich parasites at the ring stage [26]. For the growth plate experiment, a twofold serial dilution of 10‐fold concentrated compounds was prepared (final volume of 20 µL), with an initial concentration of 1 µM. A parasite inoculum was then prepared at 0.5% parasitemia and 2% hematocrit using appropriate volumes of RBCs and culture medium, and 180 µL of this cell suspension was added to each well. Plates were incubated under growth conditions for 72 h. After the incubation period, 180 µL of the supernatant was removed, followed by the addition of 100 µL of 1 × PBS (116 mM NaCl, 10 mM NaH_2_PO_4_, and 3 mM KH_2_PO_4_) and resuspension of the cells. Immediately thereafter, 100 µL of lysis buffer (20 mM Tris base, 5 mM EDTA, 0.0008% (v/v) Triton X‐100, 0.008% (w/v) saponin, pH 8.0) containing 0.002% (v/v) SYBR Green I was added. The plates were incubated in the dark at room temperature for 30 min. Fluorescence corresponding to parasite density (excitation at 475 nm, emission at 500–550 nm) was measured using a GloMax Explorer Multimode Microplate Reader (Promega, Madison, WI, USA) [27]. Uninfected and infected RBCs without treatment were used as negative and positive controls, respectively, for parasite growth. The concentration required to inhibit 50% of parasite growth (IC_50_) was determined by nonlinear regression analysis of the concentration–response curve using GraphPad Prism version 8.0.1 for Windows (GraphPad Software, San Diego, CA, USA).

#### 4.2.2 In Silico/Vitro Toxicity Evaluation

*In silico* toxicity for 4‐alkoxy/alkylamino‐7‐chloroquinolines (**6–9**) and *N*‐2‐(7‐chloroquinolin‐4‐ylamino)ethyl amides (**10a–l**) was calculated using the pkCSM program “https://biosig.lab.uq.edu.au/pkcsm/prediction (accessed on 22 March 2026)” [22].

*In vitro* cytotoxicity assays of 4‐alkoxy/alkylamino‐7‐chloroquinolines (**6–9**) and *N*‐2‐(7‐chloroquinolin‐4‐ylamino)ethyl amides (**10a–l**) were performed at the Department of Microbiology, Immunology and Parasitology (UNIFESP). Based on a method previously described by Aguiar et al. [28], HepG2 human hepatocellular carcinoma cells were maintained in RPMI‐1640 culture medium supplemented with 2 g·L^–1^ NaHCO_3_, 5.98 g·L^–1^ HEPES (pH 7.4), 2 g·L^–1^ d‐glucose, 10 mg·L^–1^ hypoxanthine, 25 mg·L^–1^ gentamicin, and 10% (v/v) fetal bovine serum (FBS). HepG2 cells were cultured in T75 flasks, with the culture medium changed twice a week, and maintained in a humidified incubator with 5% CO_2_ at 37°C. When the cells reached 80% confluence in a T75 flask, 7 mL of 0.05% (1×) trypsin–EDTA was added to induce cell detachment. An inoculum was prepared from this cell suspension by seeding 180 µL containing 3 × 10^4^ cells per well. The culture plate was then incubated under growth conditions for 24 h to allow cell adhesion. The following day, a twofold serial dilution of 10‐fold concentrated compounds was prepared, with an initial concentration of 100 µM. Then, 20 µL of this dilution was added to each well, and the plate was incubated under growth conditions for 72 h. After the incubation period, 40 µL of a 100‐fold concentrated resazurin solution was added to each well (final concentration of 0.015 mg·mL^–1^), followed by incubation under growth conditions for approximately 4 h. Fluorescence corresponding to cell density (excitation at 560 nm, emission at 590 nm) was measured using a GloMax Explorer Multimode Microplate Reader (Promega, Madison, WI, USA). HepG2 cells without treatment or culture medium were used as positive and negative controls for cell growth. The concentration required to inhibit 50% of cell growth (CC_50_) was determined by nonlinear regression analysis of the concentration–response curve using GraphPad Prism version 8.0.1 for Windows (GraphPad Software, San Diego, CA, USA).

### 4.3 3D Qualitative SAR Model

Based on a method previously described by Vinter et al. [23], a qualitative SAR model was built using the Activity Atlas module available in Forge v10.6.0 for *N*‐2‐(7‐chloroquinolin‐4‐ylamino)ethyl amides (**10a–l**) and CQ. Before model generation, a ligand‐based pharmacophore hypothesis was generated using FieldTemplater. This tool generates an ensemble of conformations and uses electrostatic and shape fields to find patterns within the active molecules, providing a possible bioactive conformation.

## Funding

The Article Processing Charge for the publication of this research was funded by the Coordenação de Aperfeiçoamento de Pessoal de Nível Superior ‐ Brasil (CAPES) ROR identifier: 00x0ma614).

## Conflicts of Interest

The authors declare no conflicts of interest.

## Supporting information


Supporting File S1

Supporting File S2

## Data Availability

The data that support the findings of this study are available in the supporting material of this article.
