# Supplementary material for: Rational Design of N‐2‐(7‐Chloroquinolin‐4‐ylamino)ethyl Amides Active Against Chloroquine‐Resistant Plasmodium falciparum
Source: Arch Pharm (Weinheim). 2026 Oct 4;359(10):e70350. doi: 10.1002/ardp.70350 (PMC13635770; doi:10.1002/ardp.70350)
Supplement: Supplementary file 1 [file ARDP-359-e70350-s001.pdf]

Arch Pharm
Archiv der Pharmazie

Rational Design of *N*-(2-(7-Chloroquinolin-4-ylamino)ethyl Amides Active against Chloroquine-resistant *Plasmodium falciparum*

Everton P. Silva[1], Camila S. Barbosa[2], Anna C. C. Aguiar [2], Ramon G. Oliveira[3], Thiago A. M. Brito[4], Marcelo F. R. Silva[4], Helivaldo D. S. Souza[1], Petrônio F. Athayde-Filho[1], Gabriela F. Fiss[1,*]

1 Laboratório de Pesquisa em Bioenergia e Síntese Orgânica (LPBS), Department of Chemistry, Universidade Federal da Paraíba (UFPB), João Pessoa, Brazil
2 Department of Microbiology, Immunology and Parasitology, Universidade Federal de São Paulo (UNIFESP), São Paulo, Brazil
3 Laboratório Nacional de Biociências (LNBio), Centro Nacional de Pesquisa em Energia e Materiais (CNPEM), Campinas, Brazil
4 Laboratório Multiusuário de Caracterização e Análises (LMCA), Instituto de Pesquisa em Fármacos e Medicamentos (IPeFarM), UFPB, João Pessoa, Brazil

*Correspondence:
Professor, Gabriela F. Fiss, LPBS, Department of Chemistry, UFPB, João Pessoa 58051-900, Brazil
Email: gffiss@gmail.com, gff@academico.ufpb.br

ARCH PHARM
Archiv der Pharmazie

Supplemental Material: Novel Compounds and Biological Screening Results

Compd.	InChI	Biological Activity (nM)		
		IC_{50}^{3D7} ± SD [a]	IC_{50}^{K1} ± SD [a]	CC_{50} HepG2 [b]
**10b**	InChI=1S/C19H18ClN3O/c1-13-2-4-14(5-3-13)19(24)23-11-10-22-17-8-9-21-18-12-15(20)6-7-16(17)18/h2-9,12H,10-11H2,1H3,(H,21,22)(H,23,24)	207 ± 98	237 ± 126	>12500
**10c**	InChI=1S/C19H18ClN3O/c1-13-3-2-4-14(11-13)19(24)23-10-9-22-17-7-8-21-18-12-15(20)5-6-16(17)18/h2-8,11-12H,9-10H2,1H3,(H,21,22)(H,23,24)	152 ± 66	78 ± 21	>12500
**10d**	InChI=1S/C19H18ClN3O2/c1-25-15-5-2-13(3-6-15)19(24)23-11-10-22-17-8-9-21-18-12-14(20)4-7-16(17)18/h2-9,12H,10-11H2,1H3,(H,21,22)(H,23,24)	208 ± 74	96 ± 26	>25000
**10e**	InChI=1S/C19H18ClN3O2/c1-25-15-4-2-3-13(11-15)19(24)23-10-9-22-17-7-8-21-18-12-14(20)5-6-16(17)18/h2-8,11-12H,9-10H2,1H3,(H,21,22)(H,23,24)	219 ± 76	108 ± 11	>50000
**10f**	InChI=1S/C20H20ClN3O3/c1-26-18-6-3-13(11-19(18)27-2)20(25)24-10-9-23-16-7-8-22-17-12-14(21)4-5-15(16)17/h3-8,11-12H,9-10H2,1-2H3,(H,22,23)(H,24,25)	210 ± 35	160.5 ± 0.7	>50000
**10h**	InChI=1S/C18H15Cl2N3O/c19-13-3-1-2-12(10-13)18(24)23-9-8-22-16-6-7-21-17-11-14(20)4-5-15(16)17/h1-7,10-11H,8-9H2,(H,21,22)(H,23,24)	109 ± 47	72 ± 23	>50000
**10i**	InChI=1S/C18H15ClFN3O/c19-13-3-6-15-16(7-8-21-17(15)11-13)22-9-10-23-18(24)12-1-4-14(20)5-2-12/h1-8,11H,9-10H2,(H,21,22)(H,23,24)	258 ± 95	150 ± 50	>12500
**10k**	InChI=1S/C13H14ClN3O/c1-9(18)15-6-7-17-12-4-5-16-13-8-10(14)2-3-11(12)13/h2-5,8H,6-7H2,1H3,(H,15,18)(H,16,17)	247 ± 90	143 ± 23	>100000
**10l**	InChI=1S/C15H18ClN3O/c1-10(2)15(20)19-8-7-18-13-5-6-17-14-9-11(16)3-4-12(13)14/h3-6,9-10H,7-8H2,1-2H3,(H,17,18)(H,19,20)	555 ± 169	183 ± 6	>100000
**11**	InChI=1S/C18H15ClN2O2/c19-14-6-7-15-16(8-9-20-17(15)12-14)21-10-11-23-18(22)13-4-2-1-3-5-13/h1-9,12H,10-11H2,(H,20,21)	>1000	–	–
**12**	InChI=1S/C18H14ClNO3/c19-14-6-7-15-16(12-14)20-9-8-17(15)22-10-11-23-18(21)13-4-2-1-3-5-13/h1-9,12H,10-11H2	>1000	–	–

[a] 50% inhibitory concentration ± standard deviation against *P. falciparum* strains 3D7 (CQ-sensitive) and K1 (CQ-resistant), determined based on a method previously described by Annunciato *et al.*

[b] 50% cytotoxicity concentration on HepG2 human hepatocellular carcinoma cells, determined based on a method previously described by Annunciato *et al.*

Y. Annunciato, E. M. Silva, J. E. Araújo, L. N. Matinez, S. Santana, E. A. Workneh, L. C. S. Alvarez, M. L. Magalhães, A. O. Andrade, W. J. Costa, G. Campolina, M. N. Santana, C. S. Barbosa, E. P. M. L. Peres, C. S. Moura, N. A. C. Santos, A. S. Bastos, R. R. M. Barros, M. L. Gazarini, J. F. Medeiros, C. B. G. Teles, F. T. M. Costa, A. C. Alves, D. B. Pereira, J. Pinto, P. V. L. Cravo, R. V. C. Guido, M. Prudêncio, M. S. Araujo, A. G. Corrêa, G. C. Cassiano, A. C. C. Aguiar, Evaluation of the activity of 4-quinolones against multi-life stages of *Plasmodium* spp., *ACS Omega* **2025**, *10*, 54850−54863. https://doi.org/10.1021/acsomega.5c08663.
