# Supplementary material for: Rational Design of N‐2‐(7‐Chloroquinolin‐4‐ylamino)ethyl Amides Active Against Chloroquine‐Resistant Plasmodium falciparum
Source: Arch Pharm (Weinheim). 2026 Oct 4;359(10):e70350. doi: 10.1002/ardp.70350 (PMC13635770; doi:10.1002/ardp.70350)
Supplement: Supplementary file 2 [file ARDP-359-e70350-s002.pdf]

WILEY-VCH

Archiv der Pharmazie

Rational Design of *N*-2-(7-Chloroquinolin-4-ylamino)ethyl Amides Active against Chloroquine-resistant *Plasmodium falciparum*

Everton P. Silva[1], Camila S. Barbosa[2], Anna C. C. Aguiar[2], Ramon G. Oliveira[3], Thiago A. M. Brito[4], Marcelo F. R. Silva[4], Helivaldo D. S. Souza[1], Petrônio F. Athayde-Filho[1], Gabriela F. Fiss[1,*]

1 Laboratório de Pesquisa em Bioenergia e Síntese Orgânica (LPBS), Department of Chemistry, Universidade Federal da Paraíba (UFPB), João Pessoa, Brazil
2 Department of Microbiology, Immunology and Parasitology, Universidade Federal de São Paulo (UNIFESP), São Paulo, Brazil
3 Institute of Chemistry, Universidade Estadual de Campinas (UNICAMP), Campinas, Brazil
4 Laboratório Multiusuário de Caracterização e Análises (LMCA), Instituto de Pesquisa em Fármacos e Medicamentos (IPeFarM), UFPB, João Pessoa, Brazil

*Correspondence:
Professor, Gabriela F. Fiss, LPBS, Department of Chemistry, UFPB, João Pessoa 58051-900, Brazil
Email: gffiss@gmail.com, gff@academico.ufpb.br

WILEY-VCH

Supporting Information

FIGURE S1 ^{1}H NMR spectrum (DMSO-d_6, 400 MHz) of compound **10a**.
FIGURE S2 ^{13}C NMR APT spectrum (DMSO-d_6, 101 MHz) of compound **10a**.
FIGURE S3 HRMS spectrum (ESI) of compound **10a**.
FIGURE S4 HPLC chromatogram of compound **10a**.
FIGURE S5 ^{1}H NMR spectrum (DMSO-d_6, 400 MHz) of compound **10b**.
FIGURE S6 ^{13}C NMR spectrum (DMSO-d_6, 101 MHz) of compound **10b**.
FIGURE S7 IR spectrum (KBr) of compound **10b**.
FIGURE S8 HRMS spectrum (ESI) of compound **10b**.
FIGURE S9 HPLC chromatogram of compound **10b**.
FIGURE S10 ^{1}H NMR spectrum (DMSO-d_6, 400 MHz) of compound **10c**.
FIGURE S11 ^{13}C NMR spectrum (DMSO-d_6, 101 MHz) of compound **10c**.
FIGURE S12 IR spectrum (KBr) of compound **10c**.
FIGURE S13 HRMS spectrum (ESI) of compound **10c**.
FIGURE S14 HPLC chromatogram of compound **10c**.
FIGURE S15 ^{1}H NMR spectrum (DMSO-d_6, 400 MHz) of compound **10d**.
FIGURE S16 ^{13}C NMR spectrum (DMSO-d_6, 101 MHz) of compound **10d**.
FIGURE S17 IR spectrum (KBr) of compound **10d**.
FIGURE S18 HRMS spectrum (ESI) of compound **10d**.
FIGURE S19 HPLC chromatogram of compound **10d**.
FIGURE S20 ^{1}H NMR spectrum (DMSO-d_6, 400 MHz, DMSO-d_6) of compound **10e**.
FIGURE S21 ^{13}C NMR spectrum (DMSO-d_6, 101 MHz, DMSO-d_6) of compound **10e**.
FIGURE S22 IR spectrum (KBr) of compound **10e**.
FIGURE S23 HRMS spectrum (ESI) of compound **10e**.
FIGURE S24 HPLC chromatogram of compound **10e**.
FIGURE S25 ^{1}H NMR spectrum (DMSO-d_6, 400 MHz) of compound **10f**.
FIGURE S26 ^{13}C NMR spectrum (DMSO-d_6, 101 MHz) of compound **10f**.
FIGURE S27 IR spectrum (KBr) of compound **10f**.
FIGURE S28 HRMS spectrum (ESI) of compound **10f**.
FIGURE S29 HPLC chromatogram of compound **10f**.
FIGURE S30 ^{1}H NMR spectrum (DMSO-d_6, 400 MHz) of compound **10g**.
FIGURE S31 ^{13}C NMR spectrum (DMSO-d_6, 101 MHz) of compound **10g**.
FIGURE S32 HRMS spectrum (ESI) of compound **10g**.
FIGURE S33 HPLC chromatogram of compound **10g**.
FIGURE S34 ^{1}H NMR spectrum (DMSO-d_6, 500 MHz) of compound **10h**.
FIGURE S35 ^{13}C NMR spectrum (DMSO-d_6, 126 MHz) of compound **10h**.
FIGURE S36 IR spectrum (KBr) of compound **10h**.
FIGURE S37 HRMS spectrum (ESI) of compound **10h**.
FIGURE S38 HPLC chromatogram of compound **10h**.
FIGURE S39 ^{1}H NMR spectrum (DMSO-d_6, 500 MHz, 70 °C) of compound **10i**.
FIGURE S40 ^{13}C NMR spectrum (DMSO-d_6, 126 MHz, 70 °C) of compound **10i**.
FIGURE S41 IR spectrum (KBr) of compound **10i**.
FIGURE S42 HRMS spectrum (ESI) of compound **10i**.
FIGURE S43 HPLC chromatogram of compound **10i**.
FIGURE S44 ^{1}H NMR spectrum (DMSO-d_6, 400 MHz) of compound **10j**.
FIGURE S45 ^{13}C NMR spectrum (DMSO-d_6, 101 MHz) of compound **10j**.
FIGURE S46 HRMS spectrum (ESI) of compound **10j**.
FIGURE S47 HPLC chromatogram of compound **10j**.
FIGURE S48 ^{1}H NMR spectrum (DMSO-d_6, 500 MHz) of compound **10k**.
FIGURE S49 ^{13}C NMR spectrum (DMSO-d_6, 126 MHz) of compound **10k**.
FIGURE S50 IR spectrum (KBr) of compound **10k**.
FIGURE S51 HRMS spectrum (ESI) of compound **10k**.
FIGURE S52 HPLC chromatogram of compound **10k**.
FIGURE S53 ^{1}H NMR spectrum (DMSO-d_6, 500 MHz) of compound **10l**.
FIGURE S54 ^{13}C NMR spectrum (DMSO-d_6, 126 MHz) of compound **10l**.
FIGURE S55 IR spectrum (KBr) of compound **10l**.
FIGURE S56 HRMS spectrum (ESI) of compound **10l**.
FIGURE S57 ^{1}H NMR spectrum (DMSO-d_6, 500 MHz) of compound **11**.
FIGURE S58 ^{13}C NMR APT spectrum (DMSO-d_6, 126 MHz) of compound **11**.
FIGURE S59 IR spectrum (KBr) of compound **11**.

WILEY-VCH

FIGURE S60 HRMS spectrum (ESI) of compound **11**.
FIGURE S61 ^{1}H NMR spectrum (DMSO-d_6, 500 MHz) of compound **12**.
FIGURE S62 ^{13}C NMR spectrum (DMSO-d_6, 126 MHz) of compound **12**.
FIGURE S63 IR spectrum (KBr) of compound **12**.
FIGURE S64 HRMS spectrum (ESI) of compound **12**.

WILEY-VCH

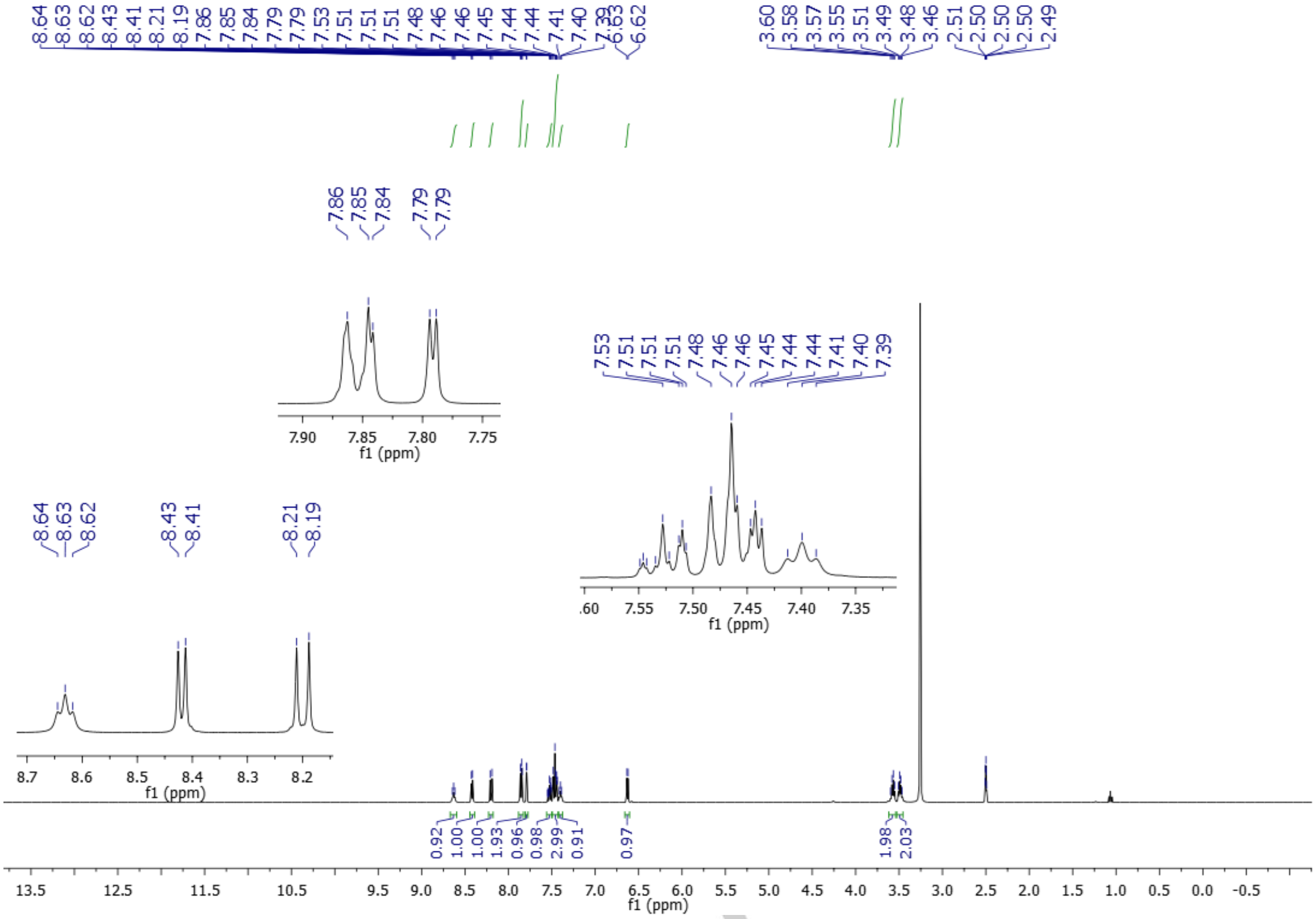

FIGURE S1 ^{1}H NMR spectrum (DMSO-d_6, 400 MHz) of compound **10a**.

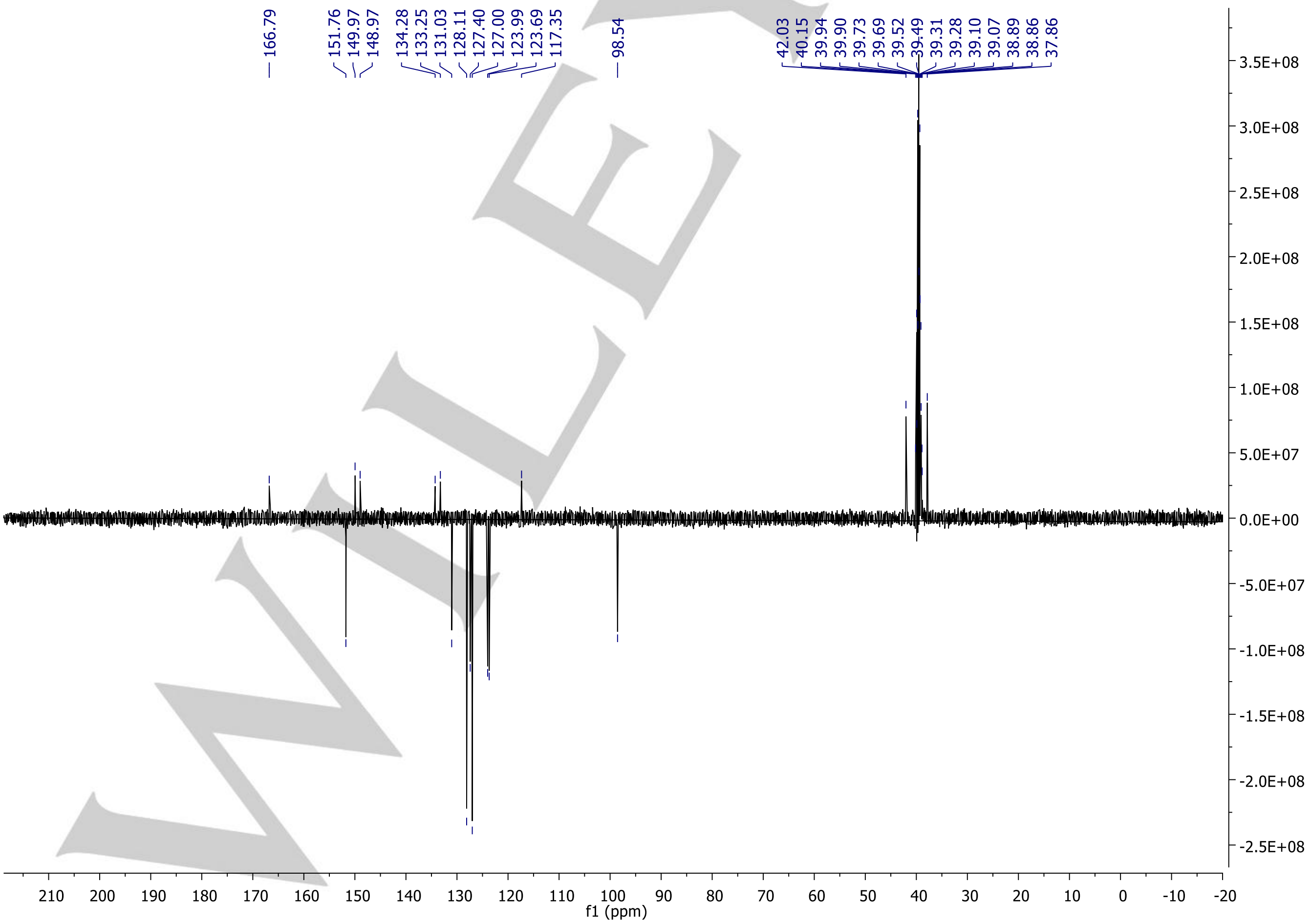

FIGURE S2 ^{13}C NMR APT spectrum (DMSO-d_6, 101 MHz) of compound **10a**.

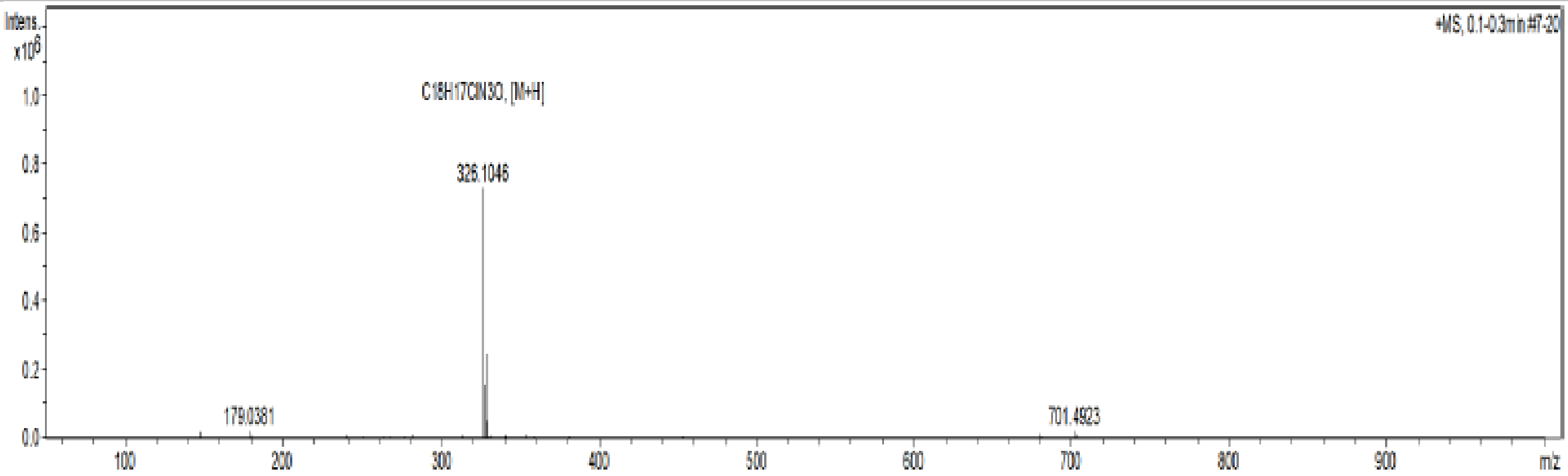

Meas. m/z	#	Ion Formula	m/z	err [ppm]	mSigma	# mSigma	Score	rdb	e¯ Conf	N-Rule
326.1046	1	C18H17ClN3O	326.1055	2.6	2.3	1	100.00	11.5	even	ok

FIGURE S3 HRMS spectrum (ESI) of compound **10a**.

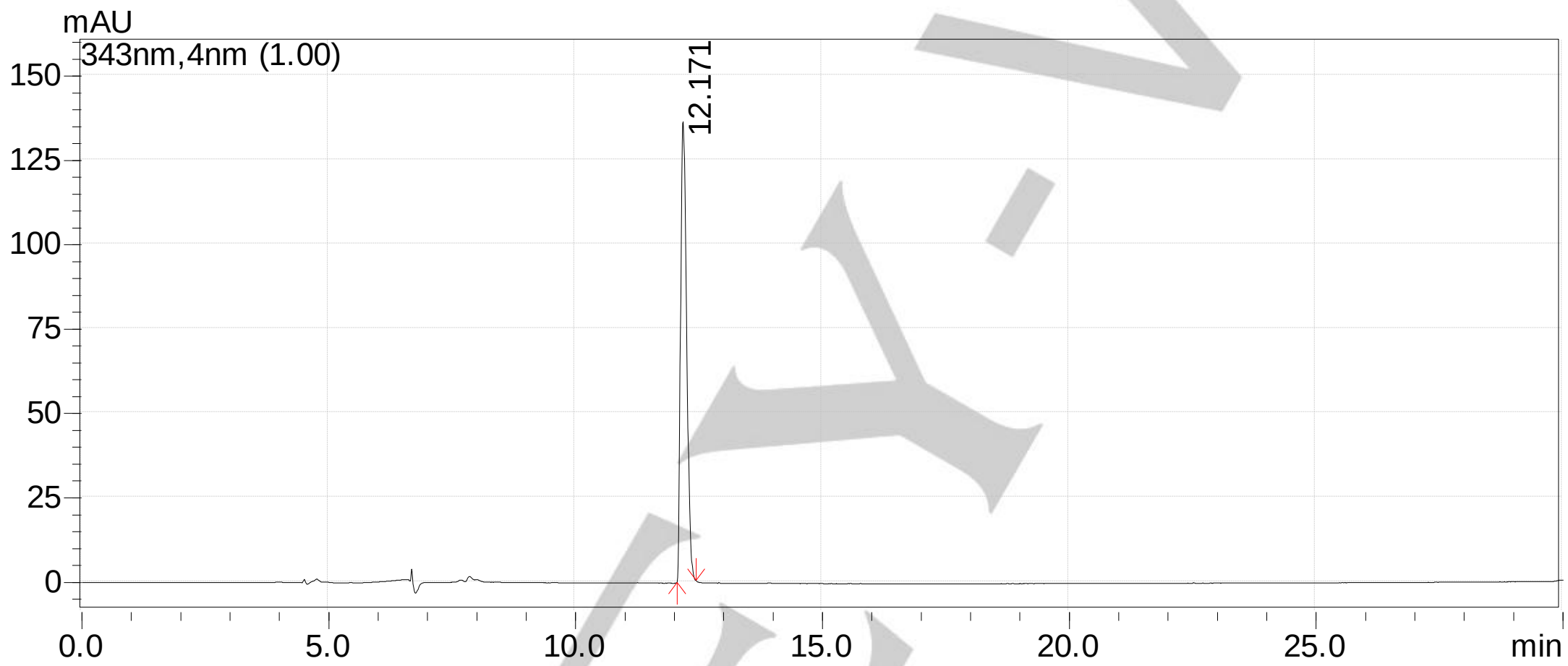

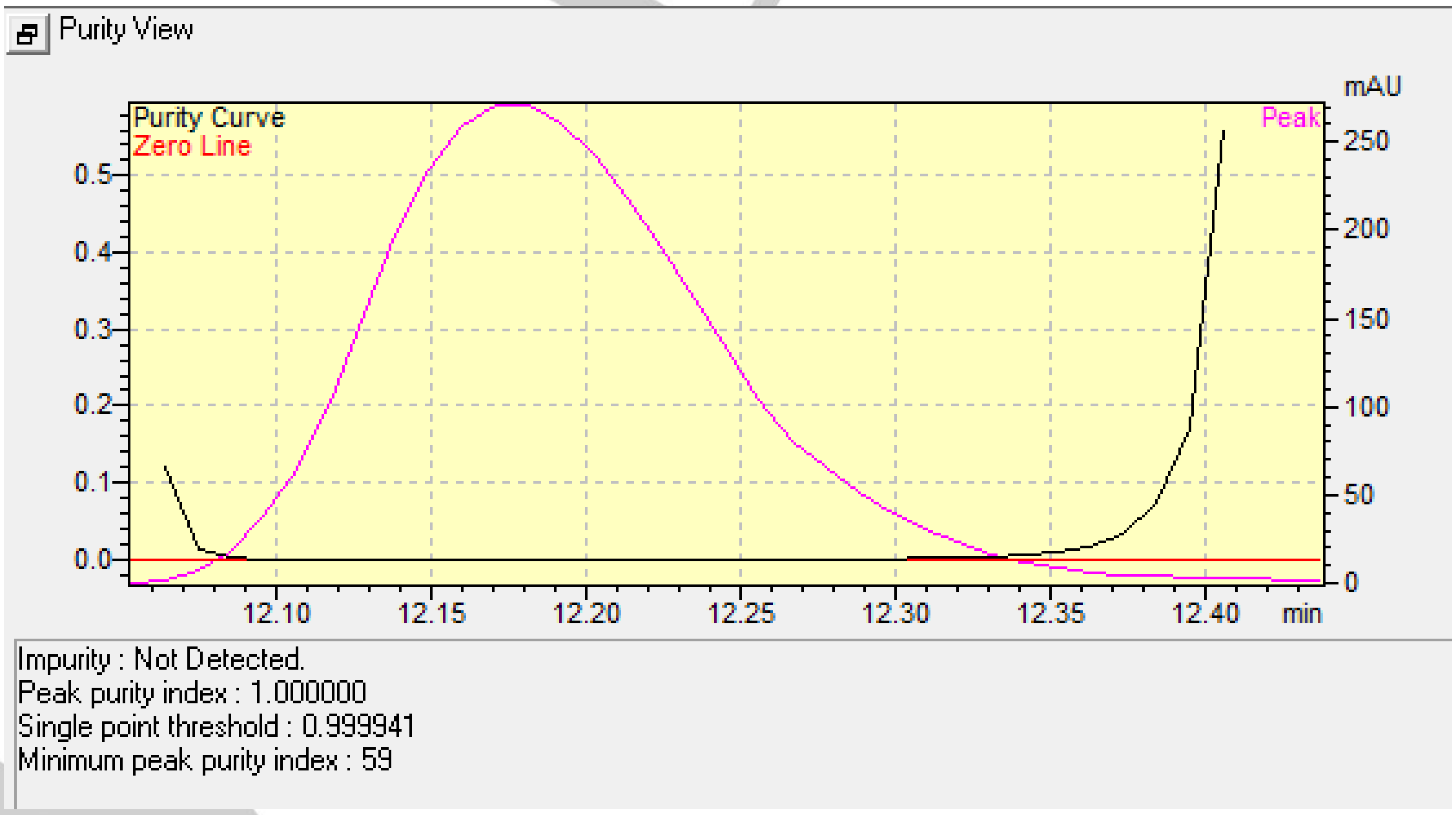

FIGURE S4 HPLC chromatogram of compound **10a**.

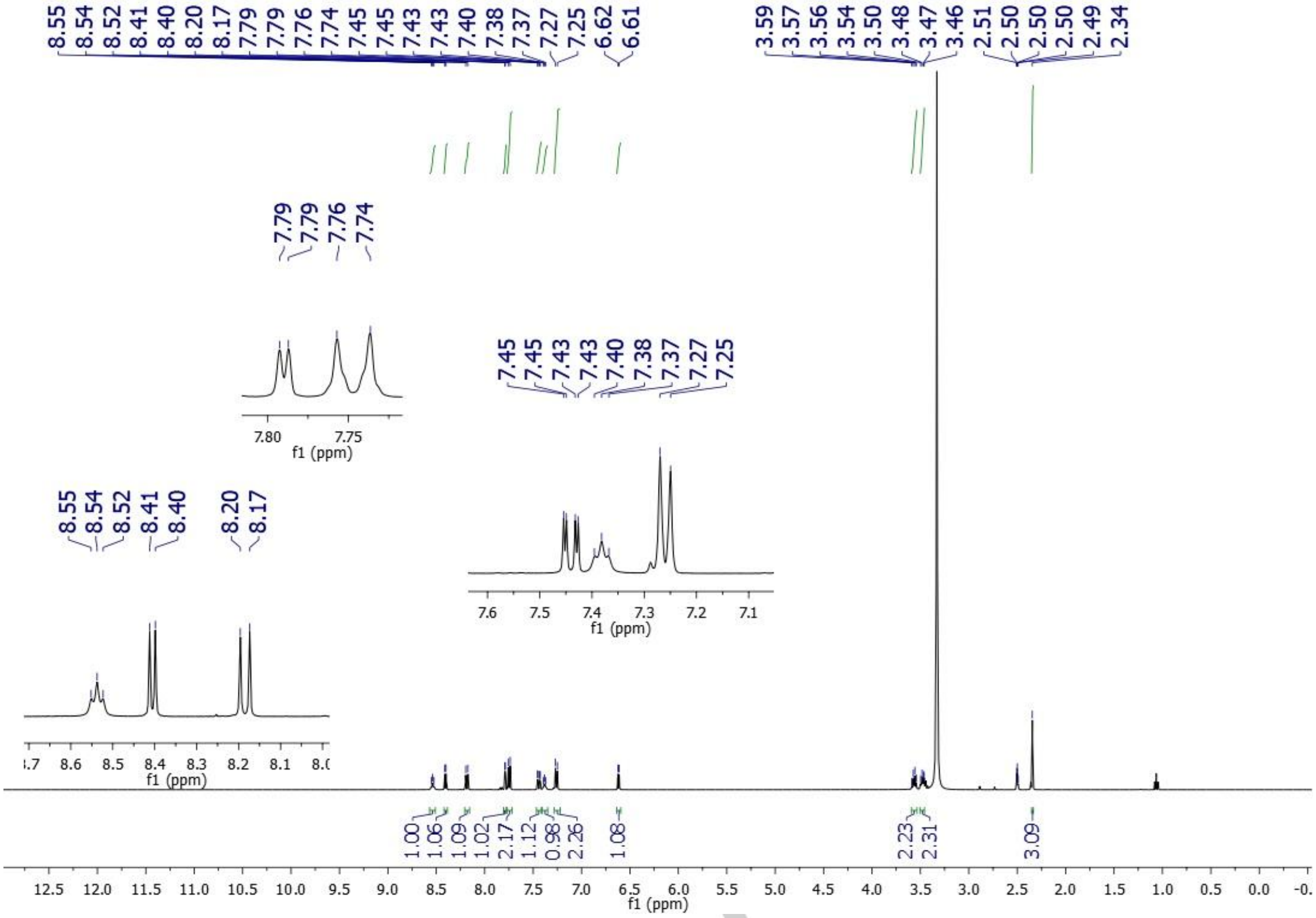

FIGURE S5 ^{1}H NMR spectrum (DMSO-d_6, 400 MHz) of compound **10b**.

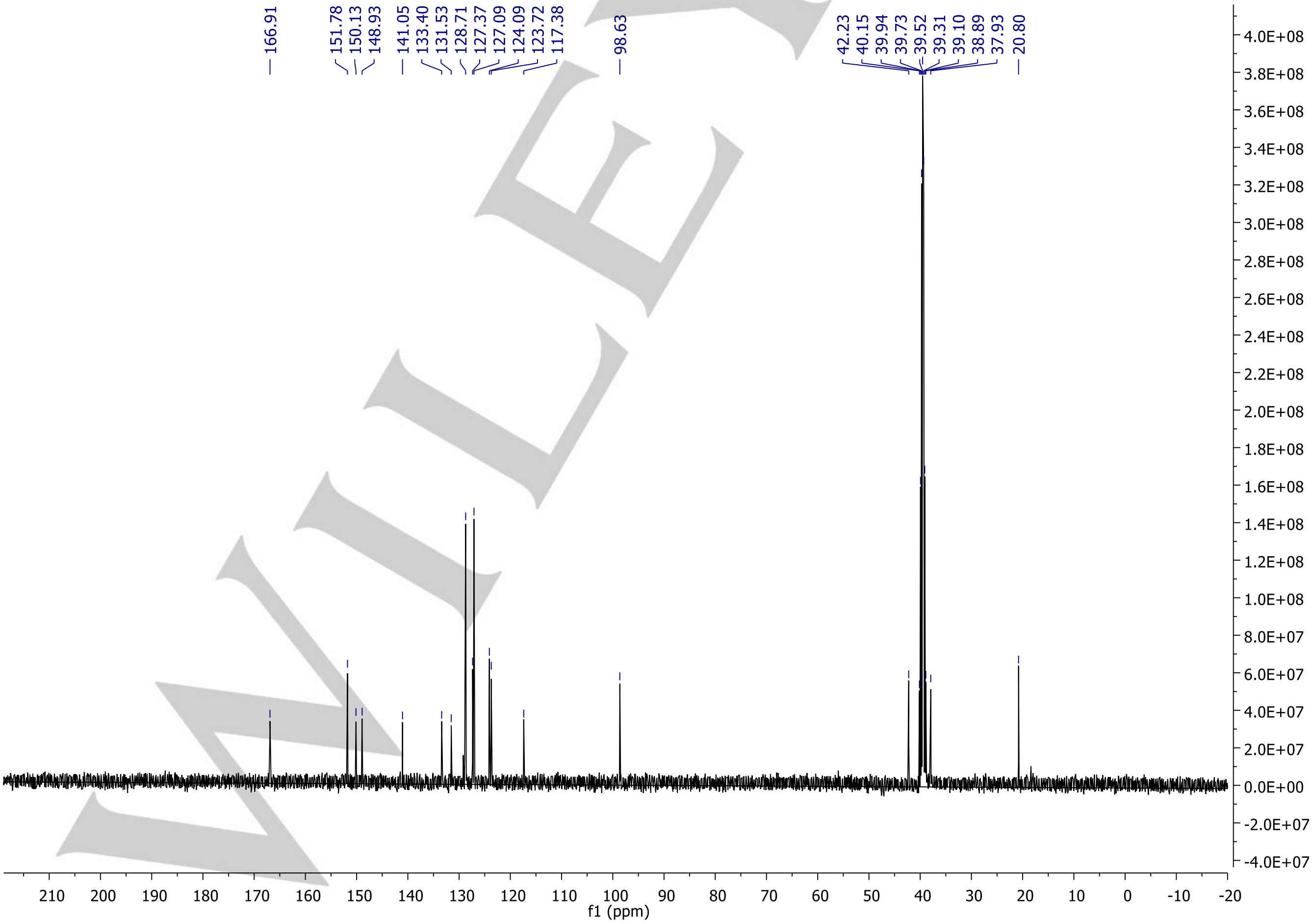

FIGURE S6 ^{13}C NMR spectrum (DMSO-d_6, 101 MHz) of compound **10b**.

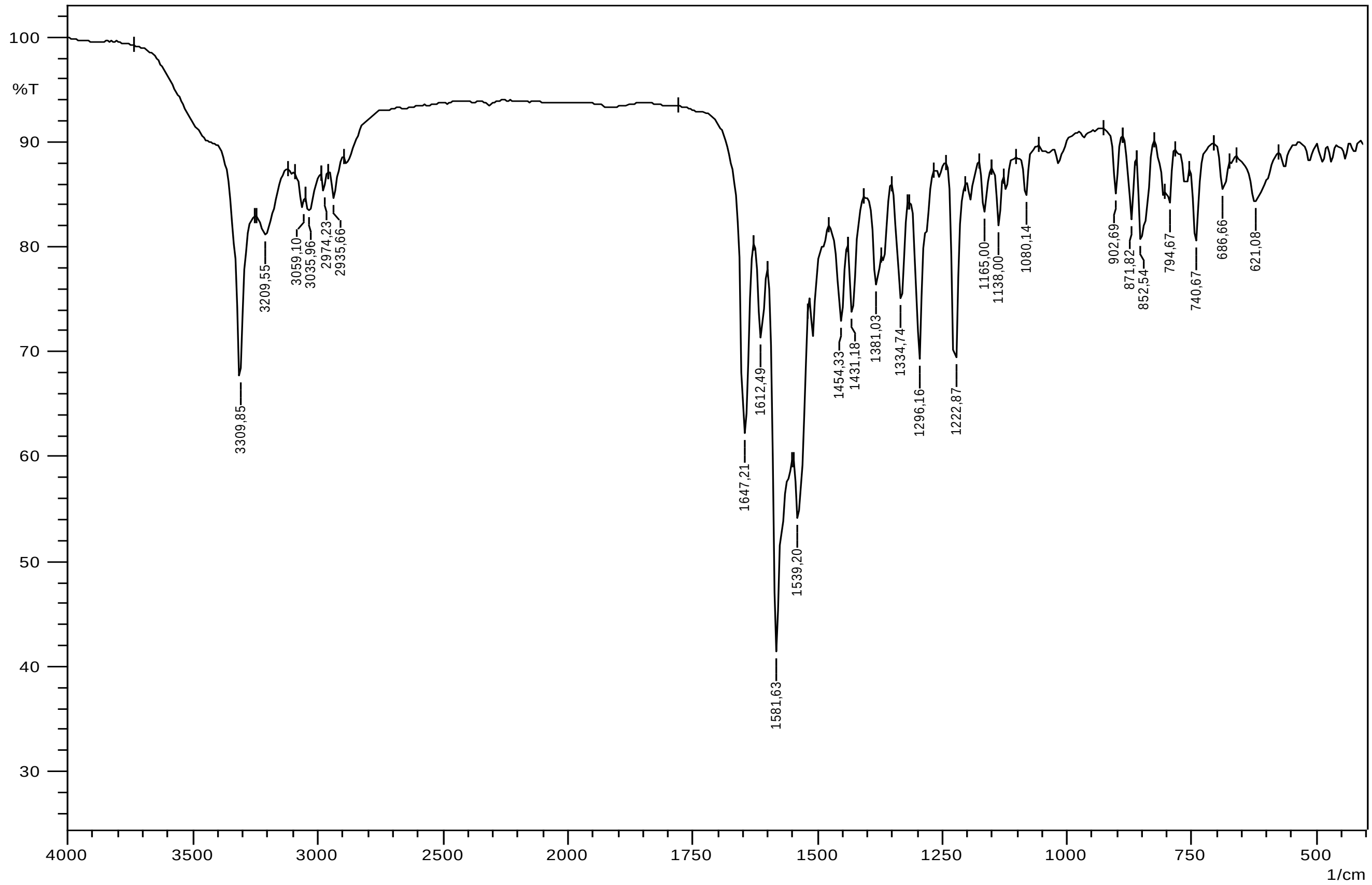

FIGURE S7 IR spectrum (KBr) of compound **10b**.

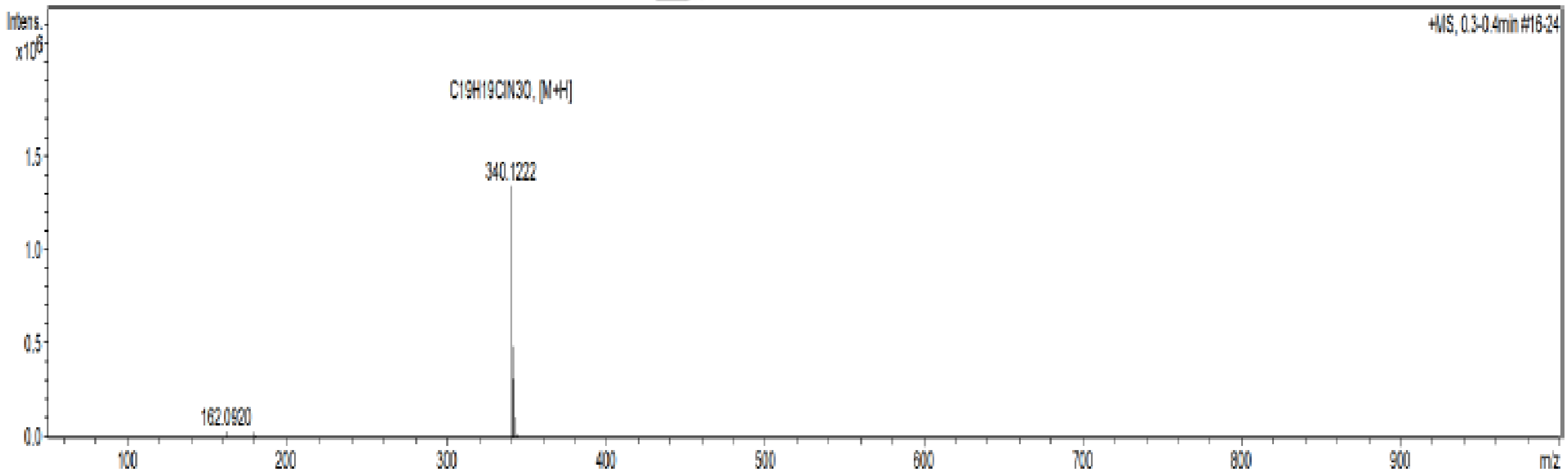

Meas. m/z	#	Ion Formula	m/z	err [ppm]	mSigma	# mSigma	Score	rdb	e⁻ Conf	N-Rule
340.1222	1	C19H19ClN3O	340.1211	-3.3	11.0	1	100.00	11.5	even	ok

FIGURE S8 HRMS spectrum (ESI) of compound **10b**.

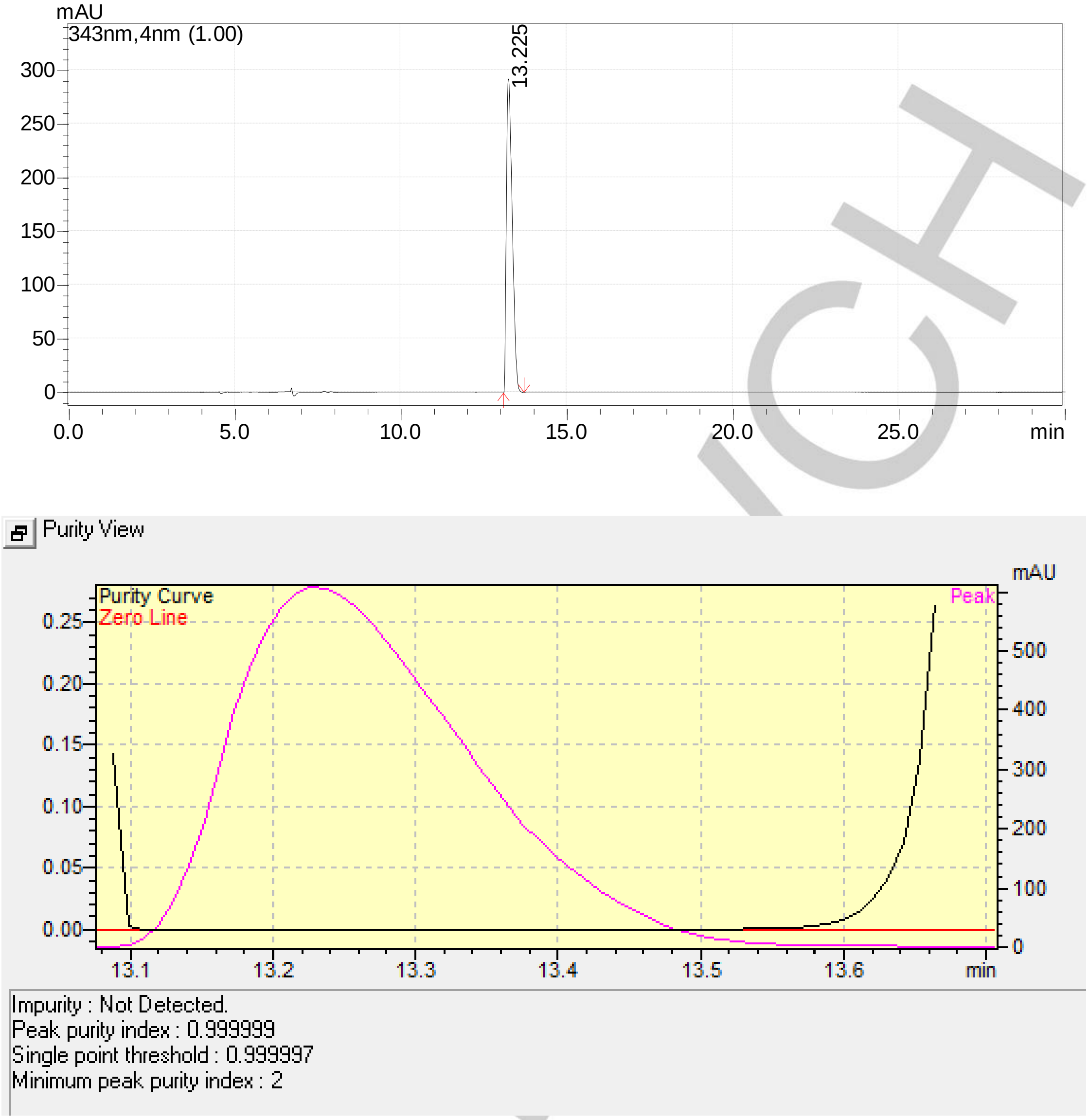

FIGURE S9 HPLC chromatrogram of compound **10b**.

WILEY-VCH

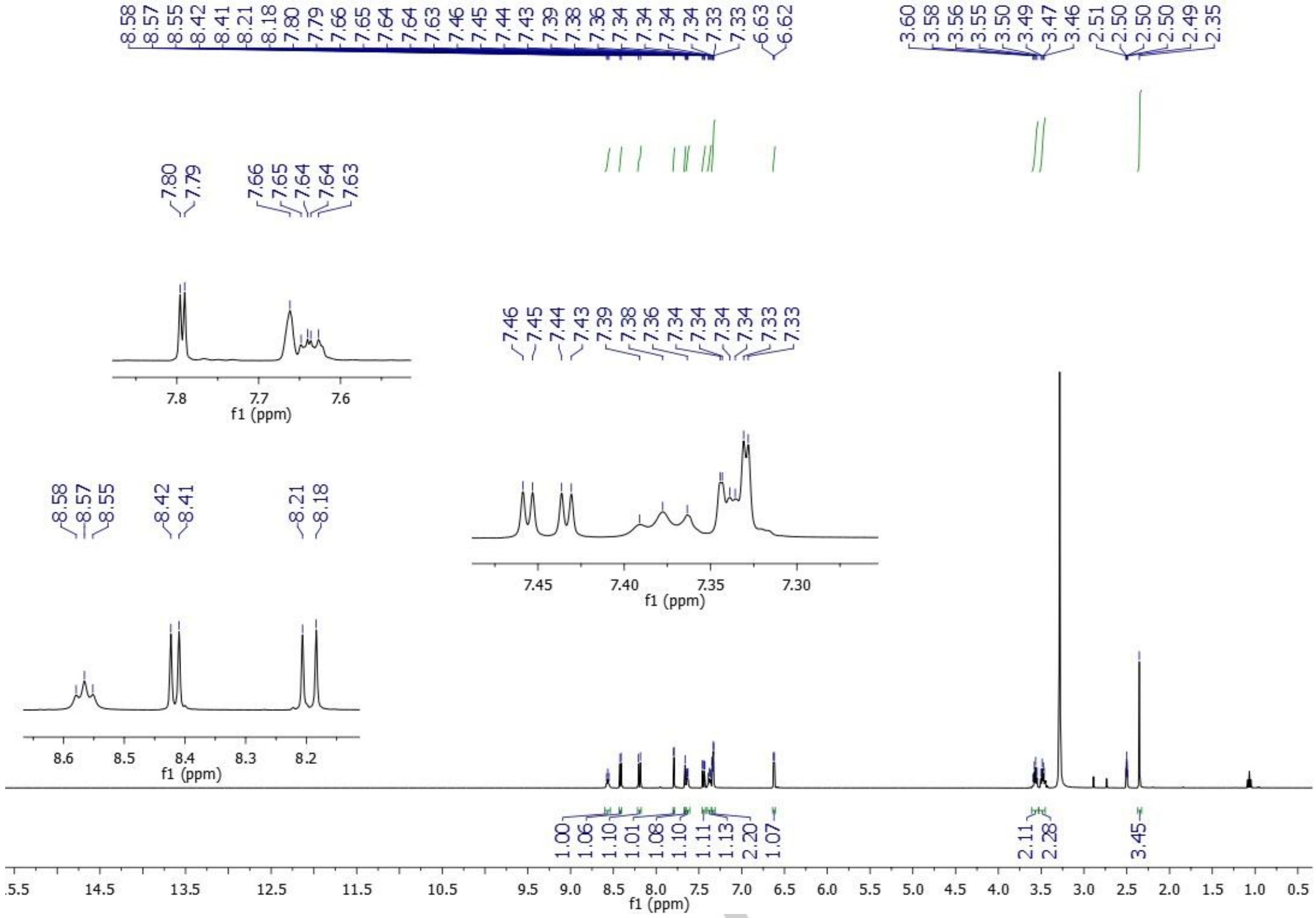

FIGURE S10 ^{1}H NMR spectrum (DMSO-d_6, 400 MHz) of compound **10c**.

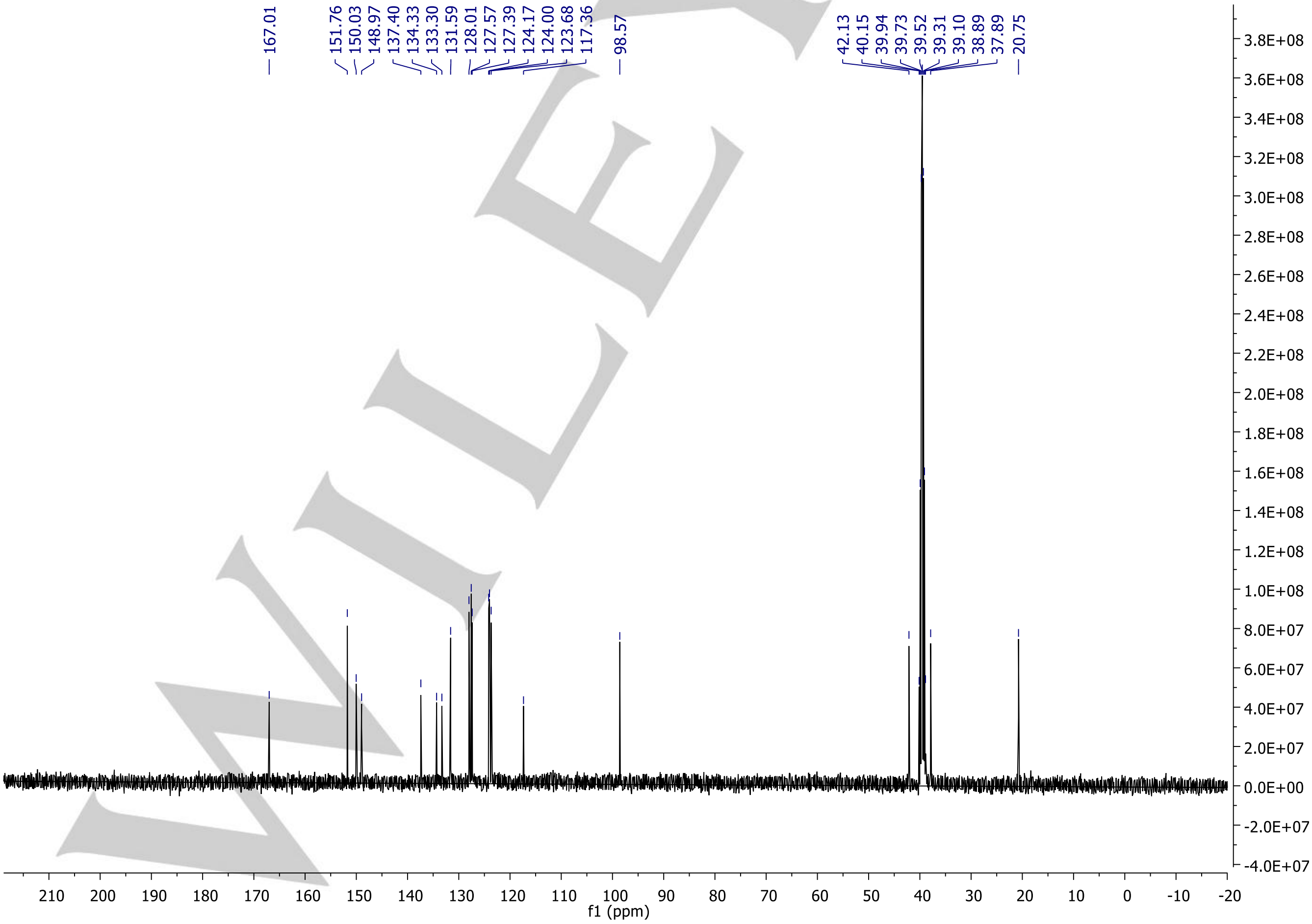

FIGURE S11 ^{13}C NMR spectrum (DMSO-d_6, 101 MHz) of compound **10c**.

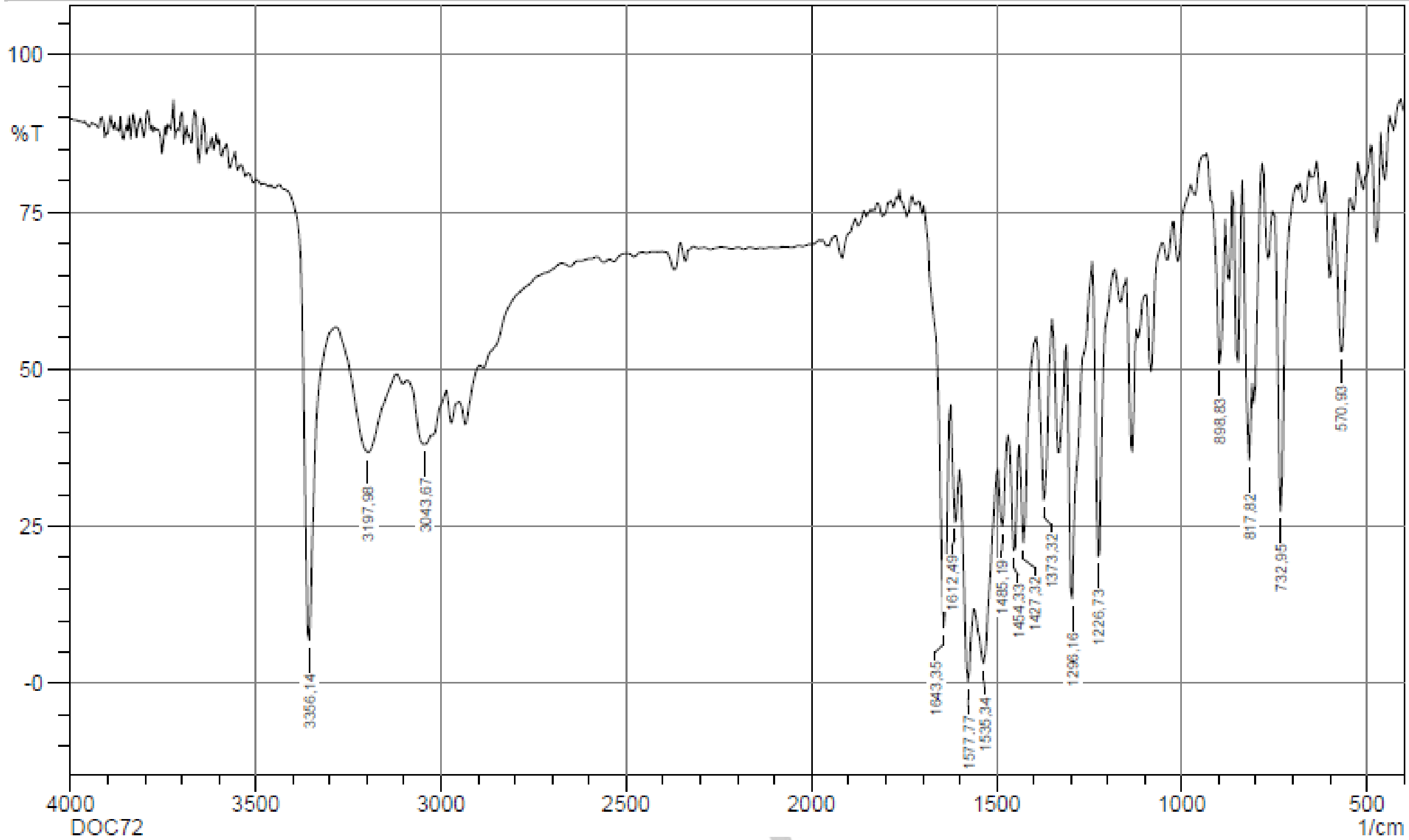

FIGURE S12 IR spectrum (KBr) of compound **10c**.

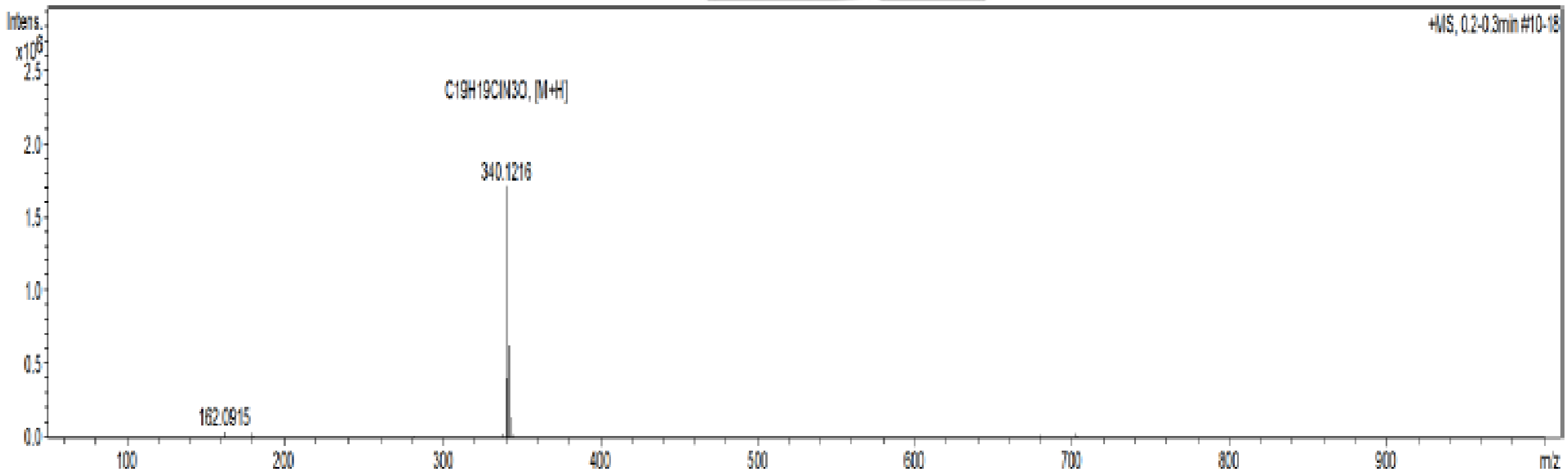

Meas. m/z	#	Ion Formula	m/z	err [ppm]	mSigma	# mSigma	Score	rdb	e⁻ Conf	N-Rule
340.1216	1	C19H19ClN3O	340.1211	-1.3	14.9	1	100.00	11.5	even	ok

FIGURE S13 HRMS spectrum (ESI) of compound **10c**.

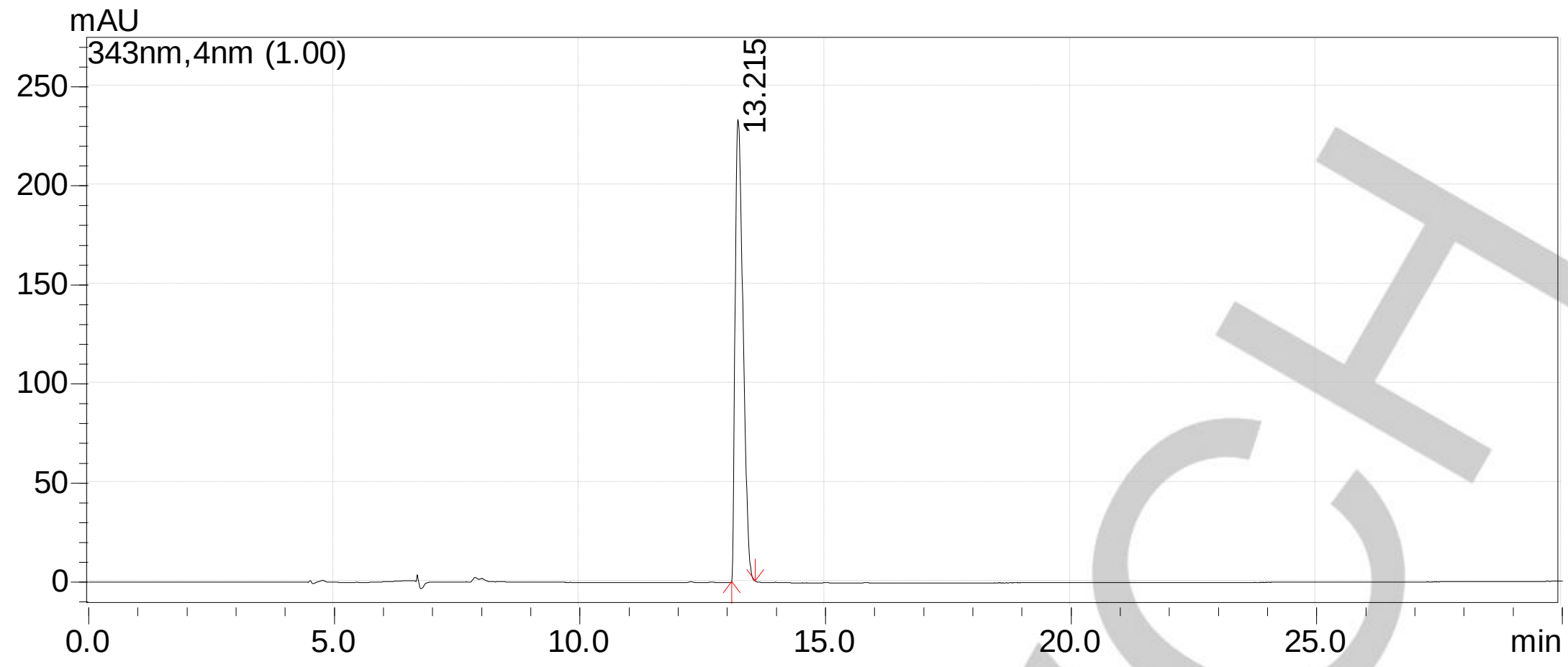

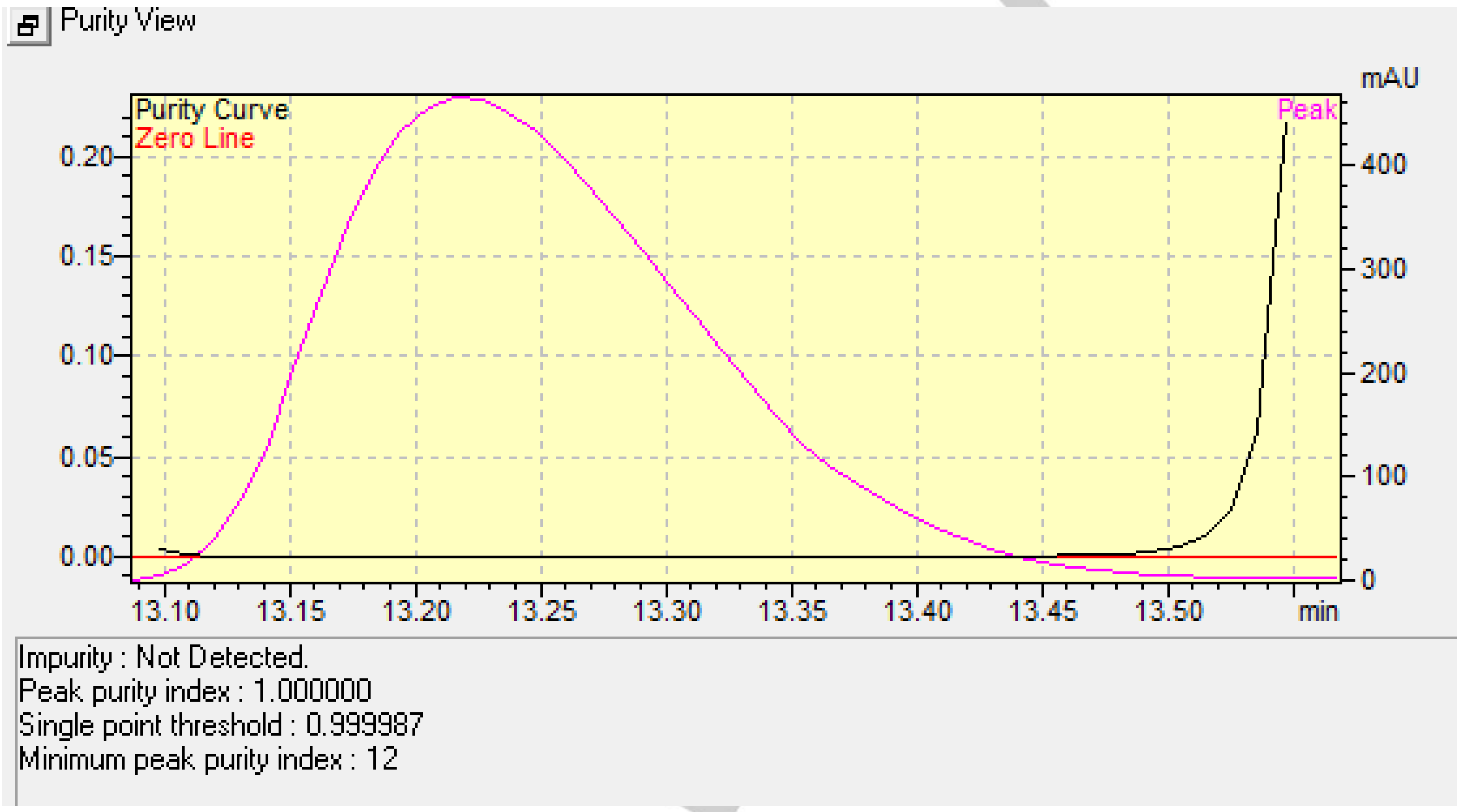

FIGURE S14 HPLC chromatrogram of compound **10c**.

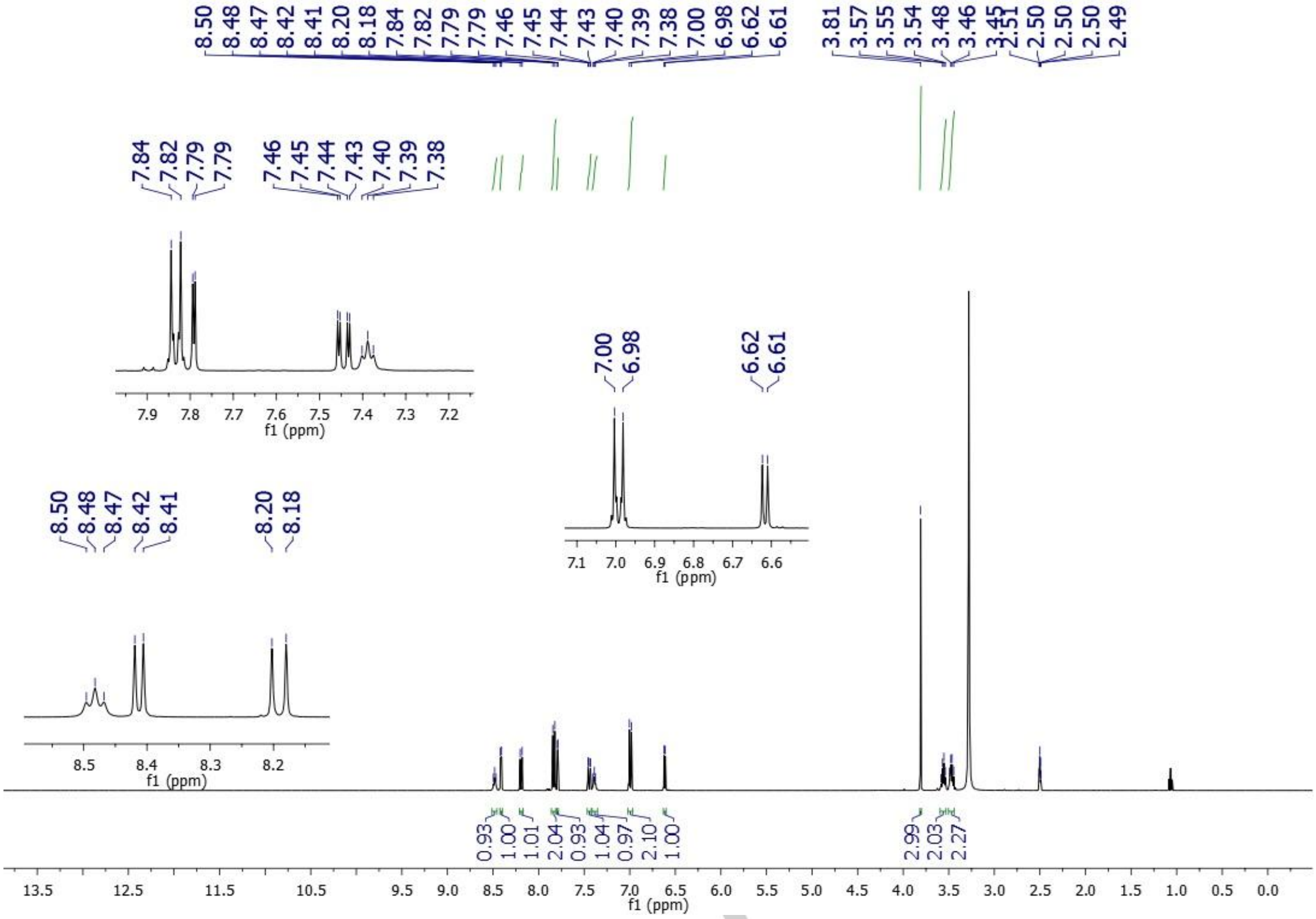

FIGURE S15 ^{1}H NMR spectrum (DMSO-d_6, 400 MHz) of compound **10d**.

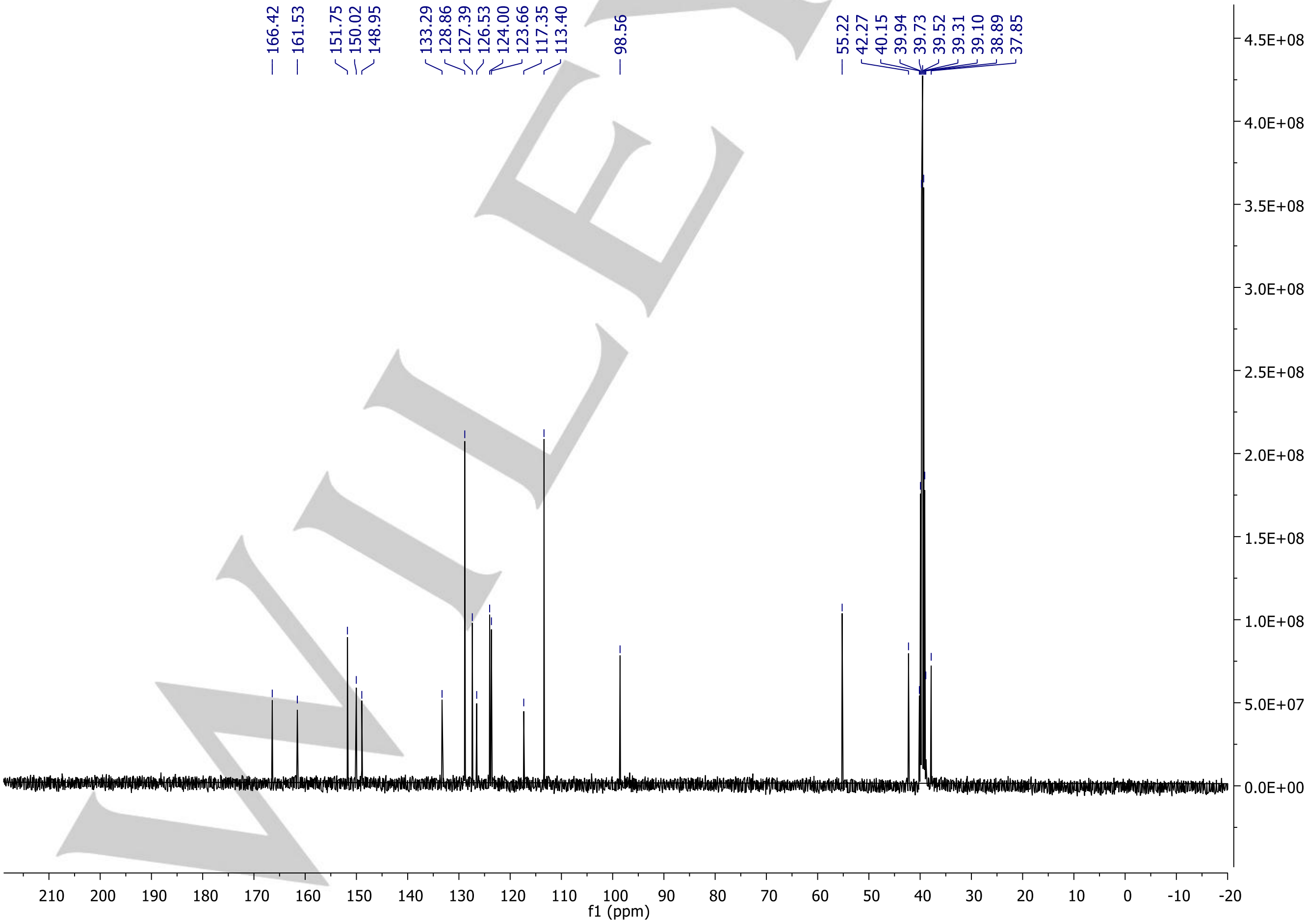

FIGURE S16 ^{13}C NMR spectrum (DMSO-d_6, 101 MHz) of compound **10d**.

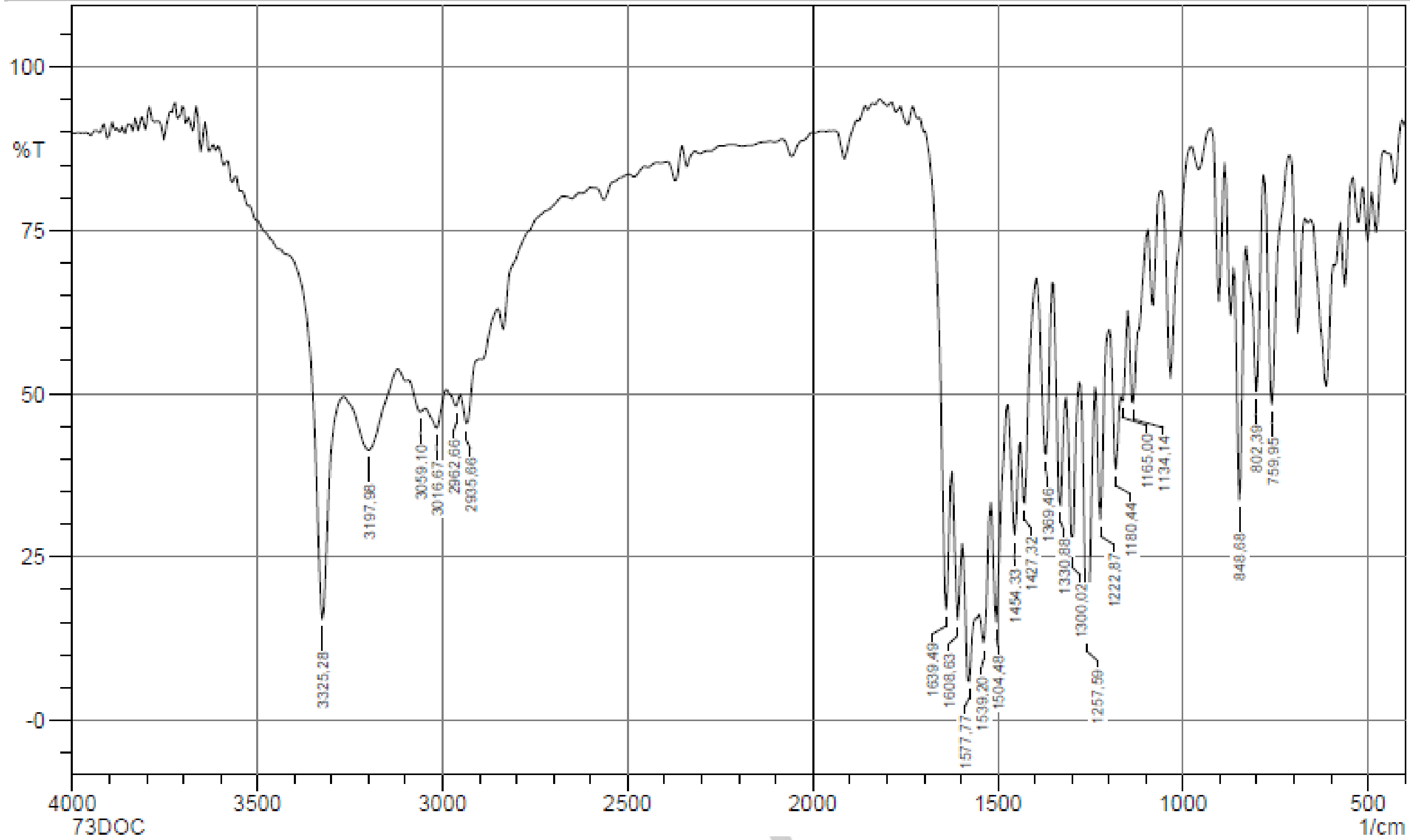

FIGURE S17 IR spectrum (KBr) of compound **10d**.

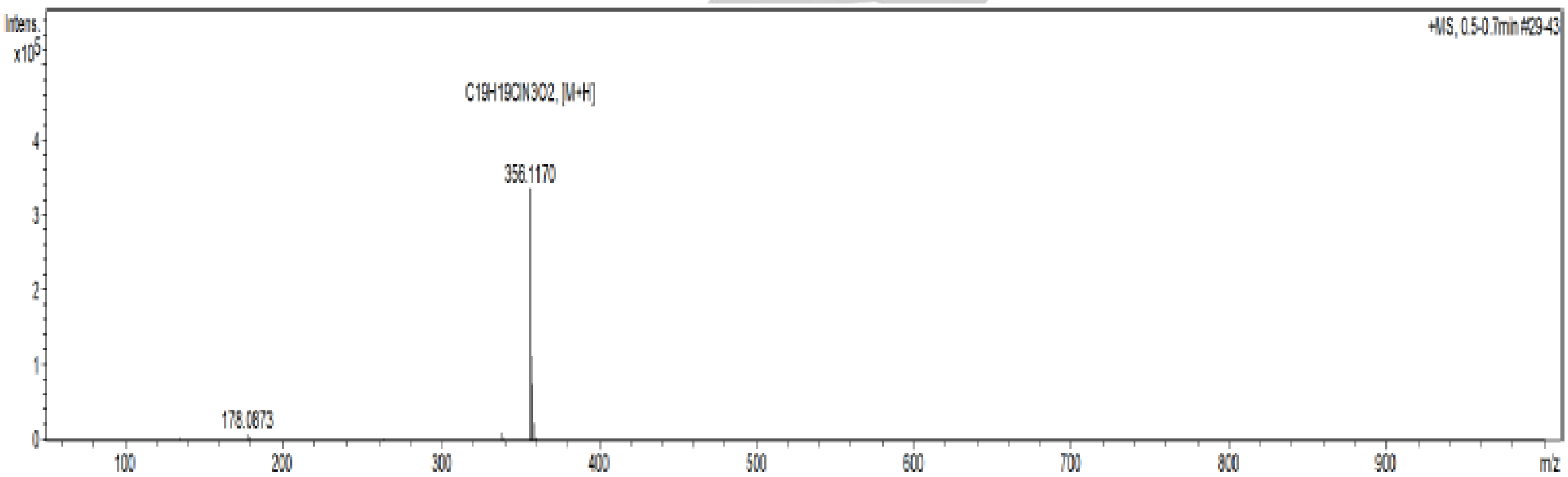

Meas. m/z	#	Ion Formula	m/z	err [ppm]	mSigma	# mSigma	Score	rdb	e¯ Conf	N-Rule
356.1170	1	C19H19ClN3O2	356.1160	-2.7	4.4	1	100.00	11.5	even	ok

FIGURE S18 HRMS spectrum (ESI) of compound **10d**.

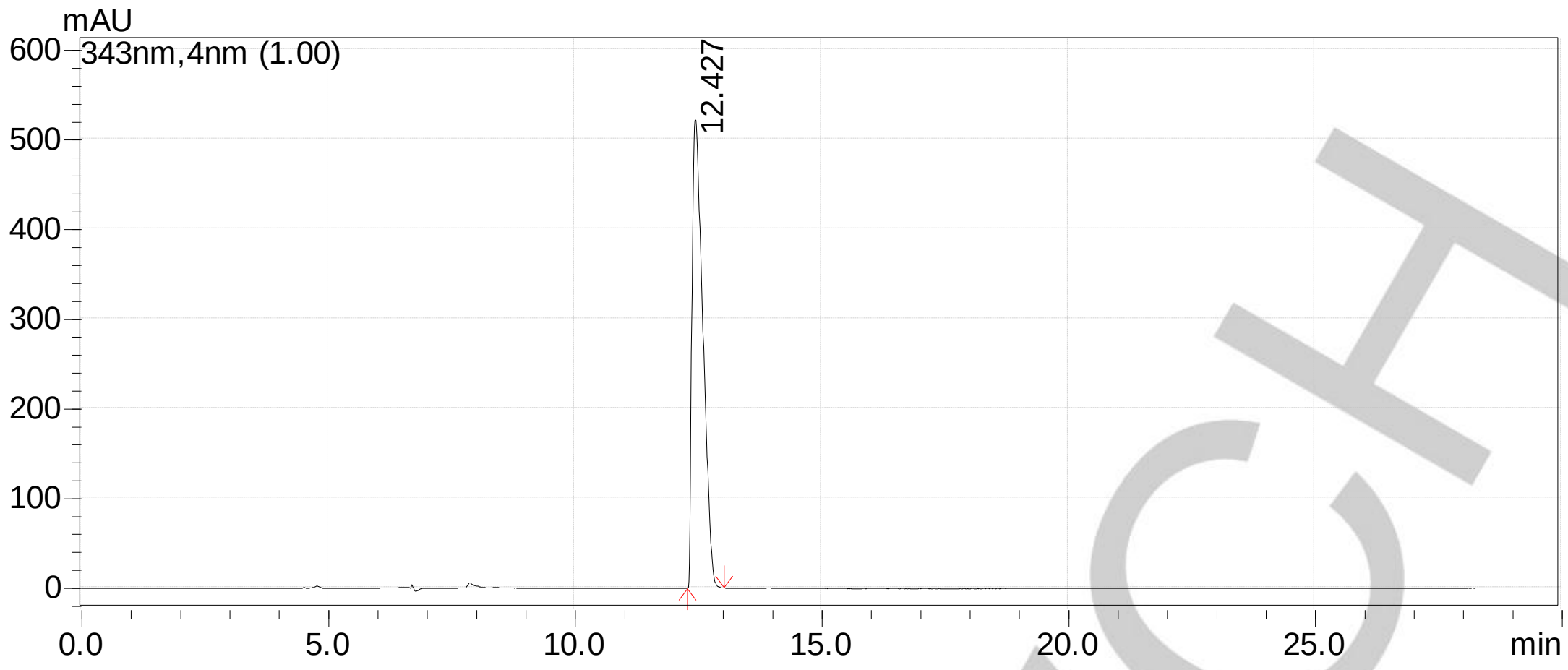

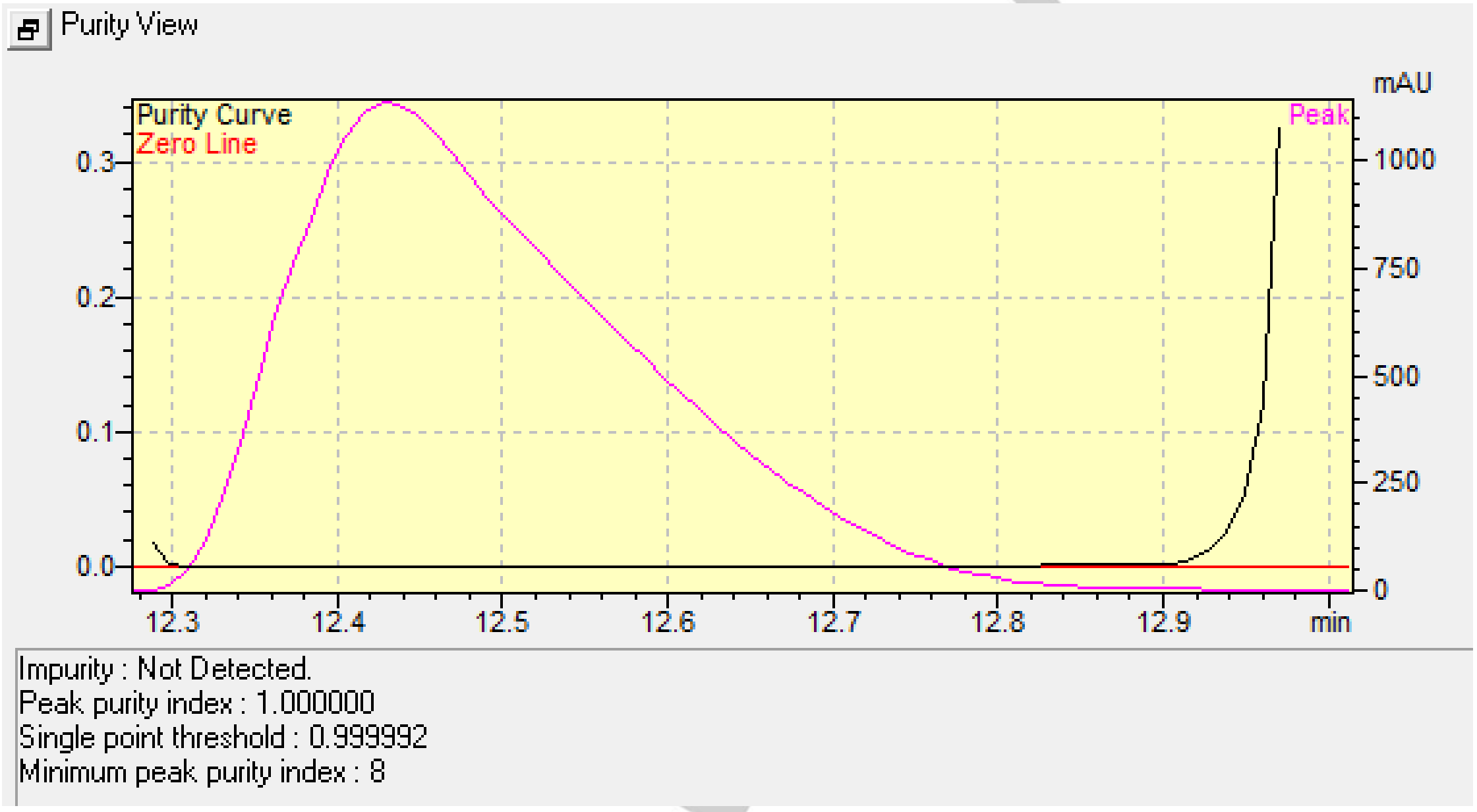

FIGURE S19 HPLC chromatrogram of compound **10d**.

WILEY-VCH

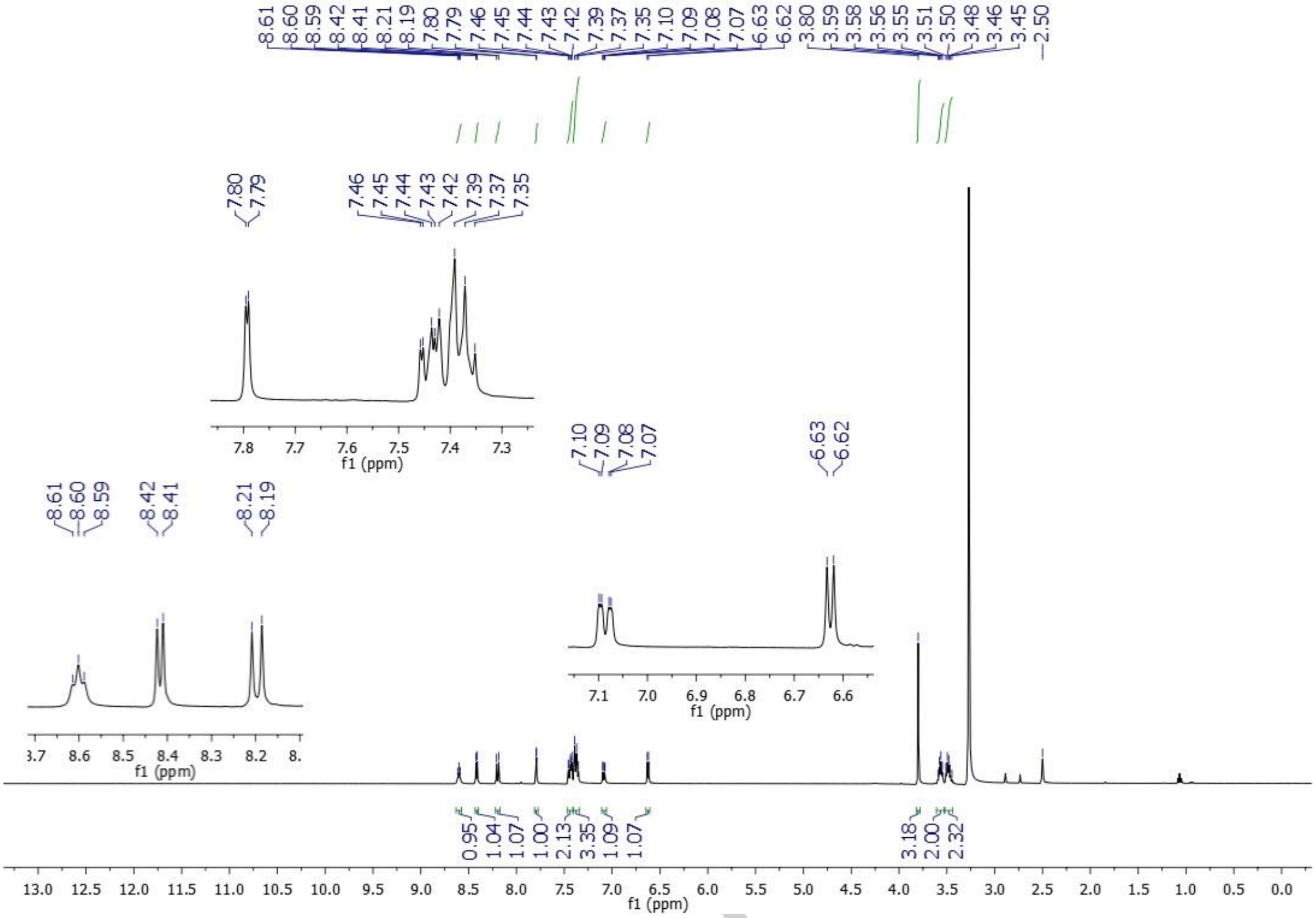

FIGURE S20 ^{1}H NMR spectrum (DMSO-d_6, 400 MHz) of compound **10e**.

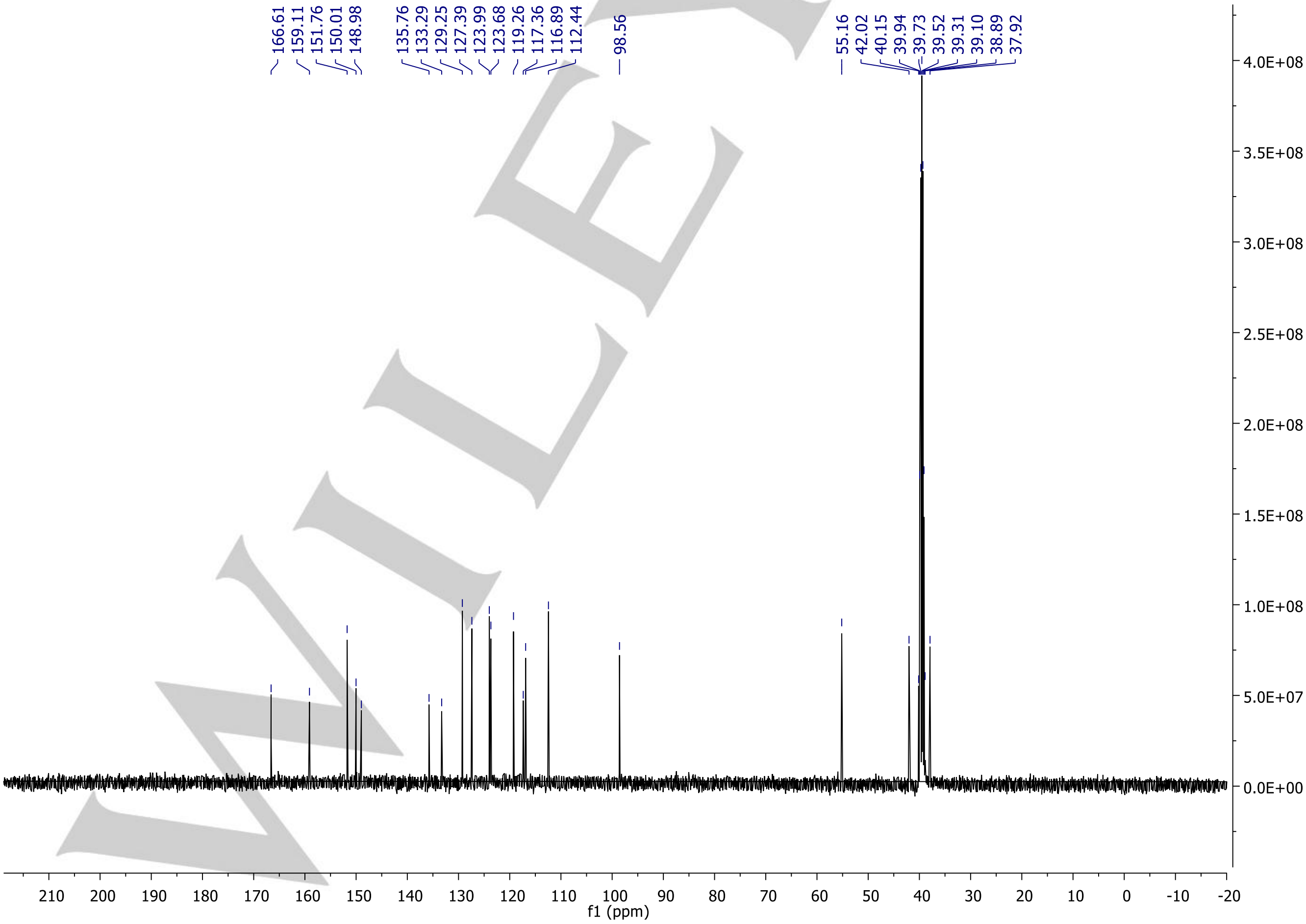

FIGURE S21 ^{13}C NMR spectrum (DMSO-d_6, 101 MHz) of compound **10e**.

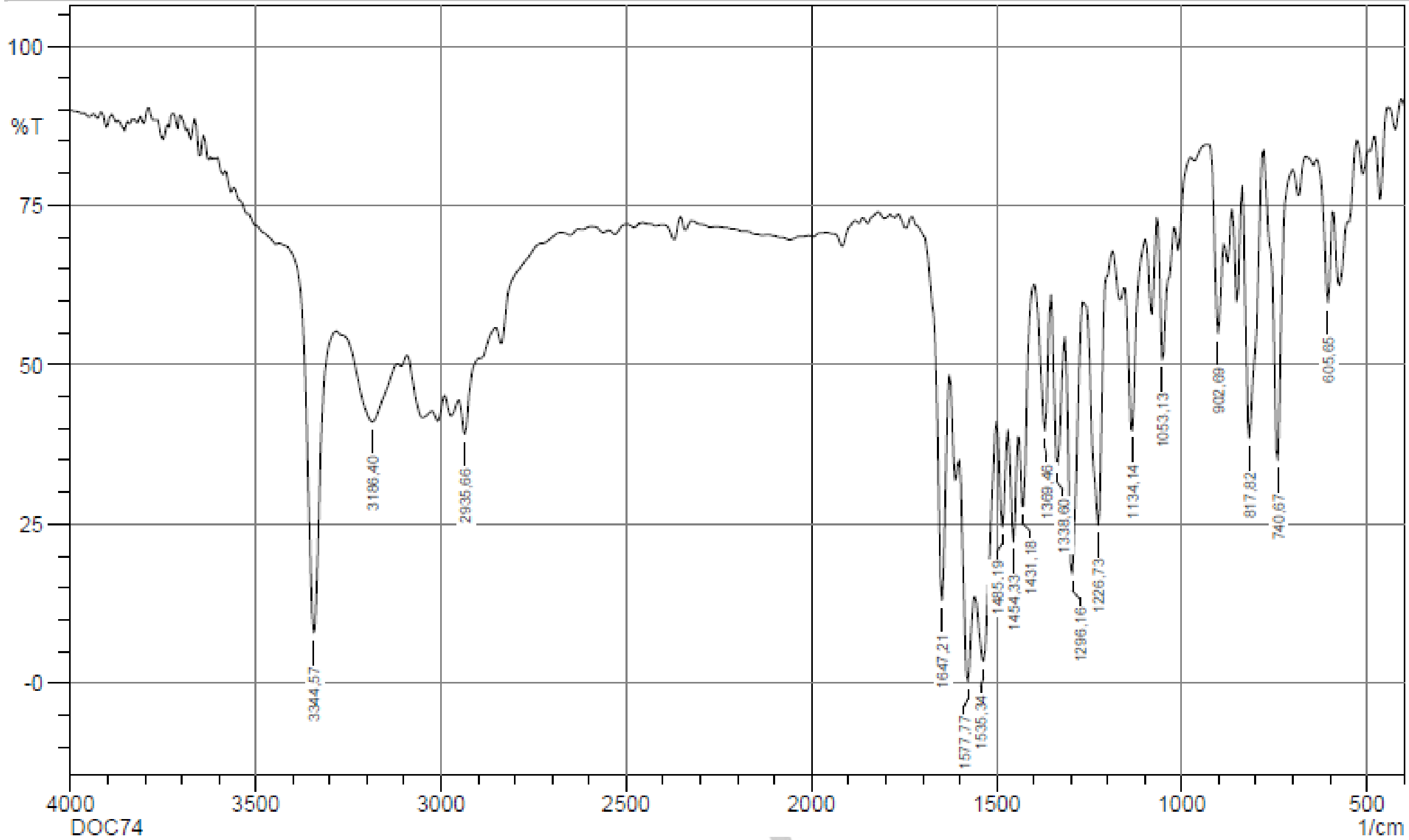

FIGURE S22 IR spectrum (KBr) of compound **10e**.

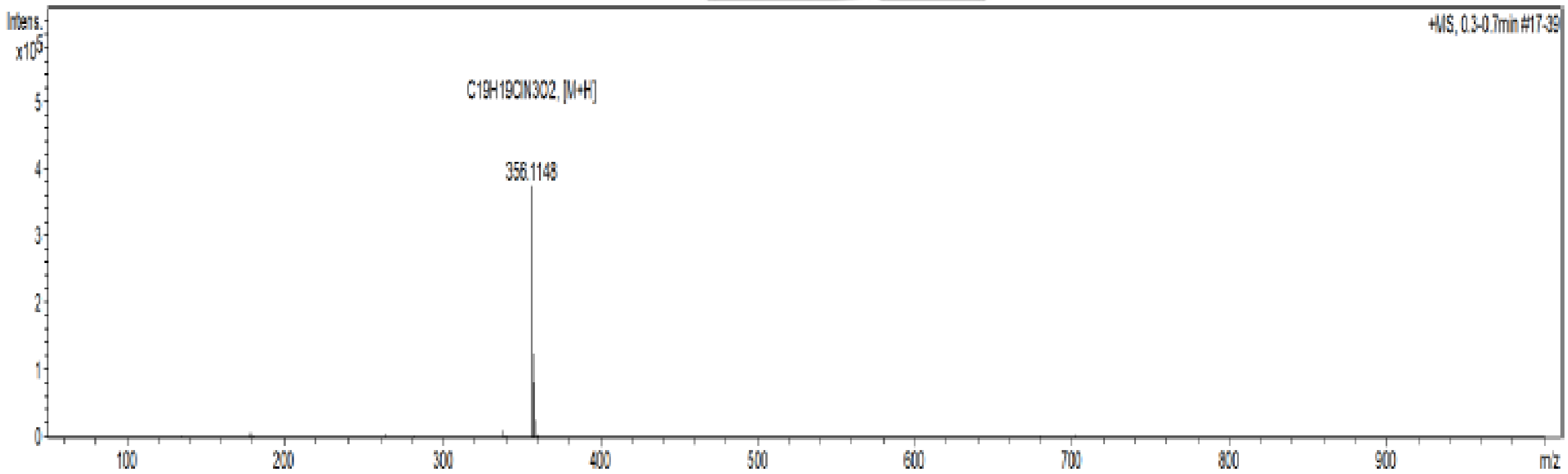

Meas. m/z	#	Ion Formula	m/z	err [ppm]	mSigma	# mSigma	Score	rdb	e¯ Conf	N-Rule
356.1148	1	C19H19ClN3O2	356.1160	3.5	4.7	1	100.00	11.5	even	ok

FIGURE S23 HRMS spectrum (ESI) of compound **10e**.

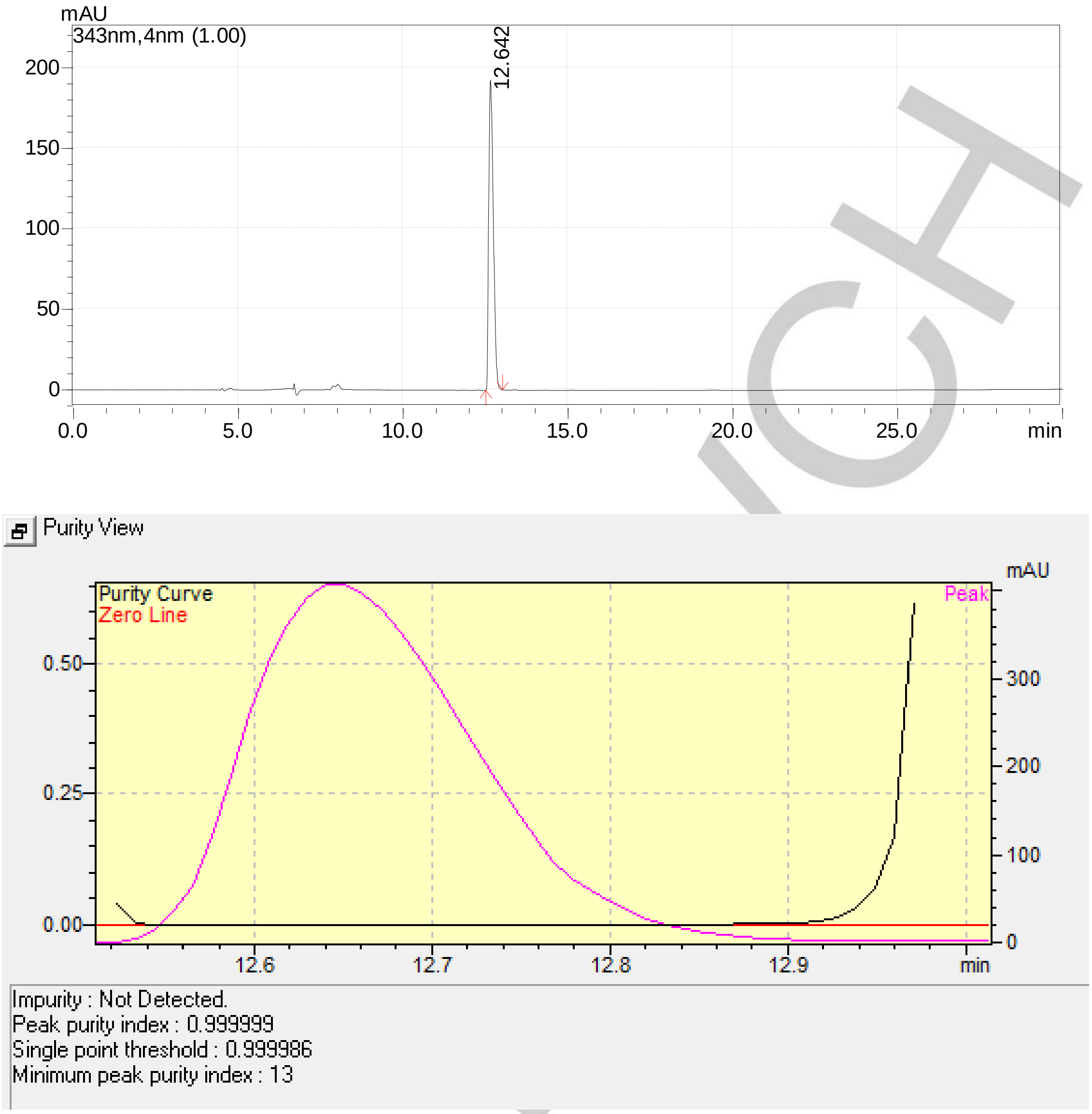

FIGURE S24 HPLC chromatrogram of compound **10e**.

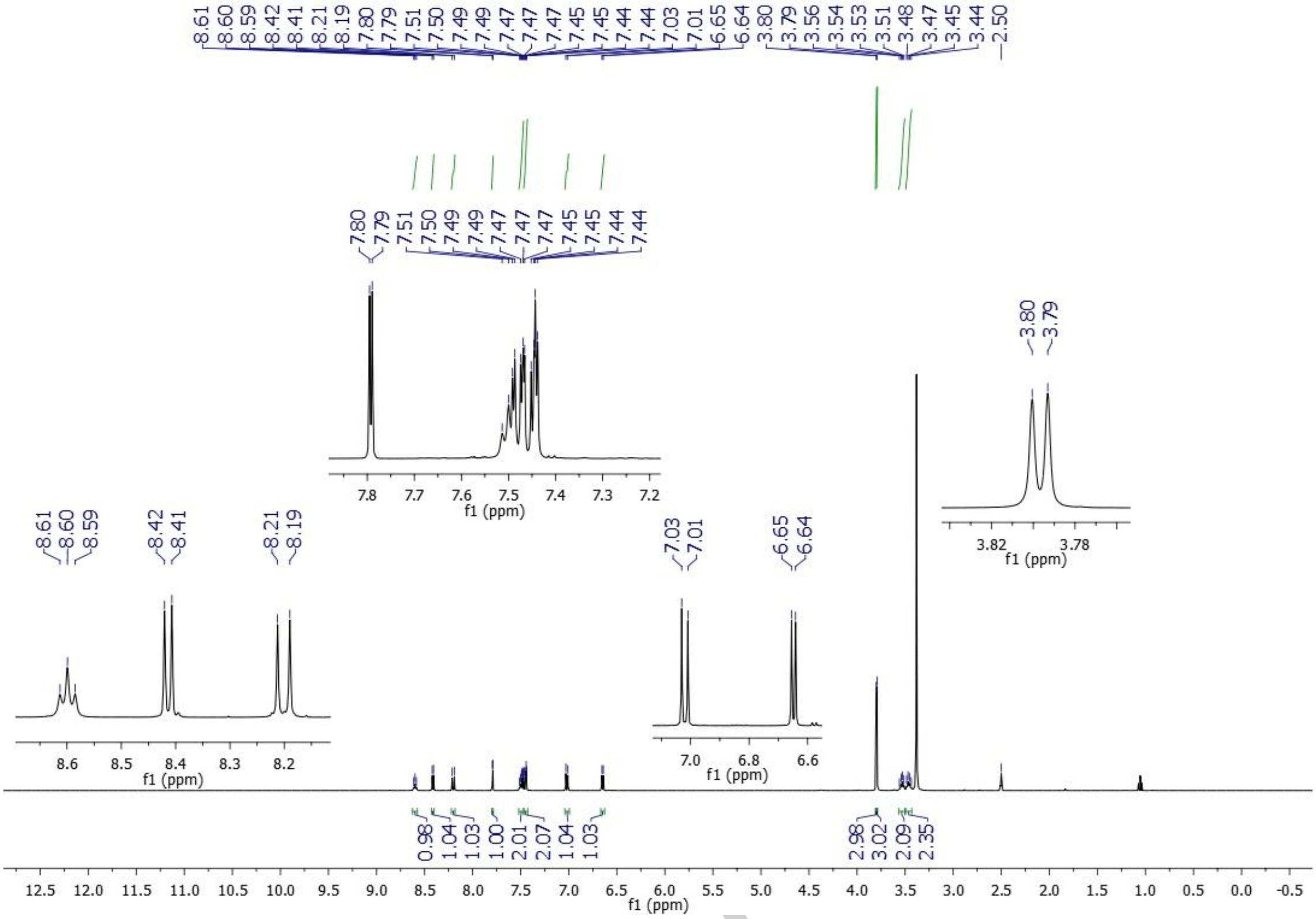

FIGURE S25 ^{1}H NMR spectrum (DMSO-d_6, 400 MHz) of compound **10f**.

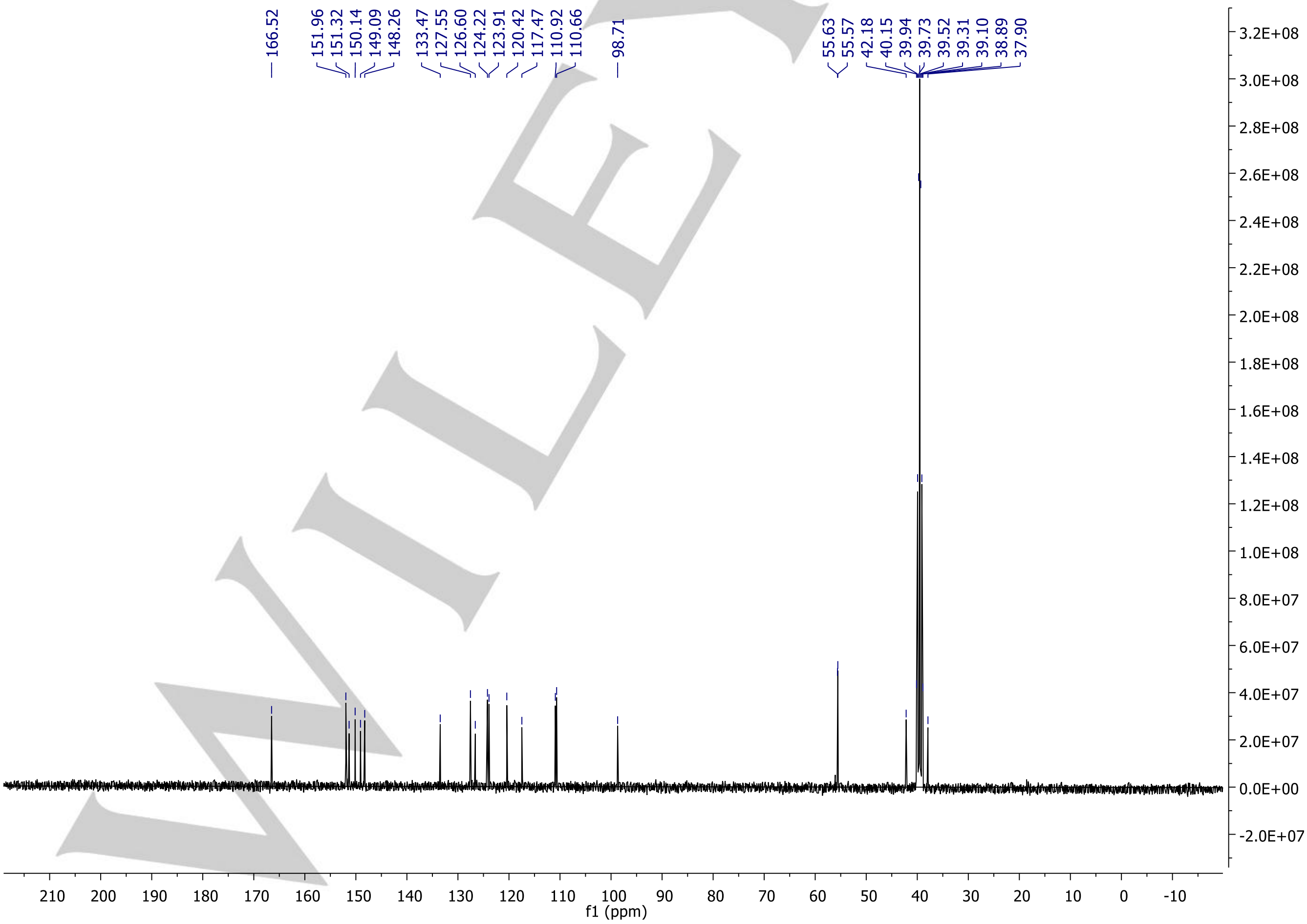

FIGURE S26 ^{13}C NMR spectrum (DMSO-d_6, 101 MHz) of compound **10f**.

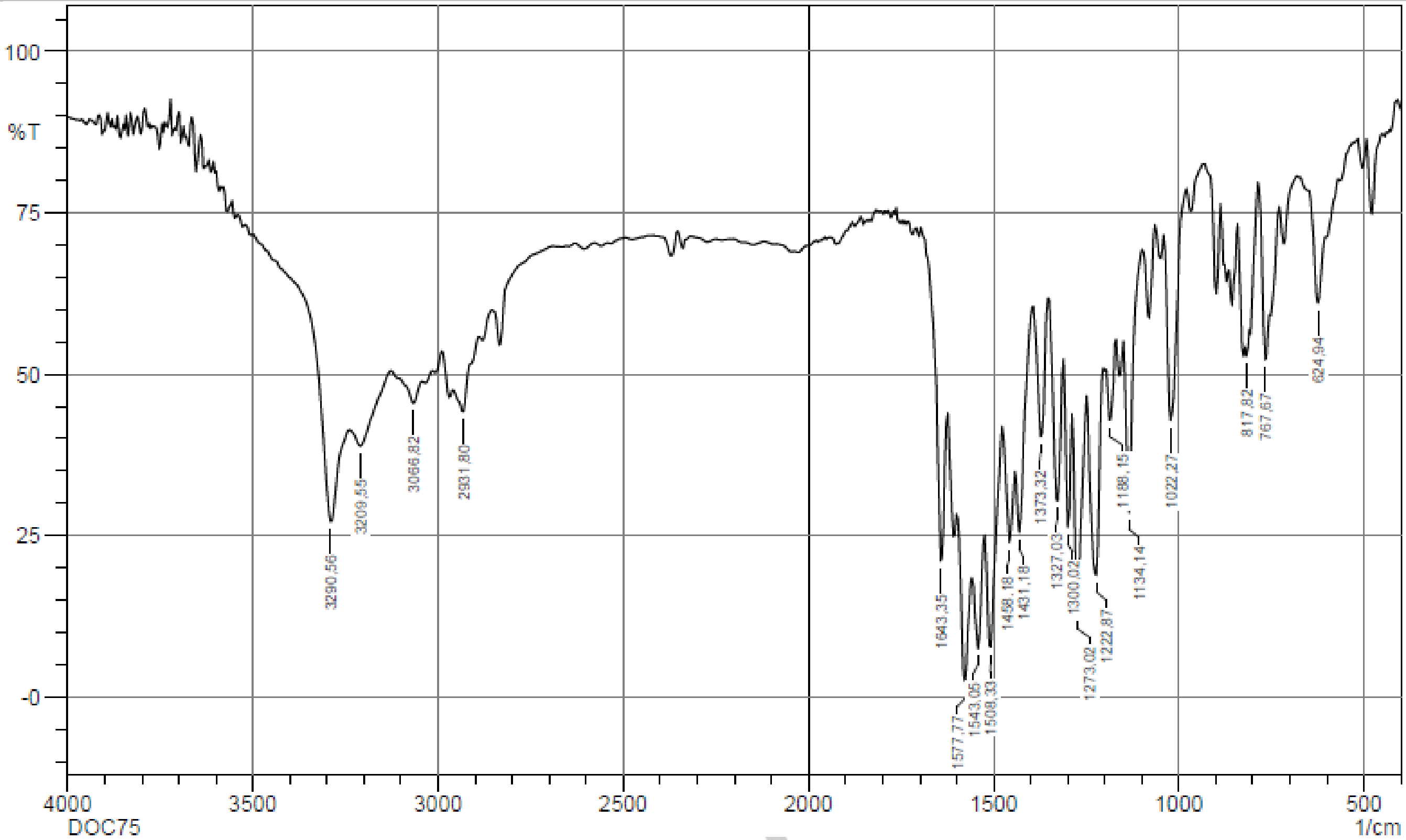

FIGURE S27 IR spectrum (KBr) of compound **10f**.

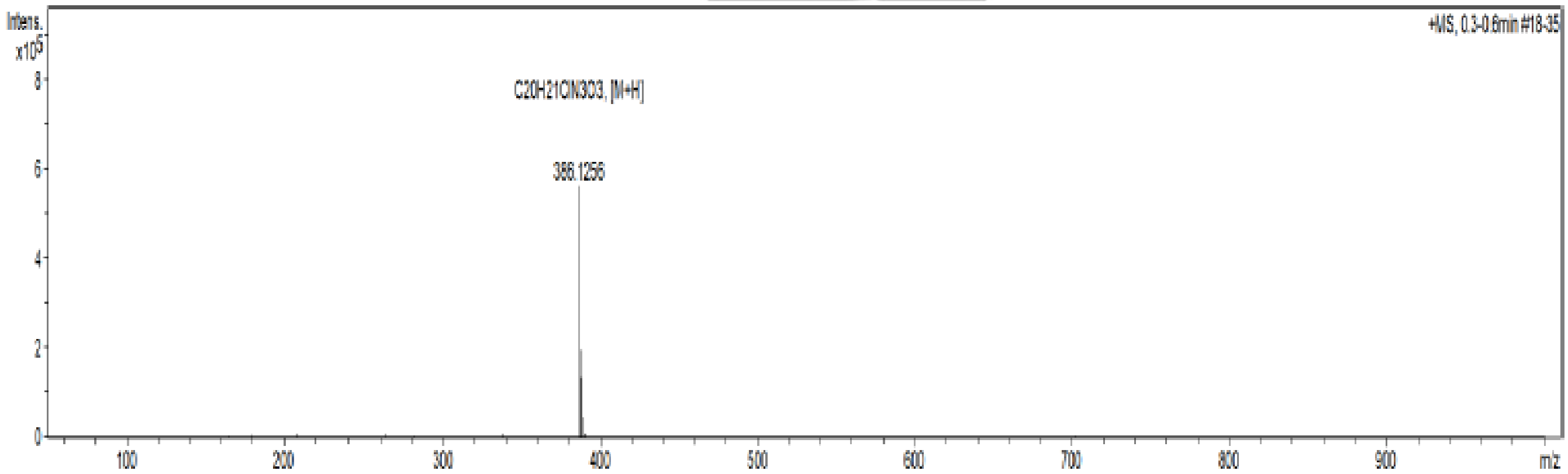

Meas. m/z	#	Ion Formula	m/z	err [ppm]	mSigma	# mSigma	Score	rdb	e⁻ Conf	N-Rule
386.1256	1	C20H21ClN3O3	386.1266	2.6	2.6	1	100.00	11.5	even	ok

FIGURE S28 HRMS spectrum (ESI) of compound **10f**.

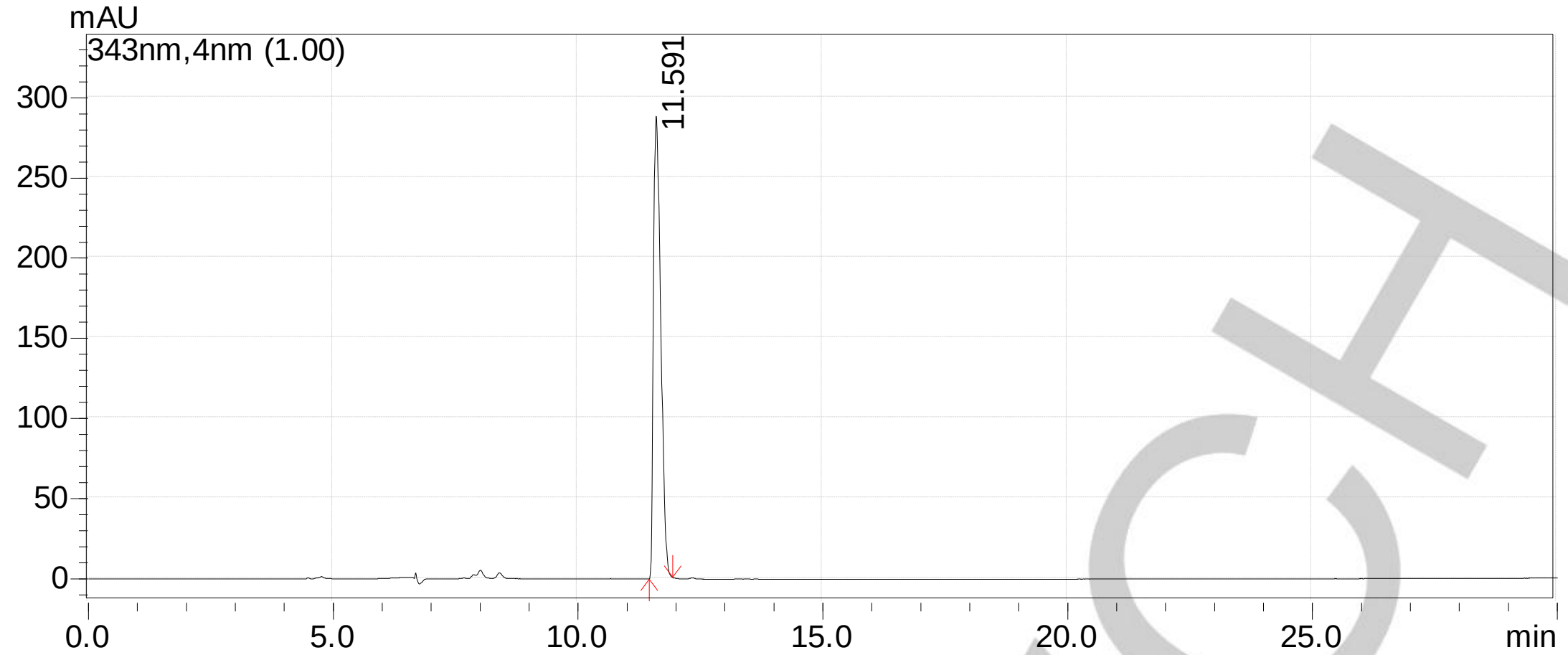

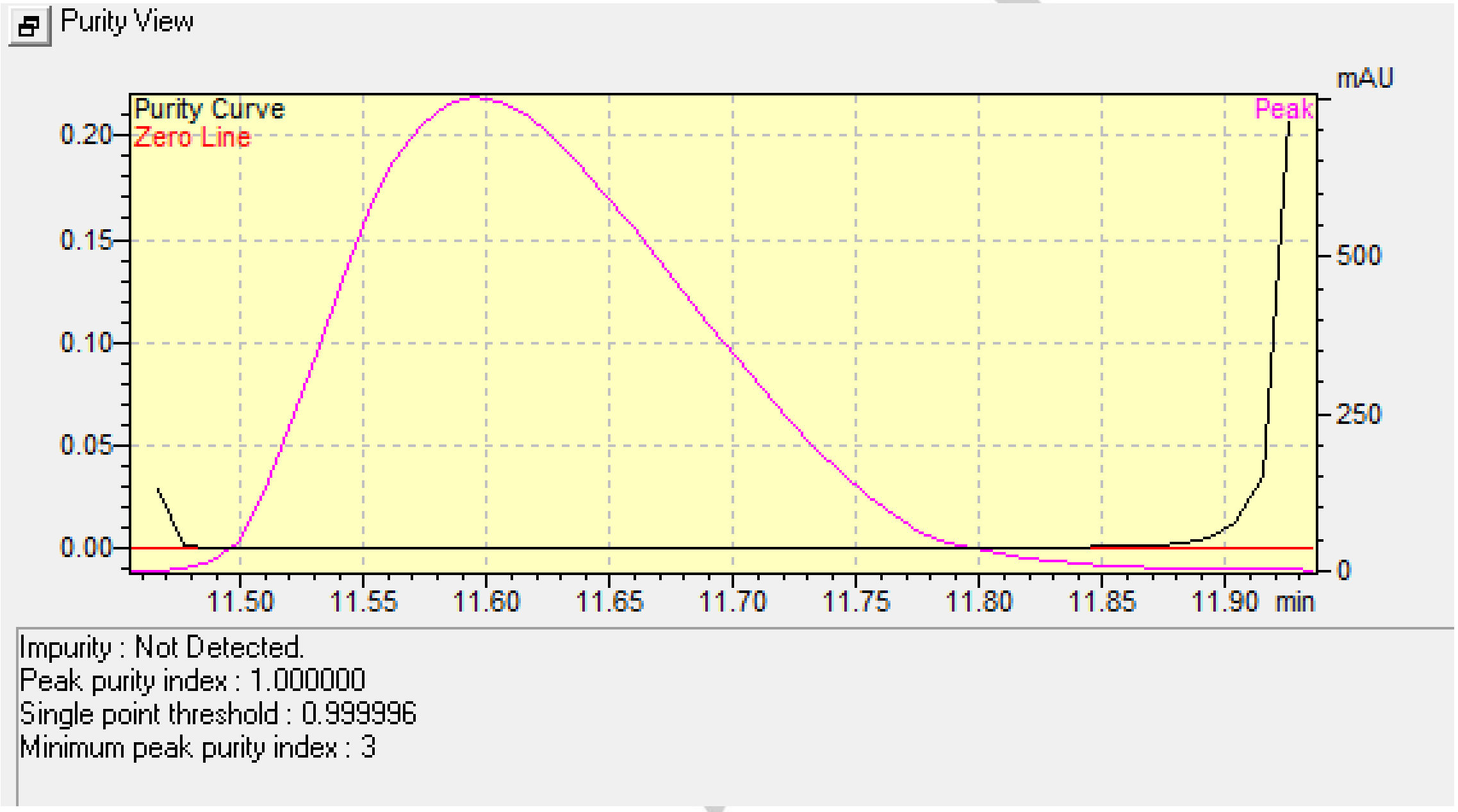

FIGURE S29 HPLC chromatrogram of compound **10f**.

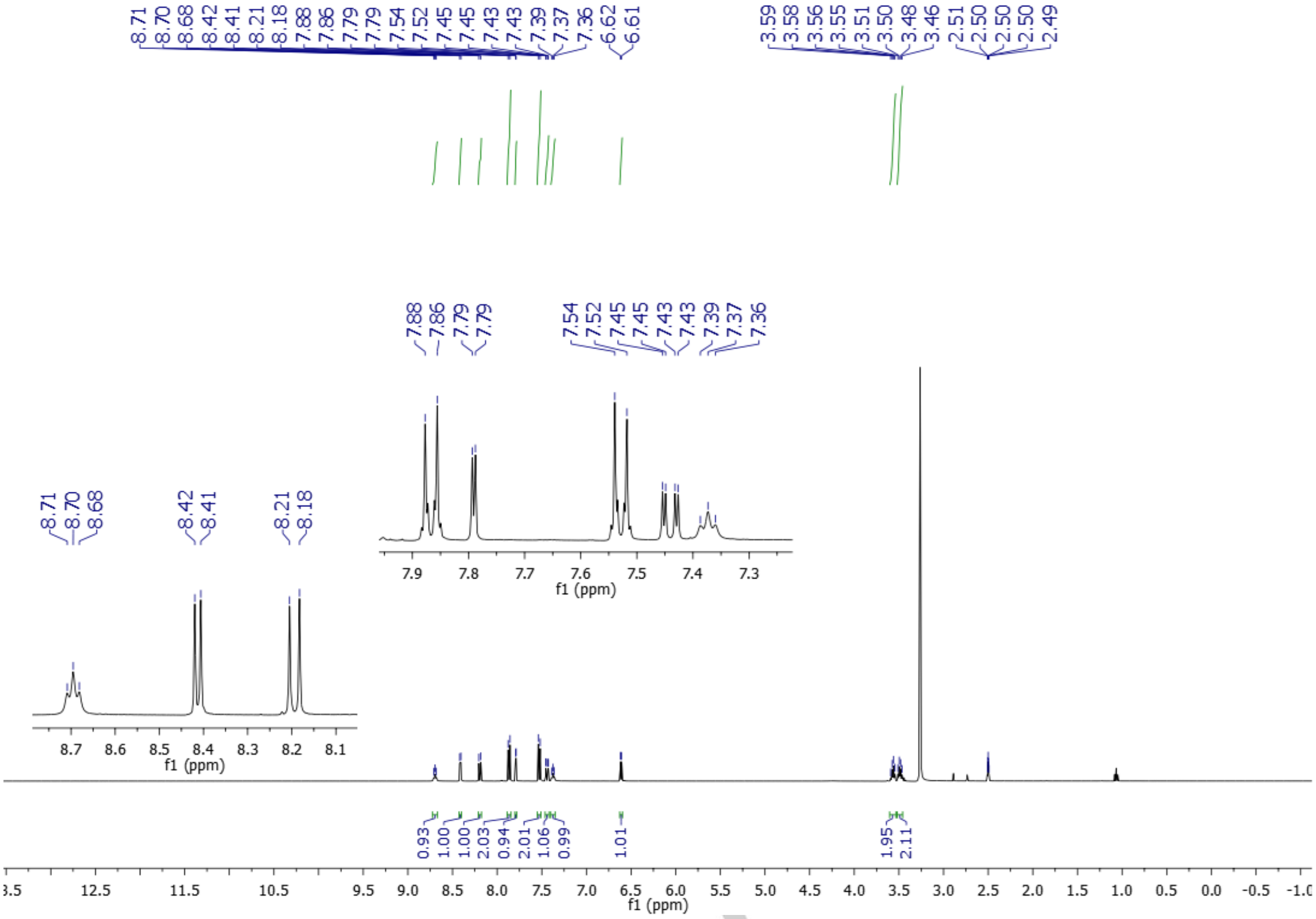

FIGURE S30 ^{1}H NMR spectrum (DMSO-d_6, 400 MHz) of compound **10g**.

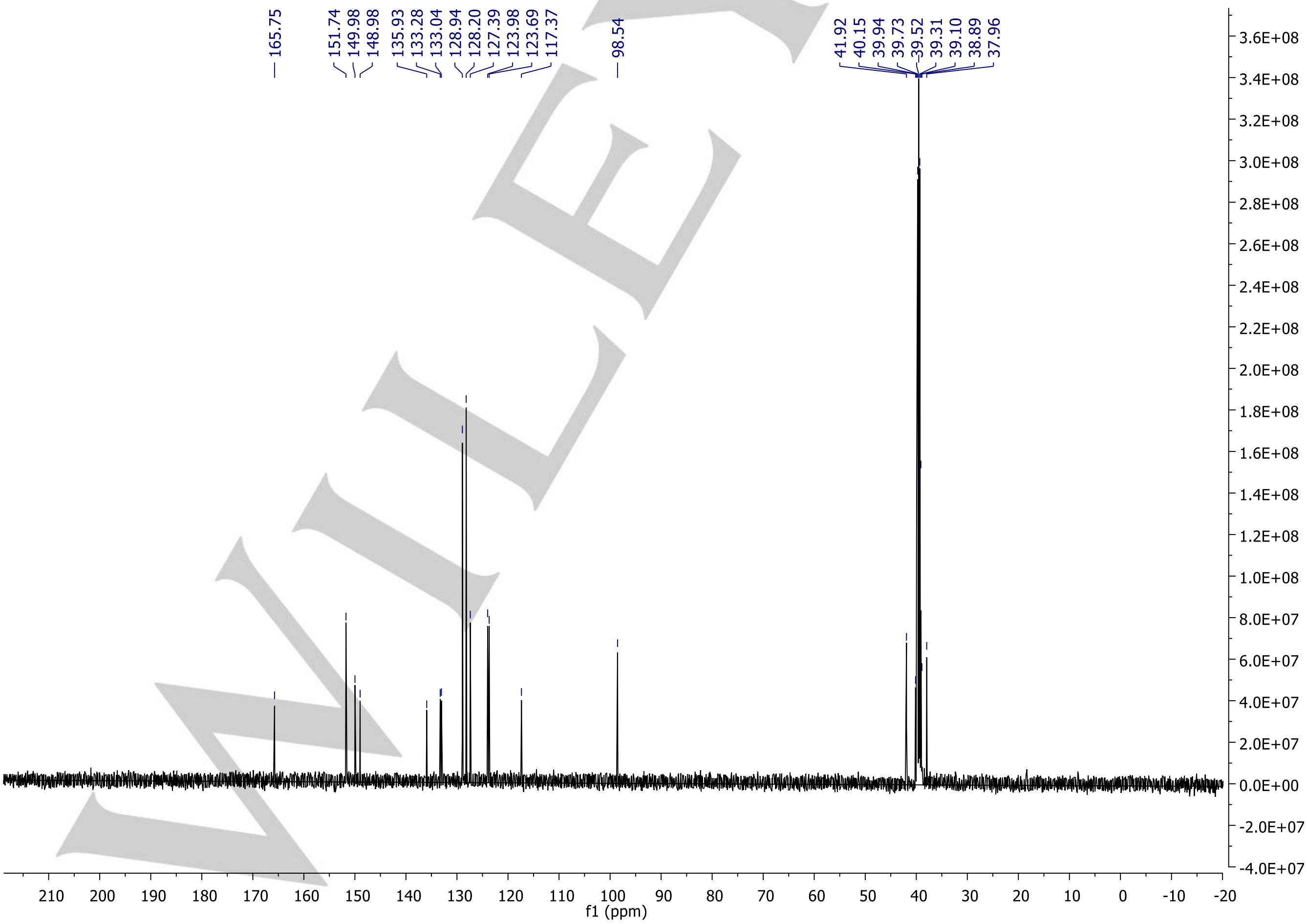

FIGURE S31 ^{13}C NMR spectrum (DMSO-d_6, 101 MHz) of compound **10g**.

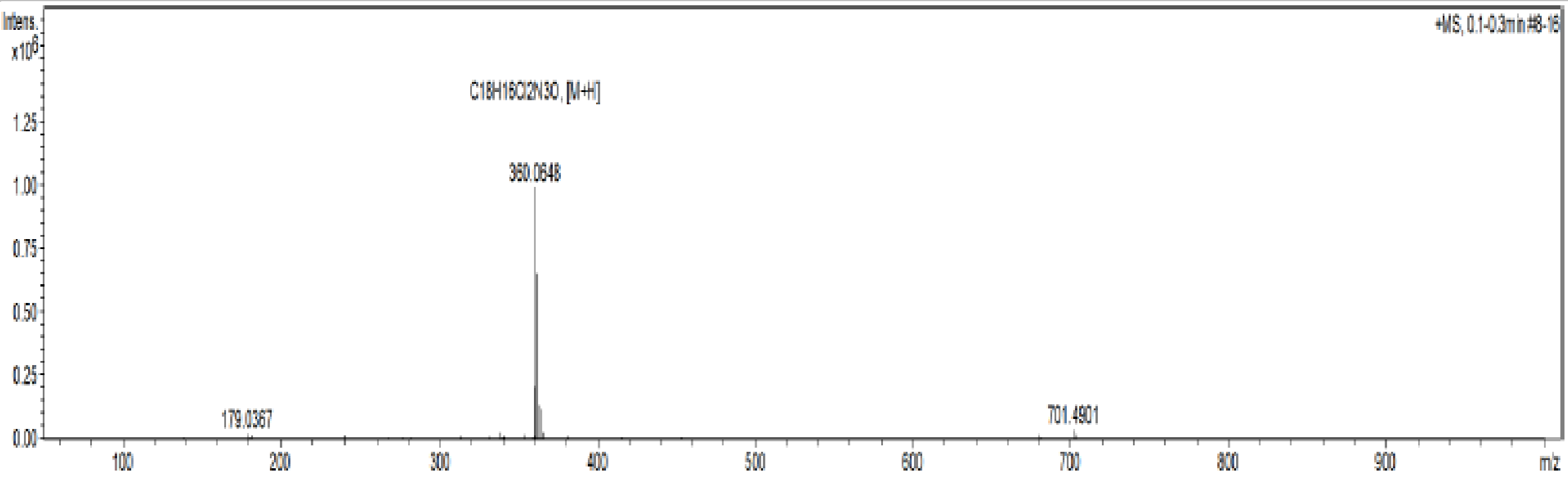

Meas. m/z	#	Ion Formula	m/z	err [ppm]	mSigma	# mSigma	Score	rdb	e¯ Conf	N-Rule
360.0648	1	C18H16Cl2N3O	360.0665	4.8	1.1	1	100.00	11.5	even	ok

FIGURE S32 HRMS spectrum (ESI) of compound **10g**.

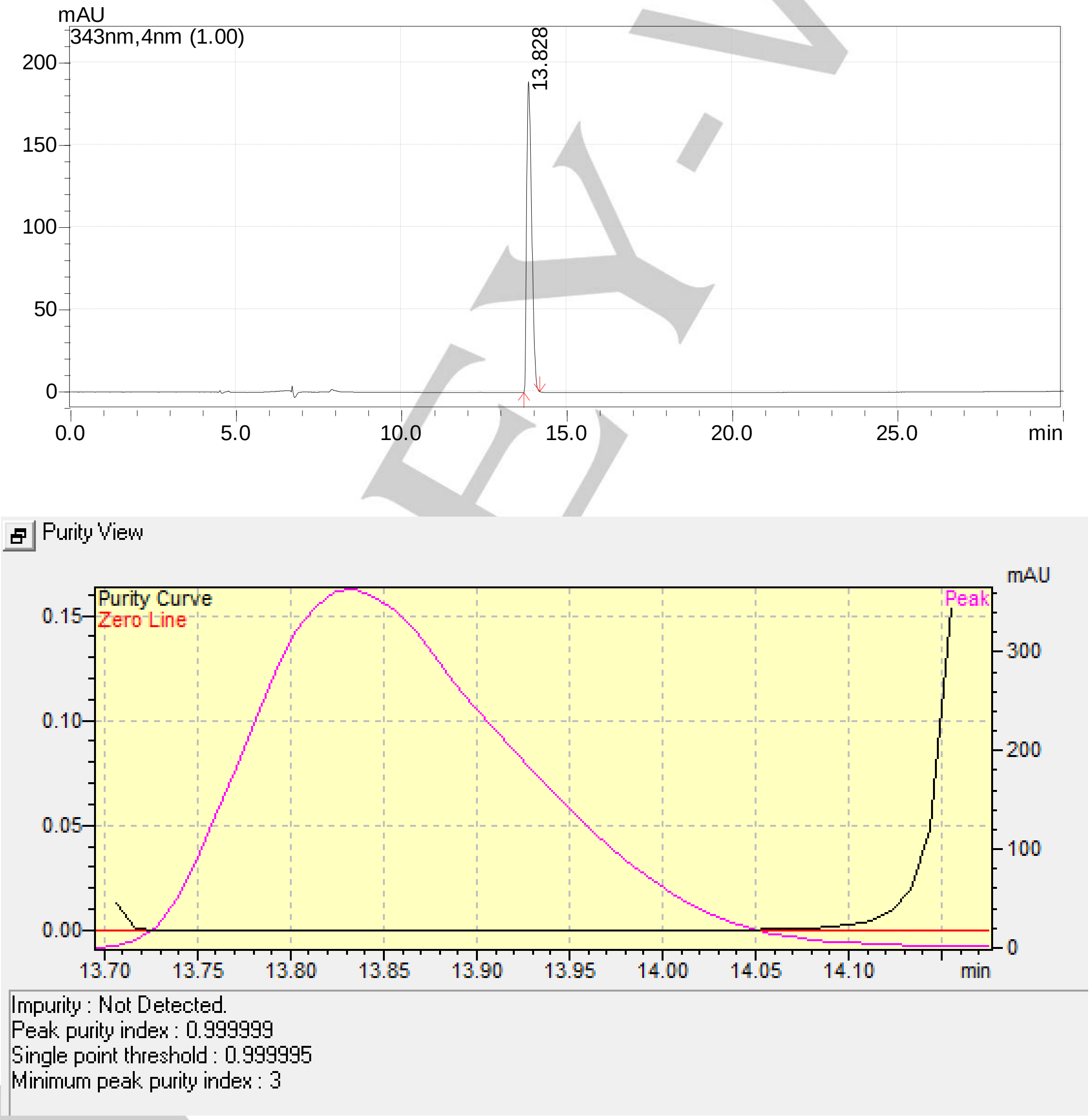

FIGURE S33 HPLC chromatrogram of compound **10g**.

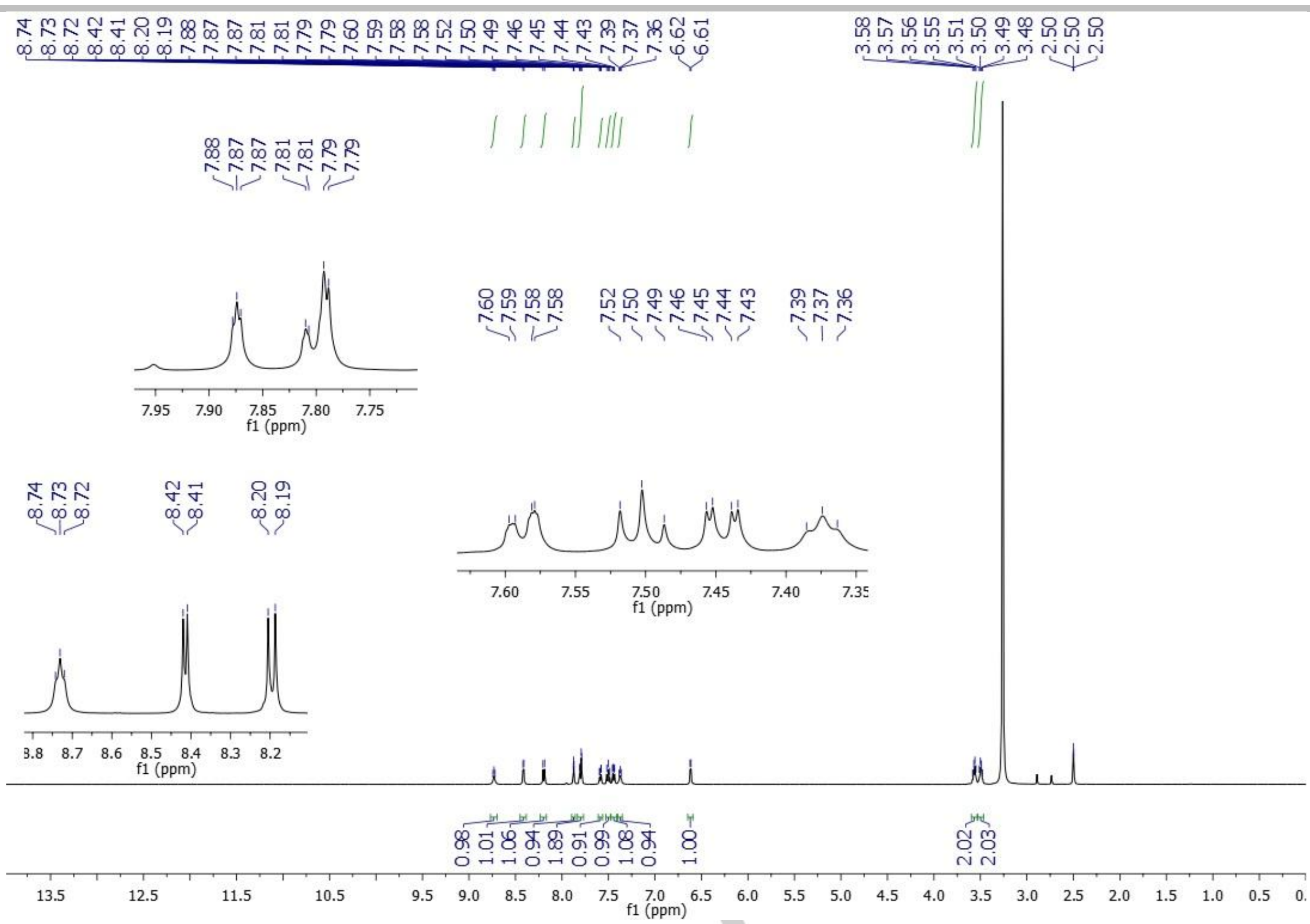

FIGURE S34 ^{1}H NMR spectrum (DMSO-d_6, 500 MHz) of compound **10h**.

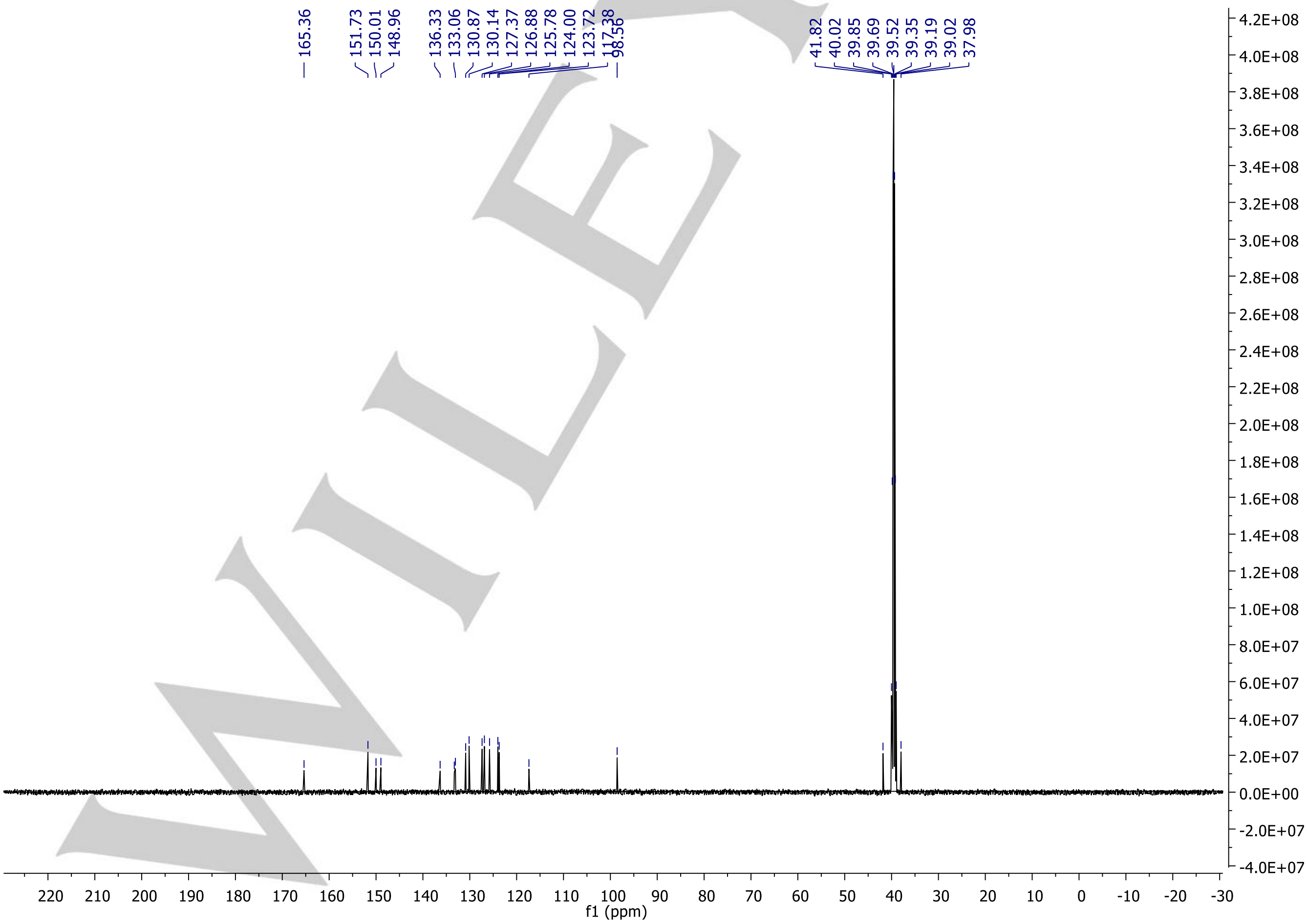

FIGURE S35 ^{13}C NMR spectrum (DMSO-d_6, 126 MHz) of compound **10h**.

WILEY

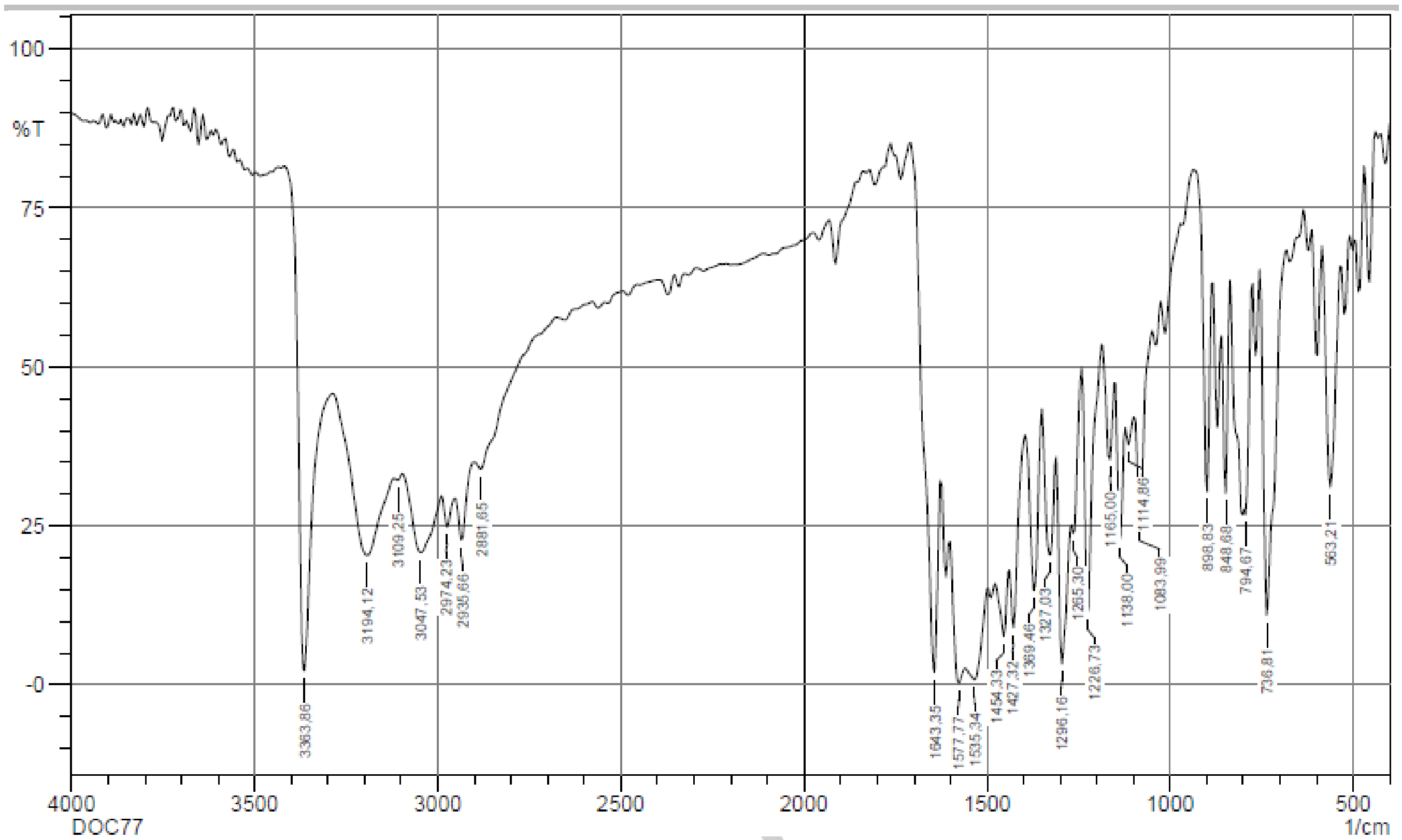

FIGURE S36 IR spectrum (KBr) of compound **10h**.

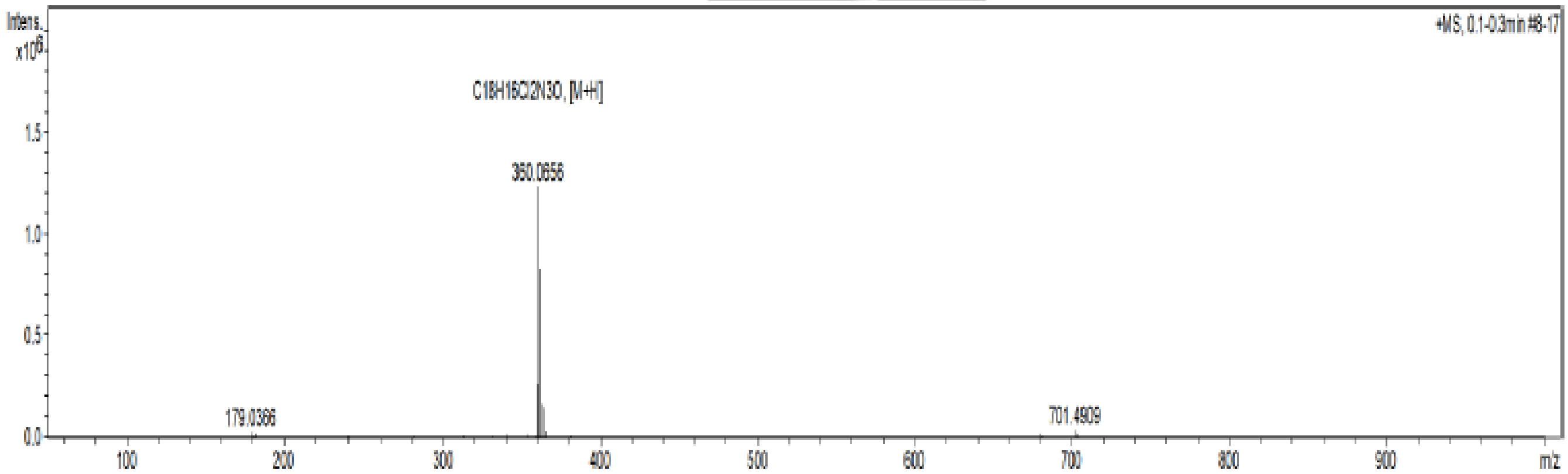

Meas. m/z	#	Ion Formula	m/z	err [ppm]	mSigma	# mSigma	Score	rdb	e¯ Conf	N-Rule
360.0656	1	C18H16Cl2N3O	360.0665	2.5	3.4	1	100.00	11.5	even	ok

FIGURE S37 HRMS spectrum (ESI) of compound **10h**.

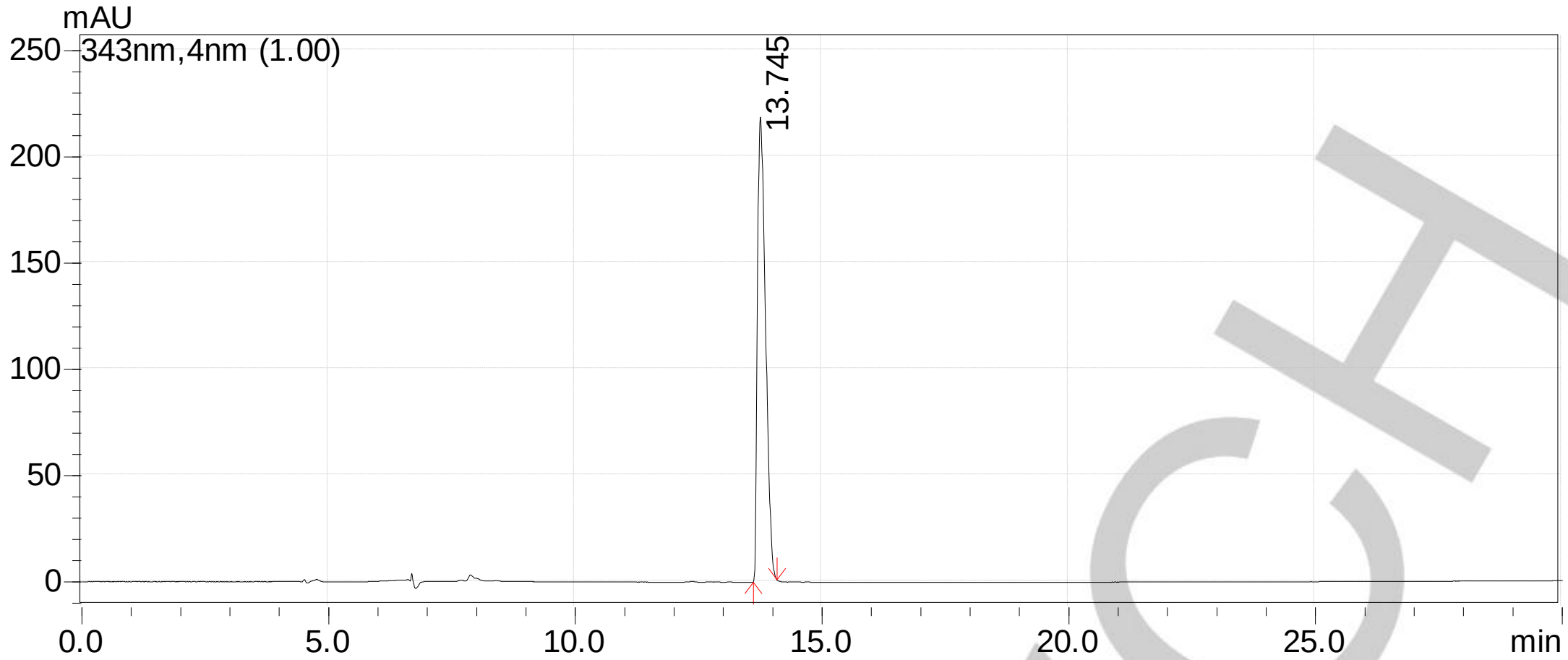

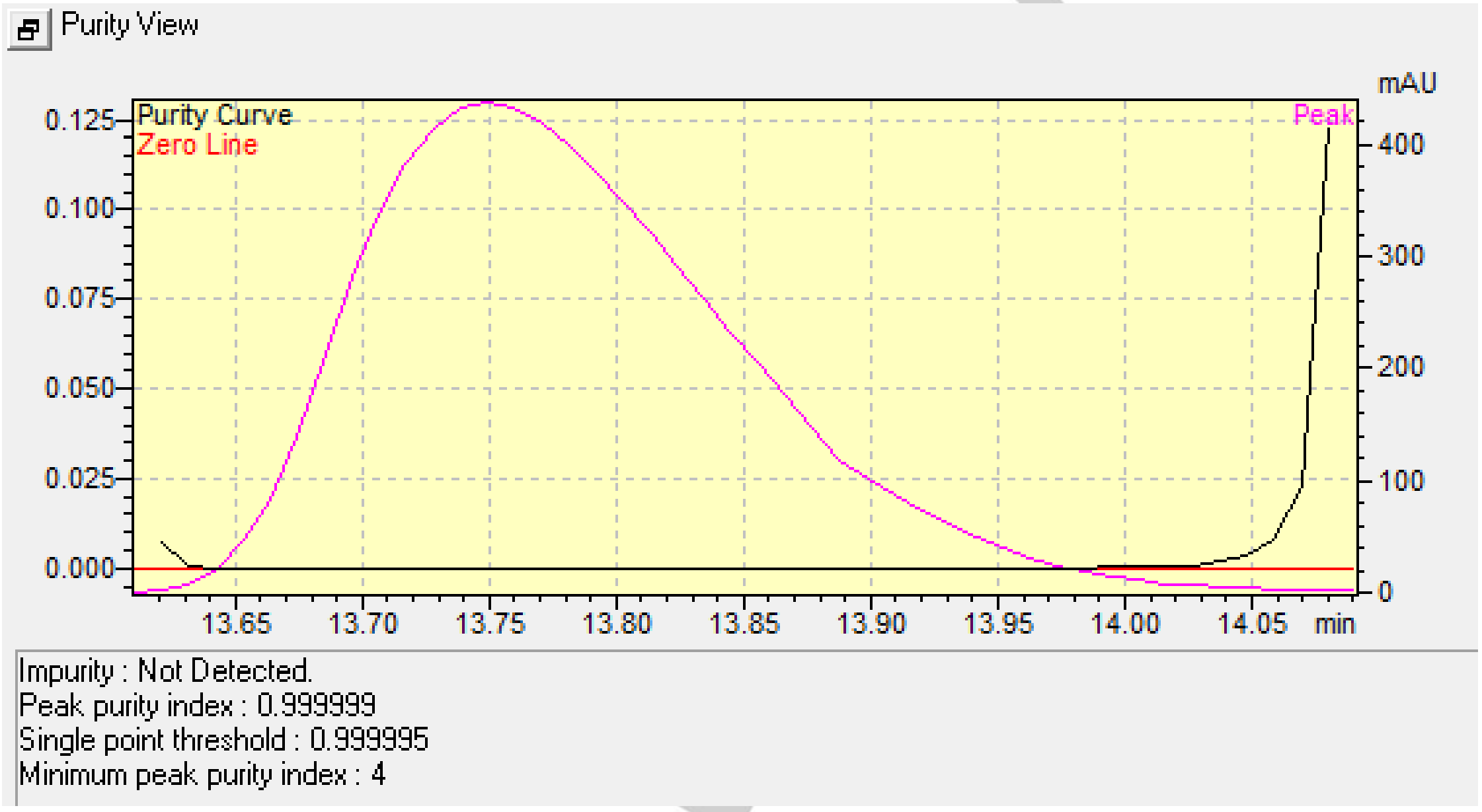

FIGURE S38 HPLC chromatrogram of compound **10h**.

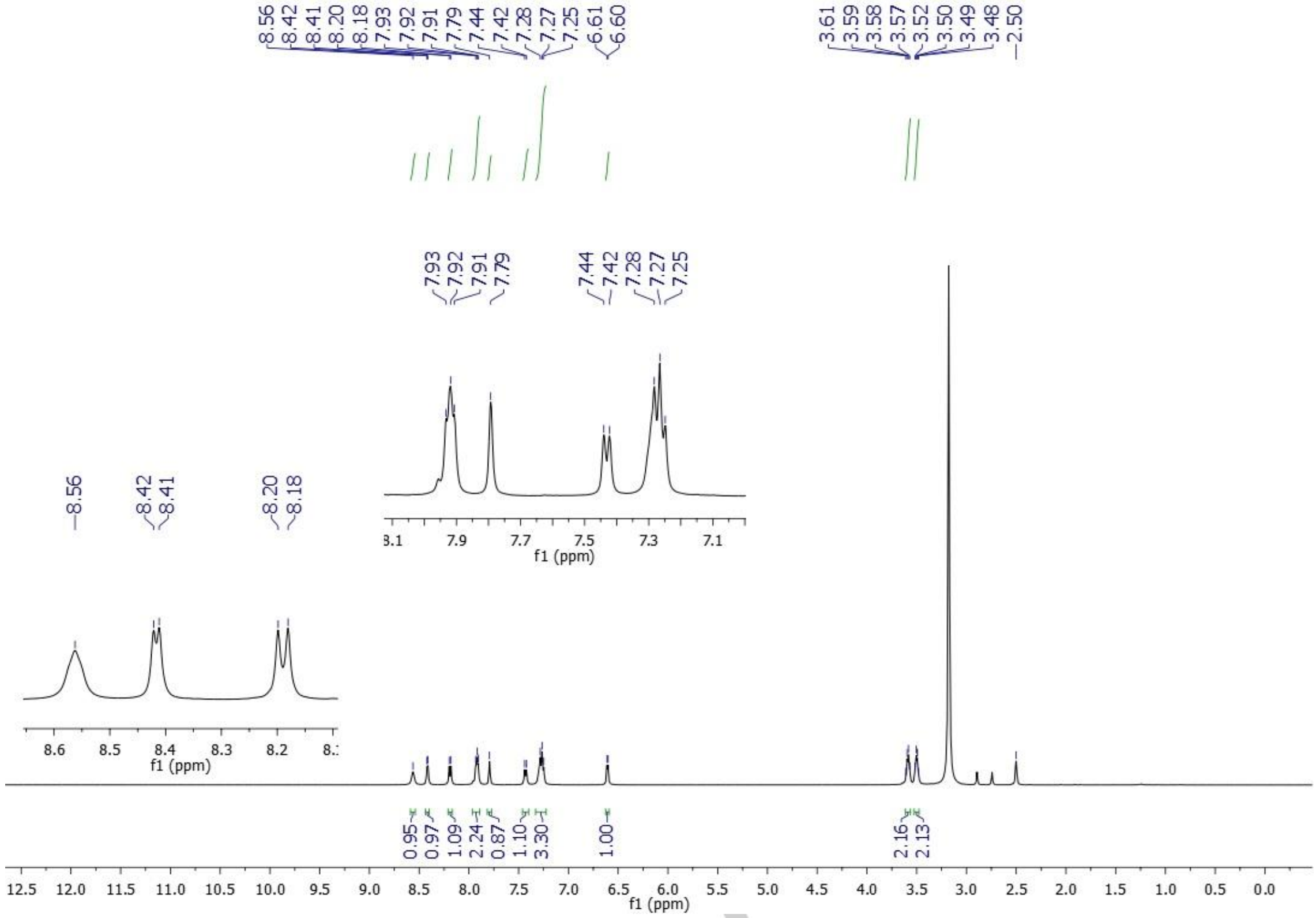

FIGURE S39 ^{1}H NMR spectrum (DMSO-d_6, 500 MHz, 70 °C) of compound **10i**.

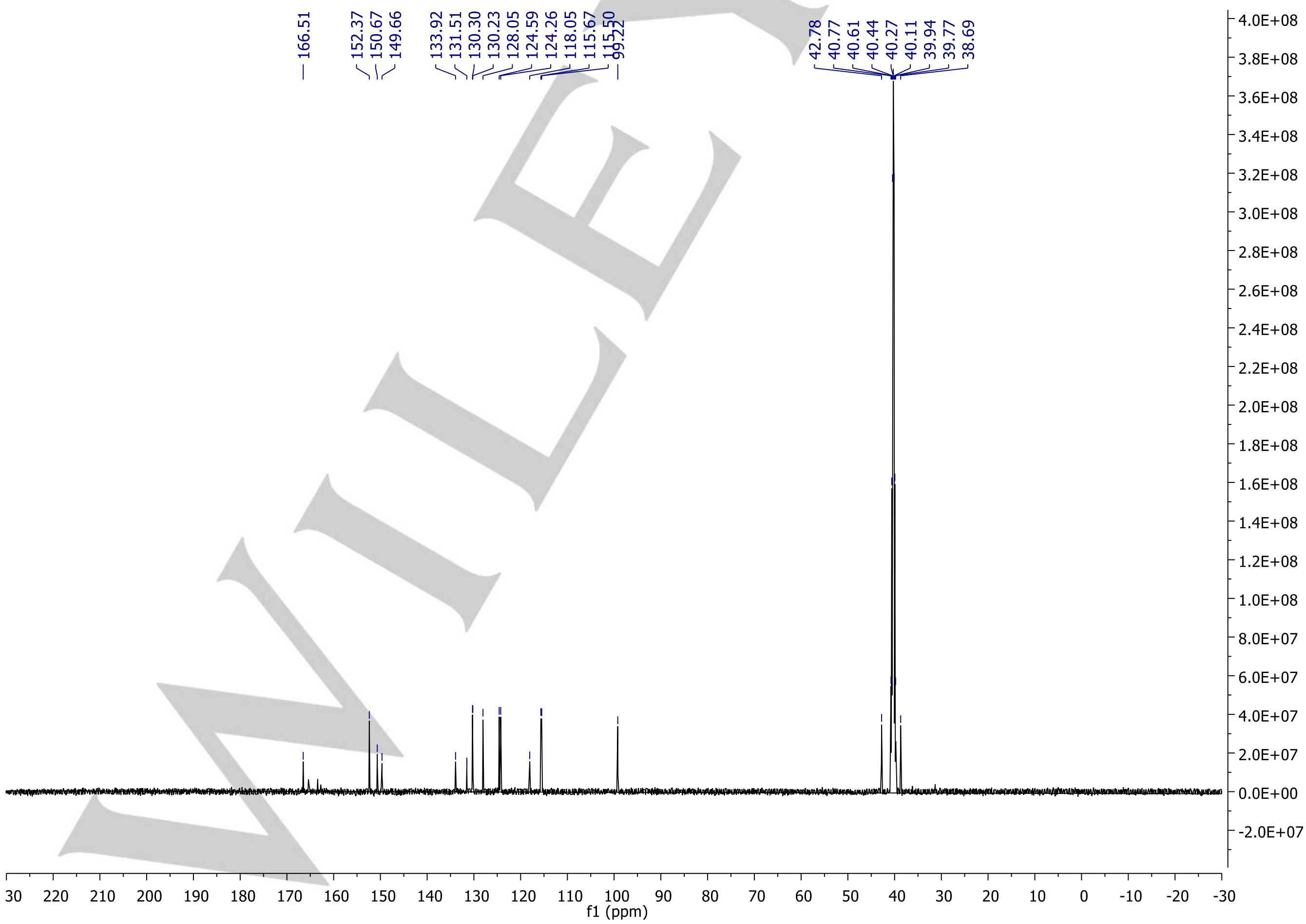

FIGURE S40 ^{13}C NMR spectrum (DMSO-d_6, 126 MHz, 70 °C) of compound **10i**.

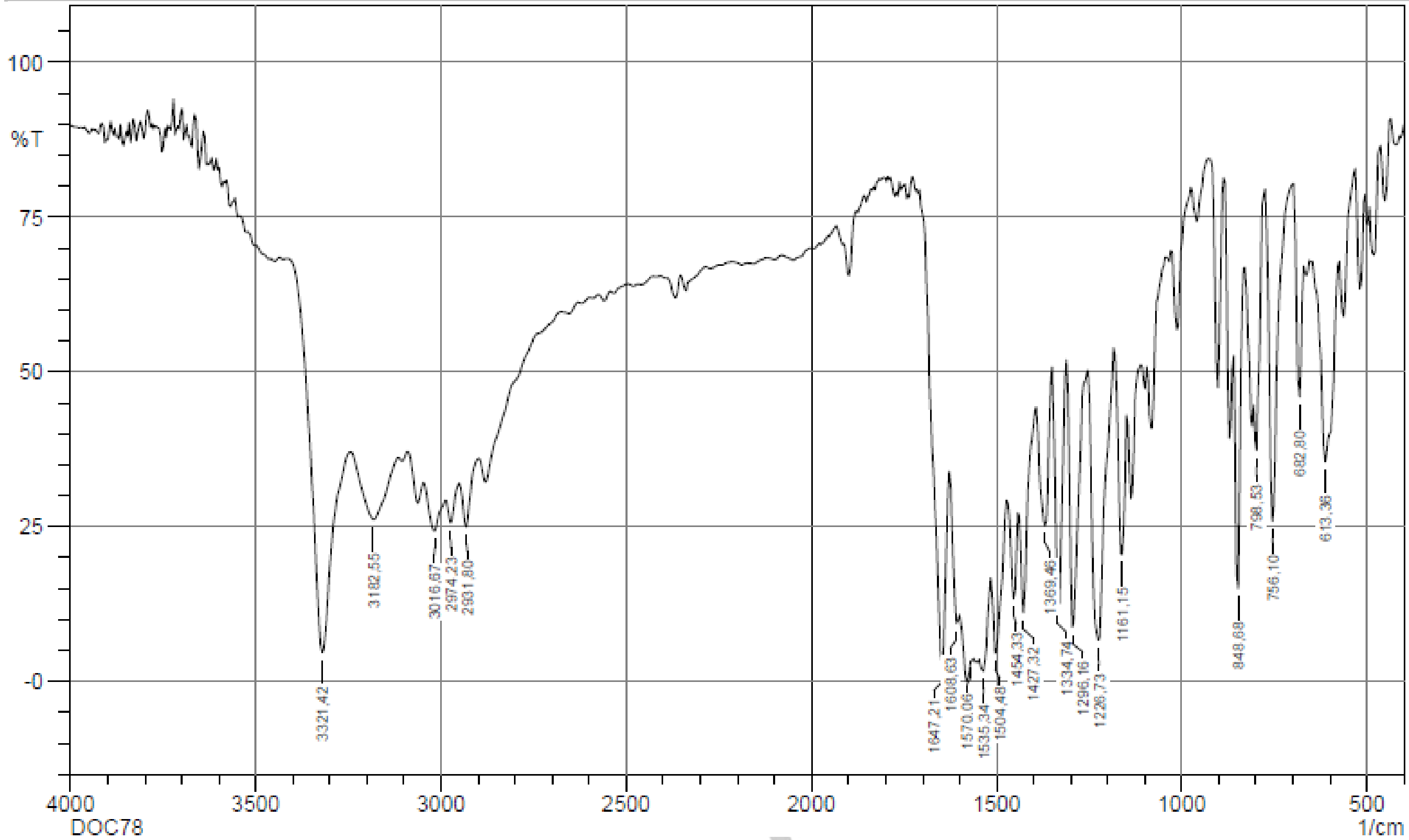

FIGURE S41 IR spectrum (KBr) of compound **10i**.

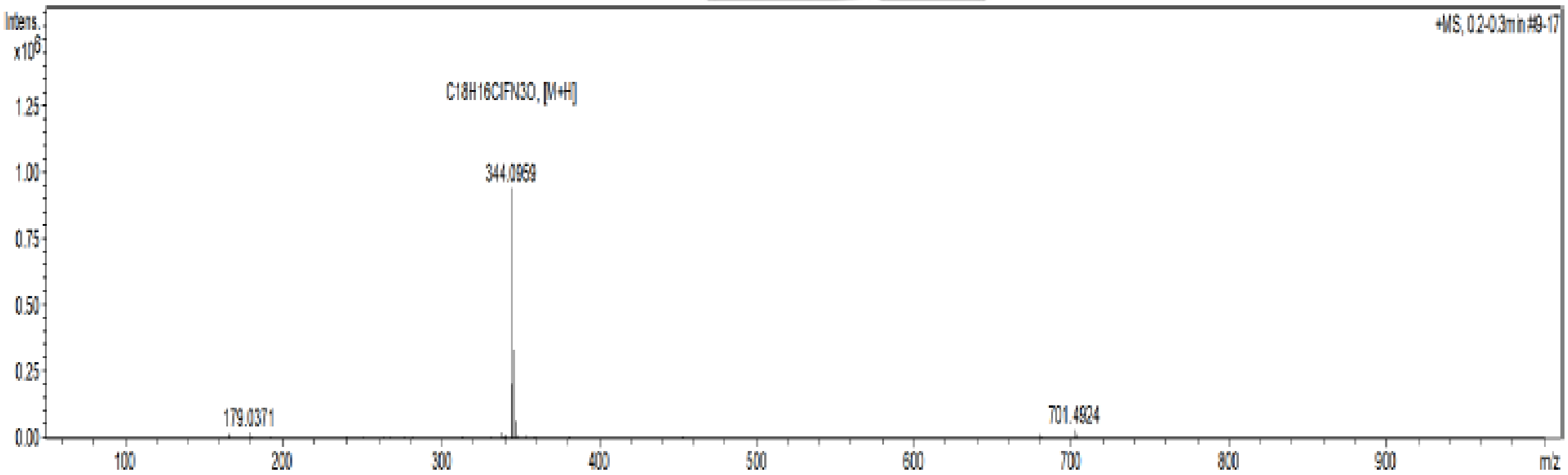

Meas. m/z	#	Ion Formula	m/z	err [ppm]	mSigma	# mSigma	Score	rdb	e¯ Conf	N-Rule
344.0959	1	C18H16ClFN3O	344.0960	0.5	7.0	1	100.00	11.5	even	ok

FIGURE S42 HRMS spectrum (ESI) of compound **10i**.

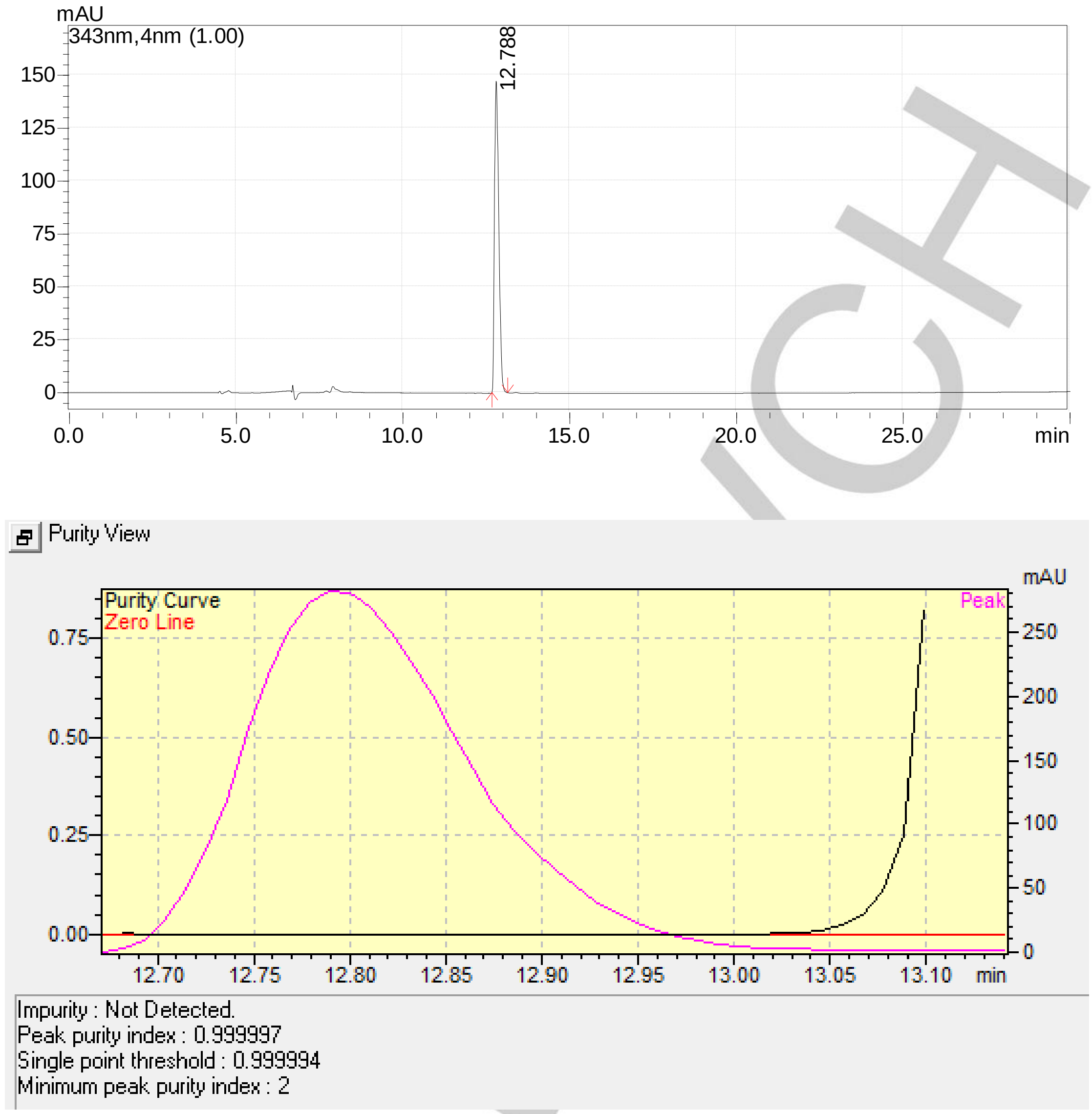

FIGURE S43 HPLC chromatrogram of compound **10i**.

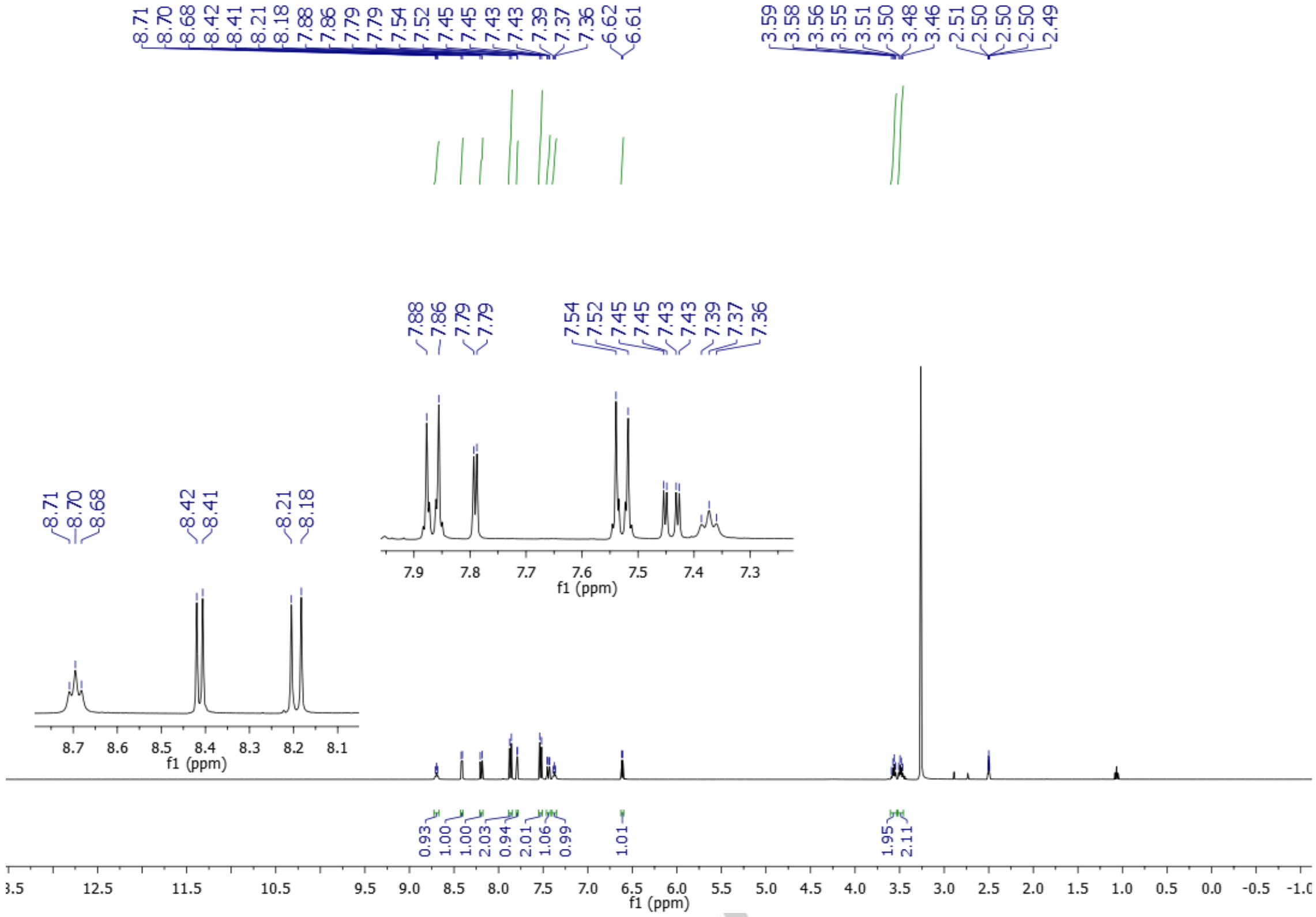

FIGURE S44 ^{1}H NMR spectrum (DMSO-d_6, 400 MHz) of compound **10j**.

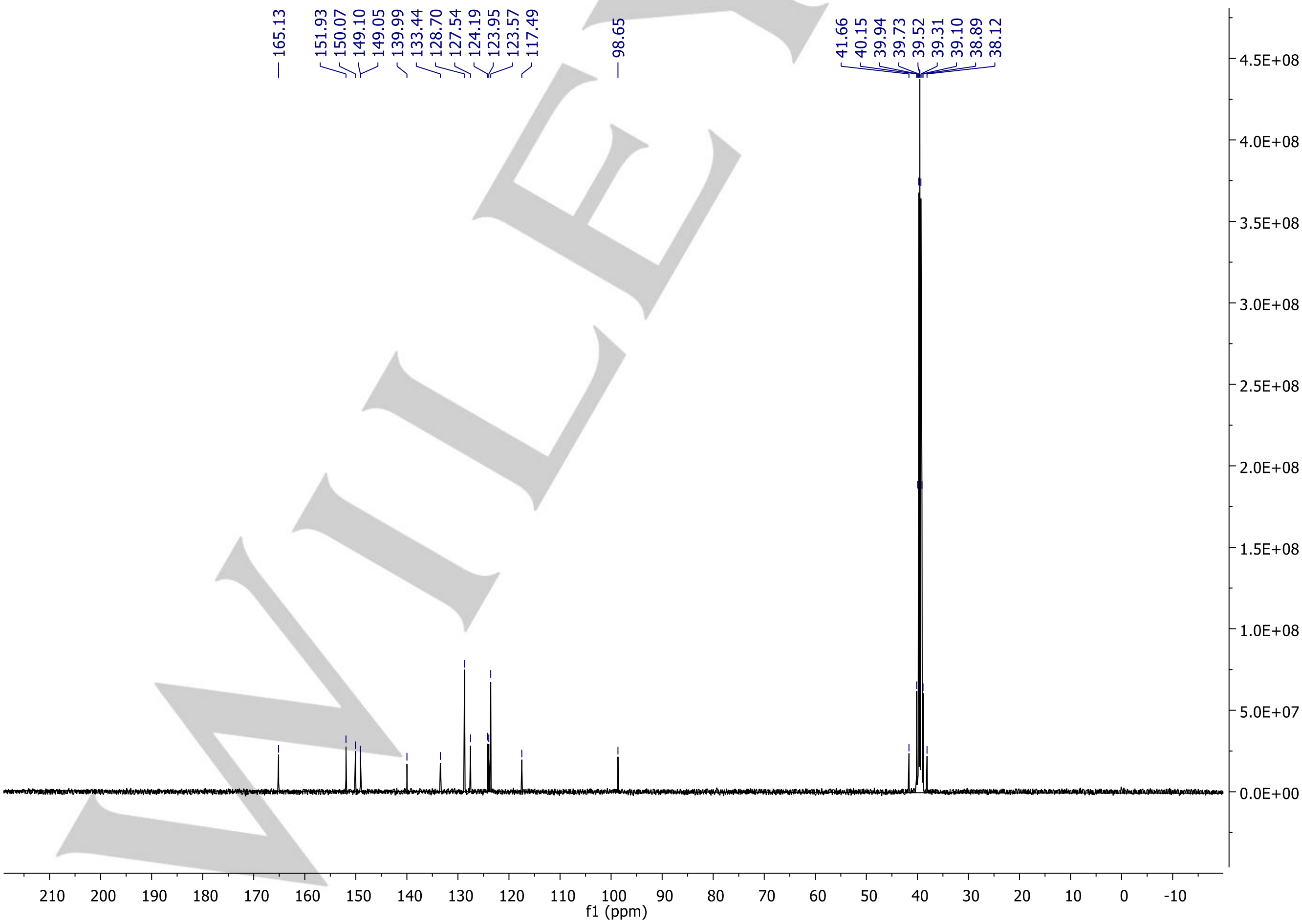

FIGURE S45 ^{13}C NMR spectrum (DMSO-d_6, 101 MHz) of compound **10j**.

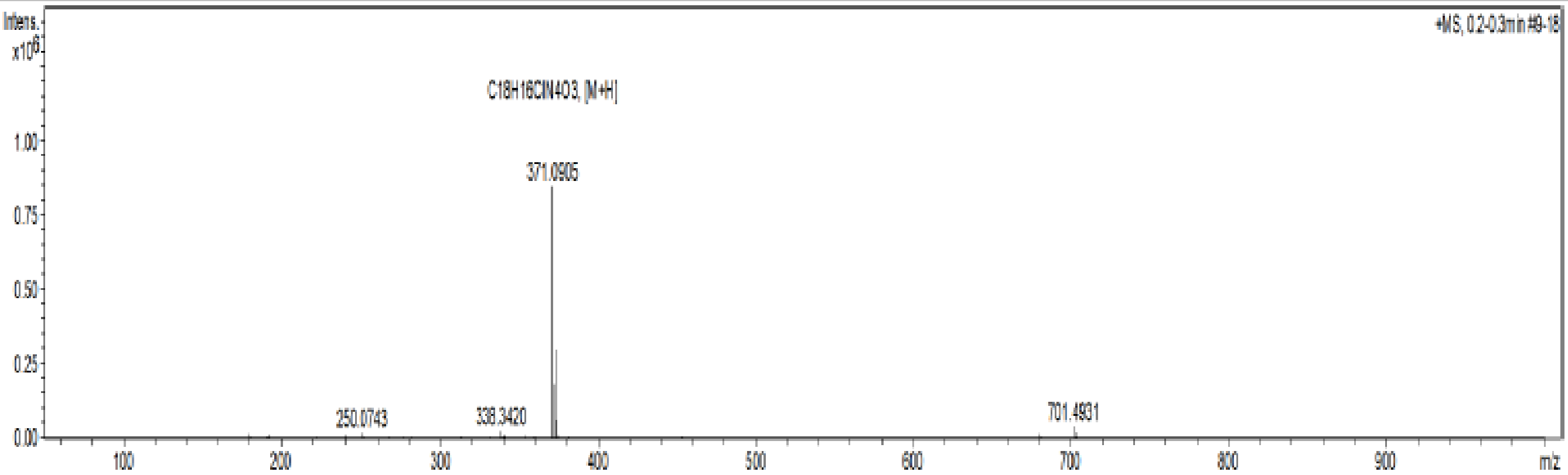

Meas. m/z	#	Ion Formula	m/z	err [ppm]	mSigma	# mSigma	Score	rdb	e¯ Conf	N-Rule
371.0905	1	C18H16ClN4O3	371.0905	0.0	1.2	1	100.00	12.5	even	ok

FIGURE S46 HRMS spectrum (ESI) of compound **10j**.

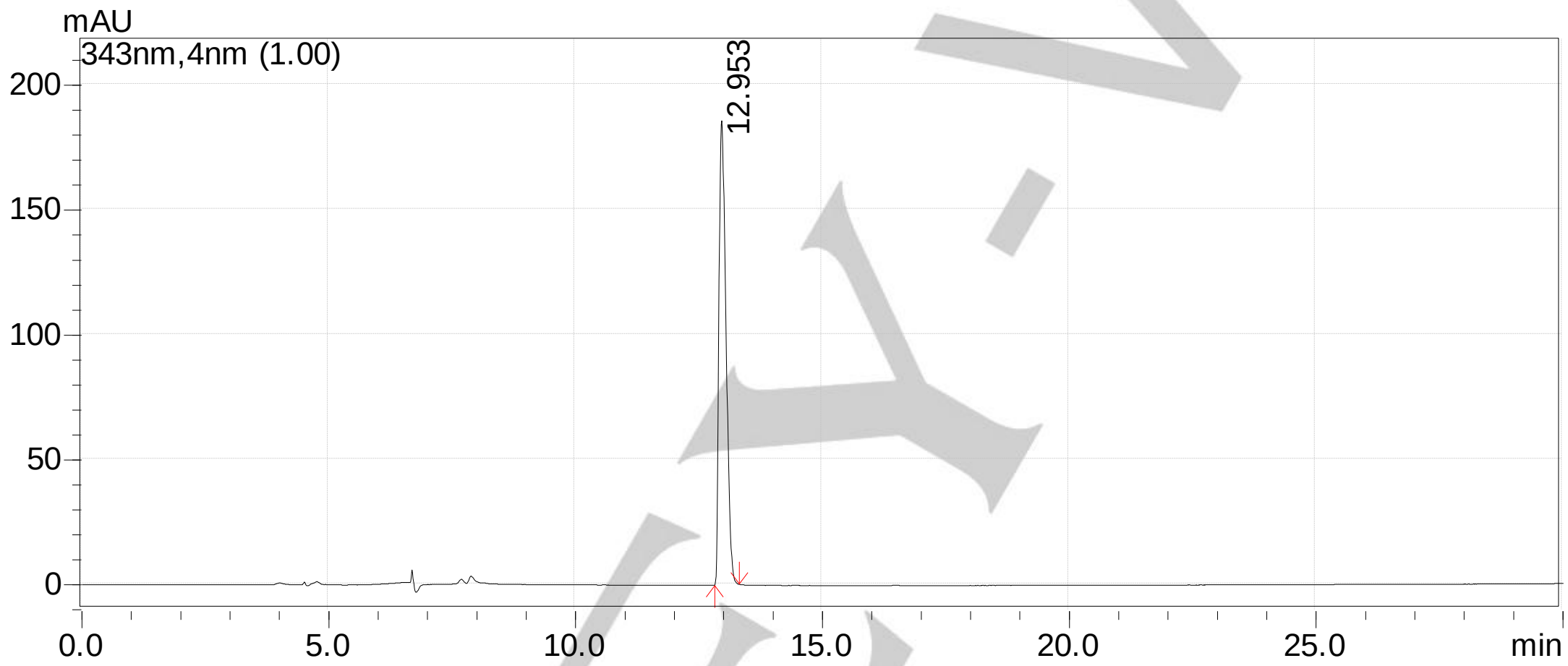

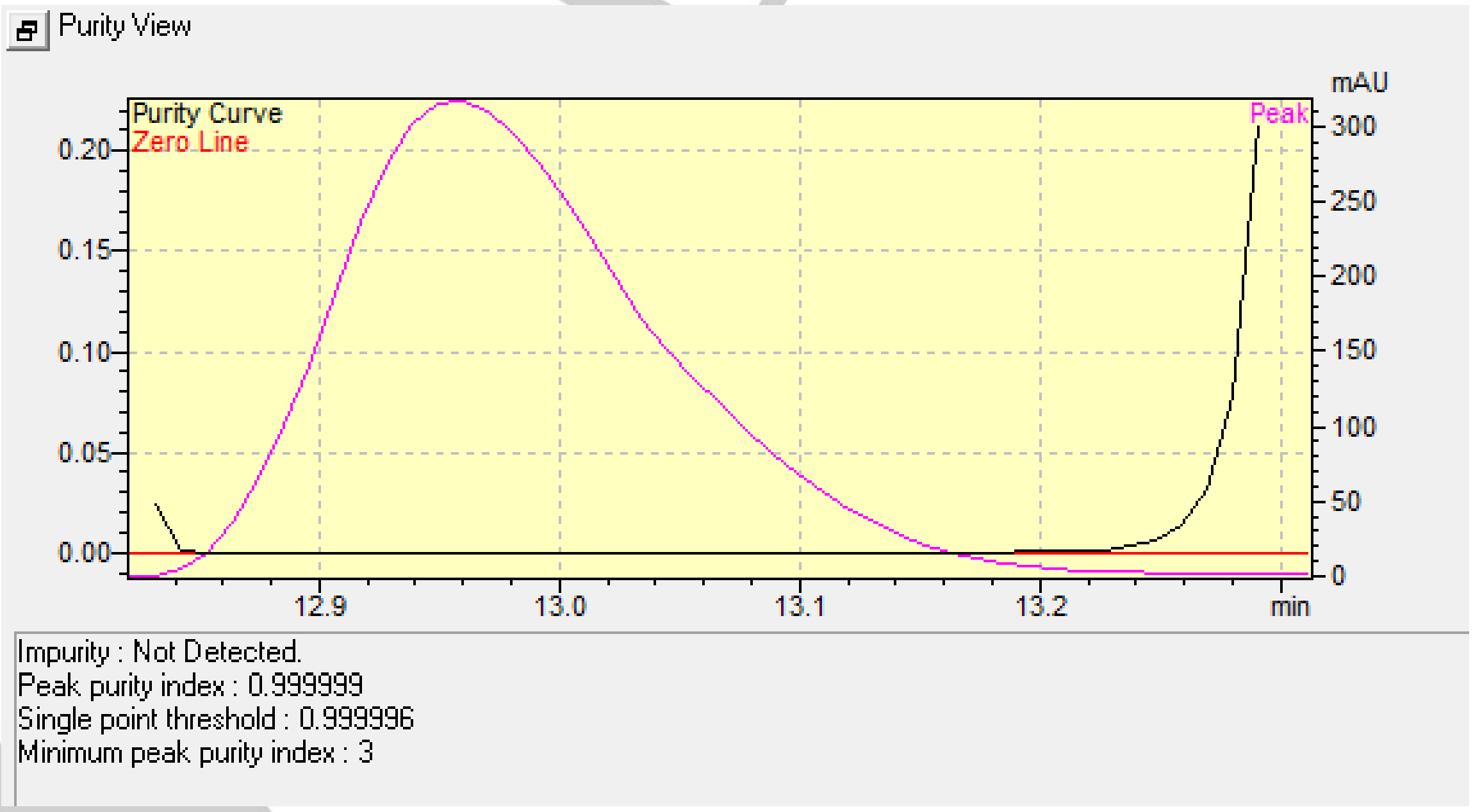

FIGURE S47 HPLC chromatogram of compound **10j**.

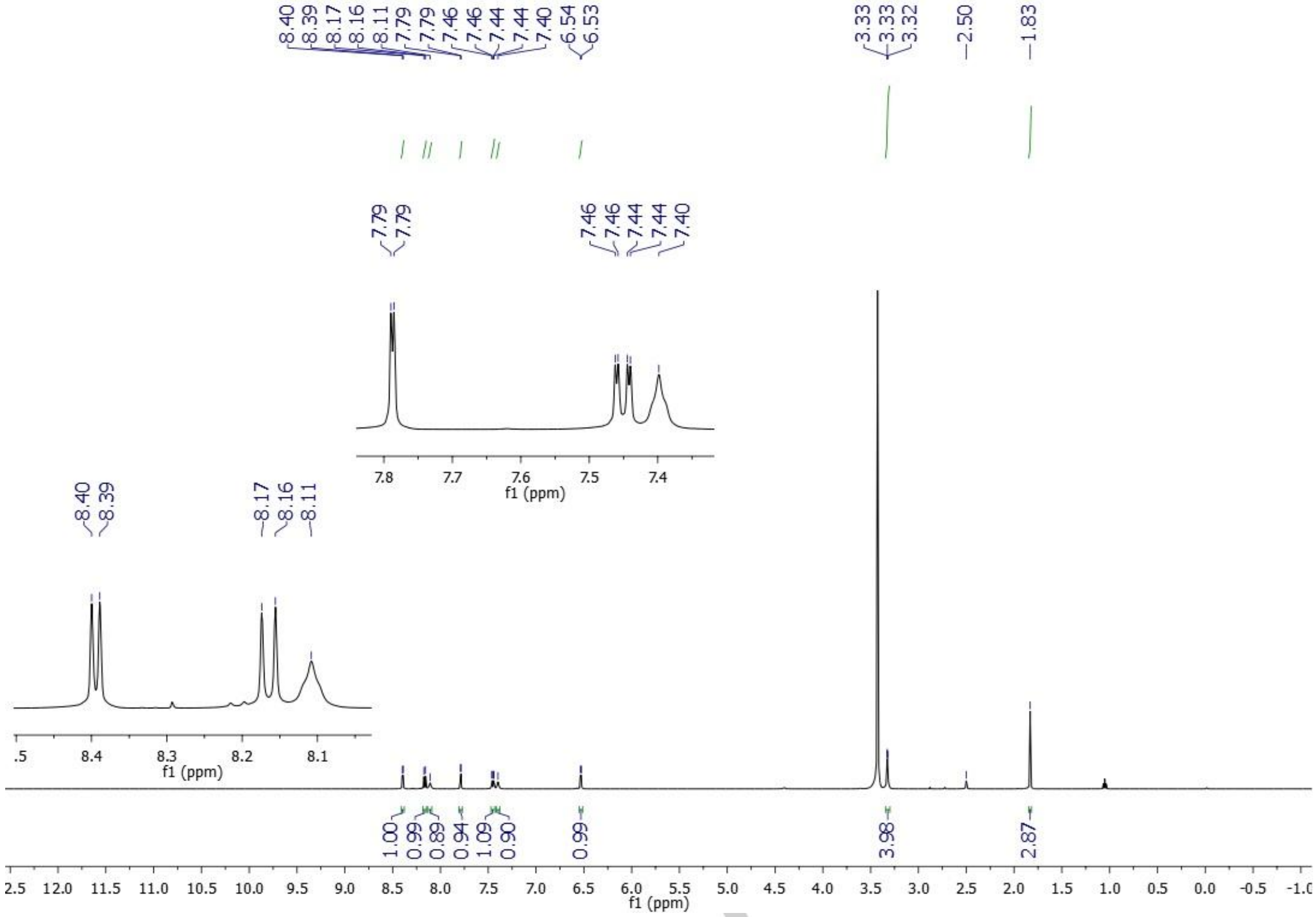

FIGURE S48 ^{1}H NMR spectrum (DMSO-d_6, 400 MHz) of compound **10k**.

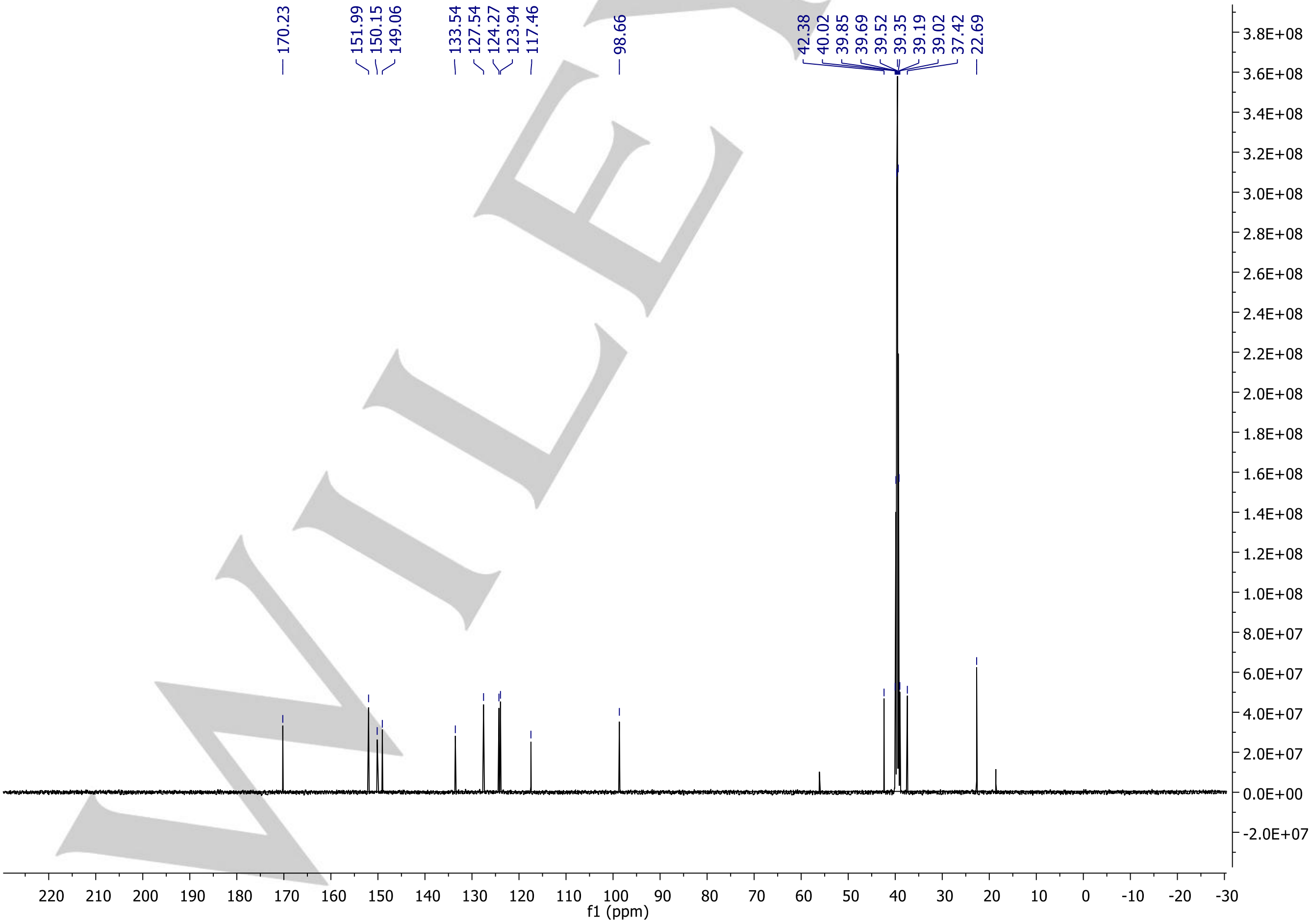

FIGURE S49 ^{13}C NMR spectrum (DMSO-d_6, 101 MHz) of compound **10k**.

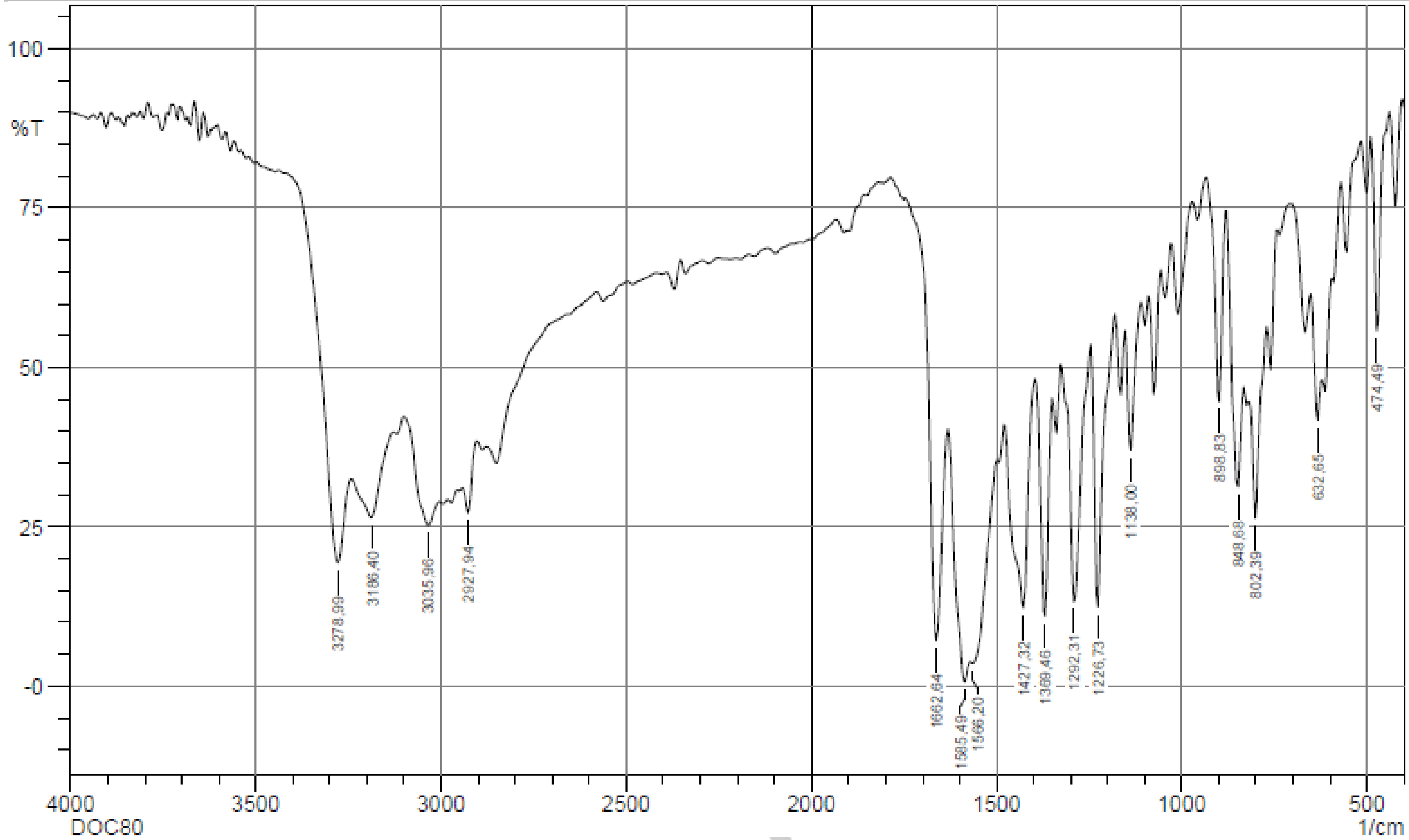

FIGURE S50 IR spectrum (KBr) of compound **10k**.

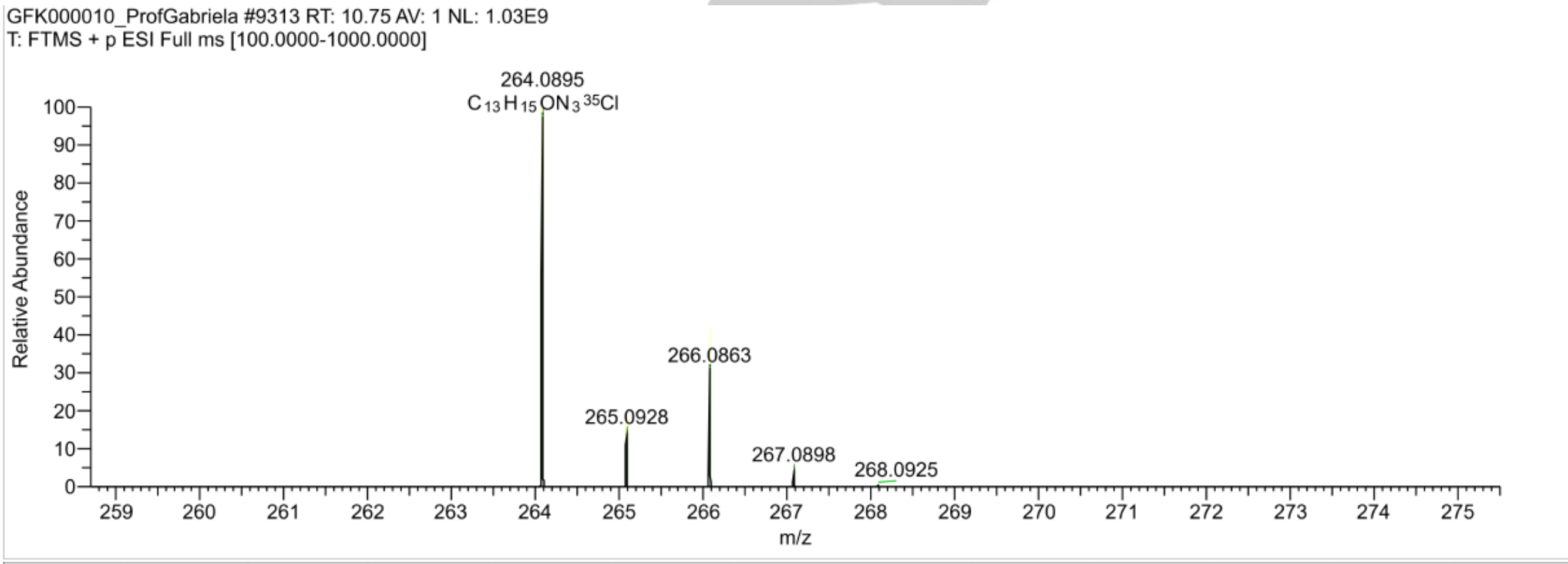

Peak Mass	Display For...	S Fit	RDB	Delta [ppm]	Theo. mass	Rank	Combined S...	# Matched I...	# Missed Iso.	MS Cov. [%]	Pattern Cov....	MSMS Matc...
264.0895	$C_{13}H_{15}ON_3{}^{35}Cl$	77.32050861 48604	7.50	-1.17	264.08982	1	98.81	5	0	100	100	(Collection)

FIGURE S51 HRMS spectrum (ESI) of compound **10k**.

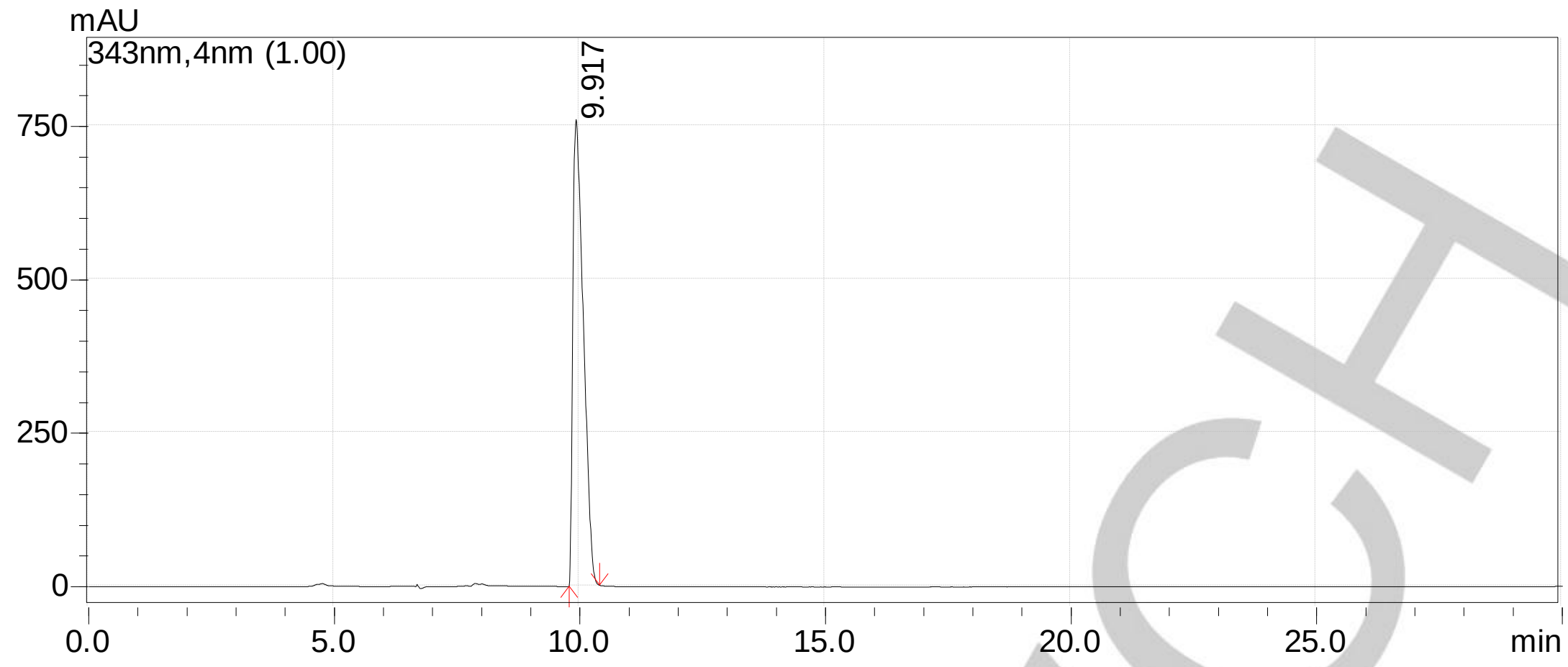

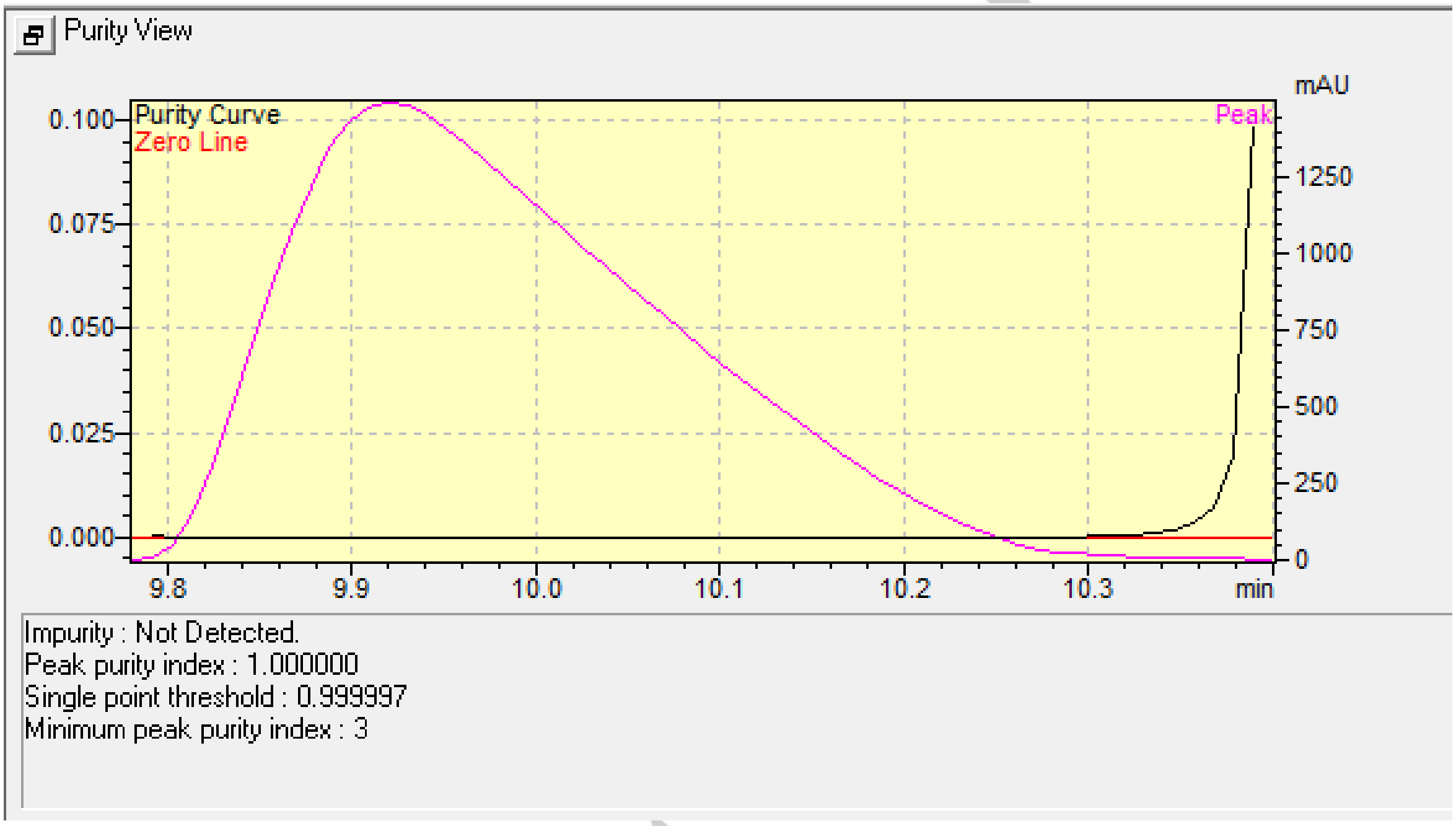

FIGURE S52 HPLC chromatrogram of compound **10k**.

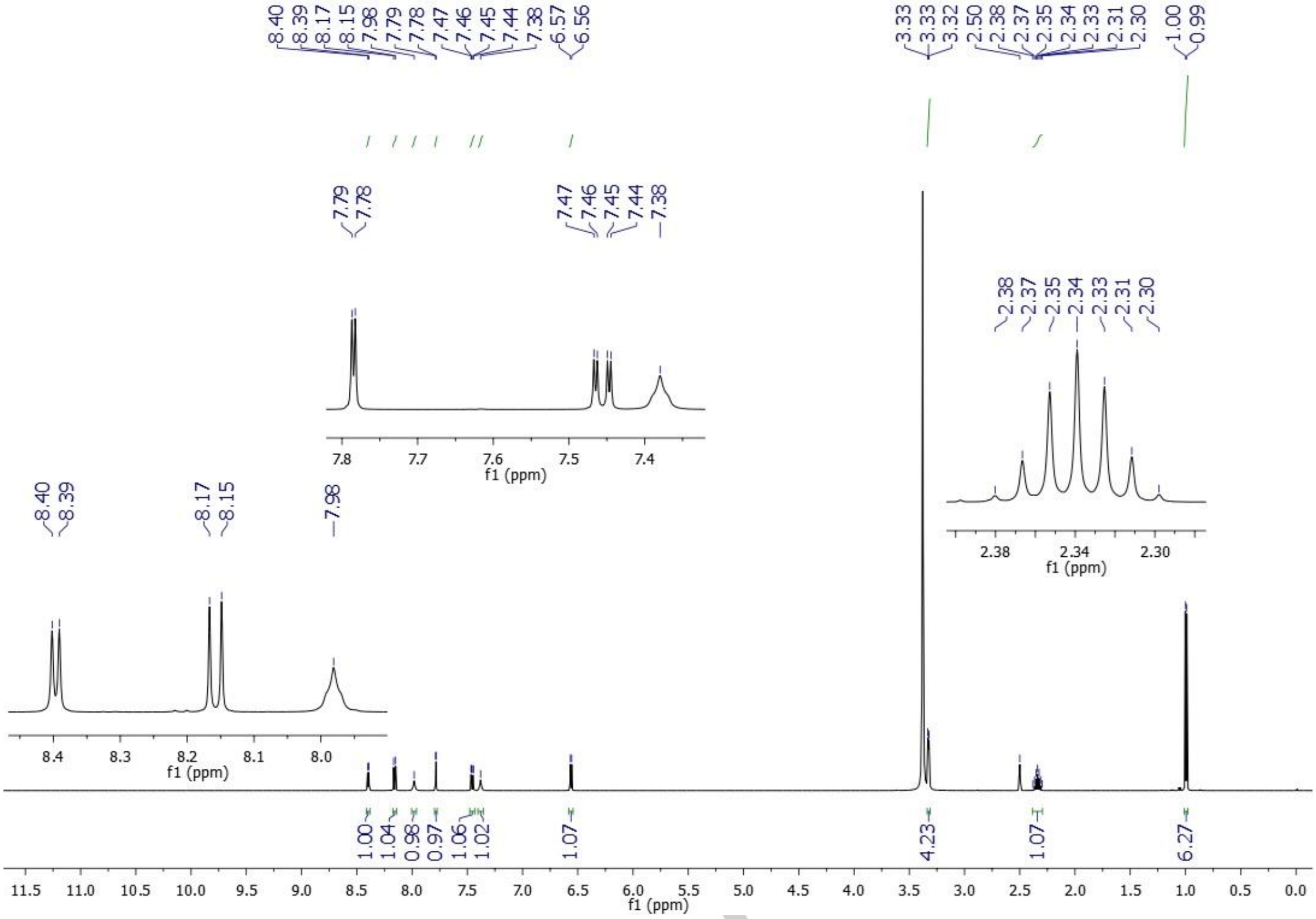

FIGURE S53 ^{1}H NMR spectrum (DMSO-d_6, 400 MHz) of compound **10l**.

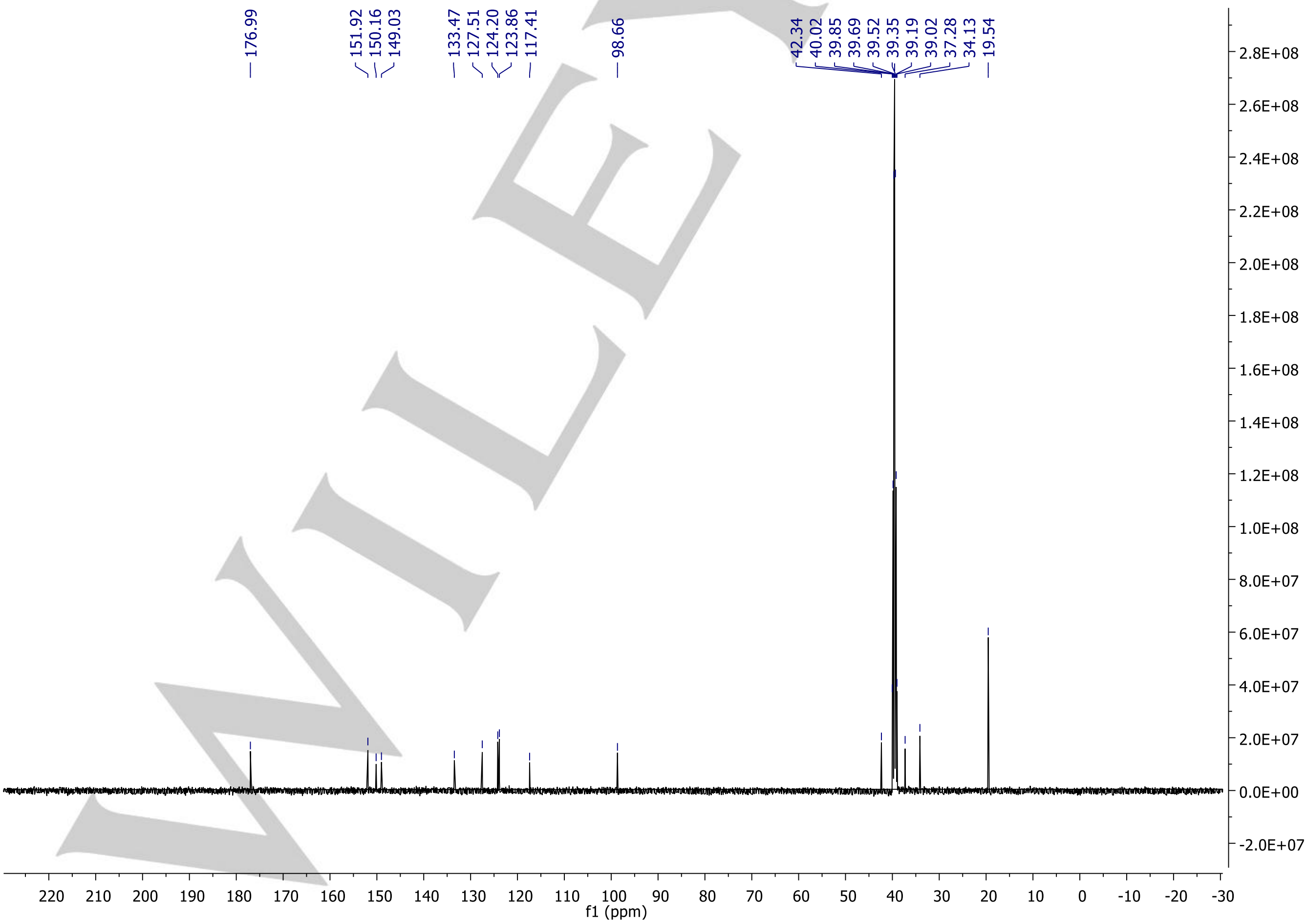

FIGURE S54 ^{13}C NMR spectrum (DMSO-d_6, 101 MHz) of compound **10l**.

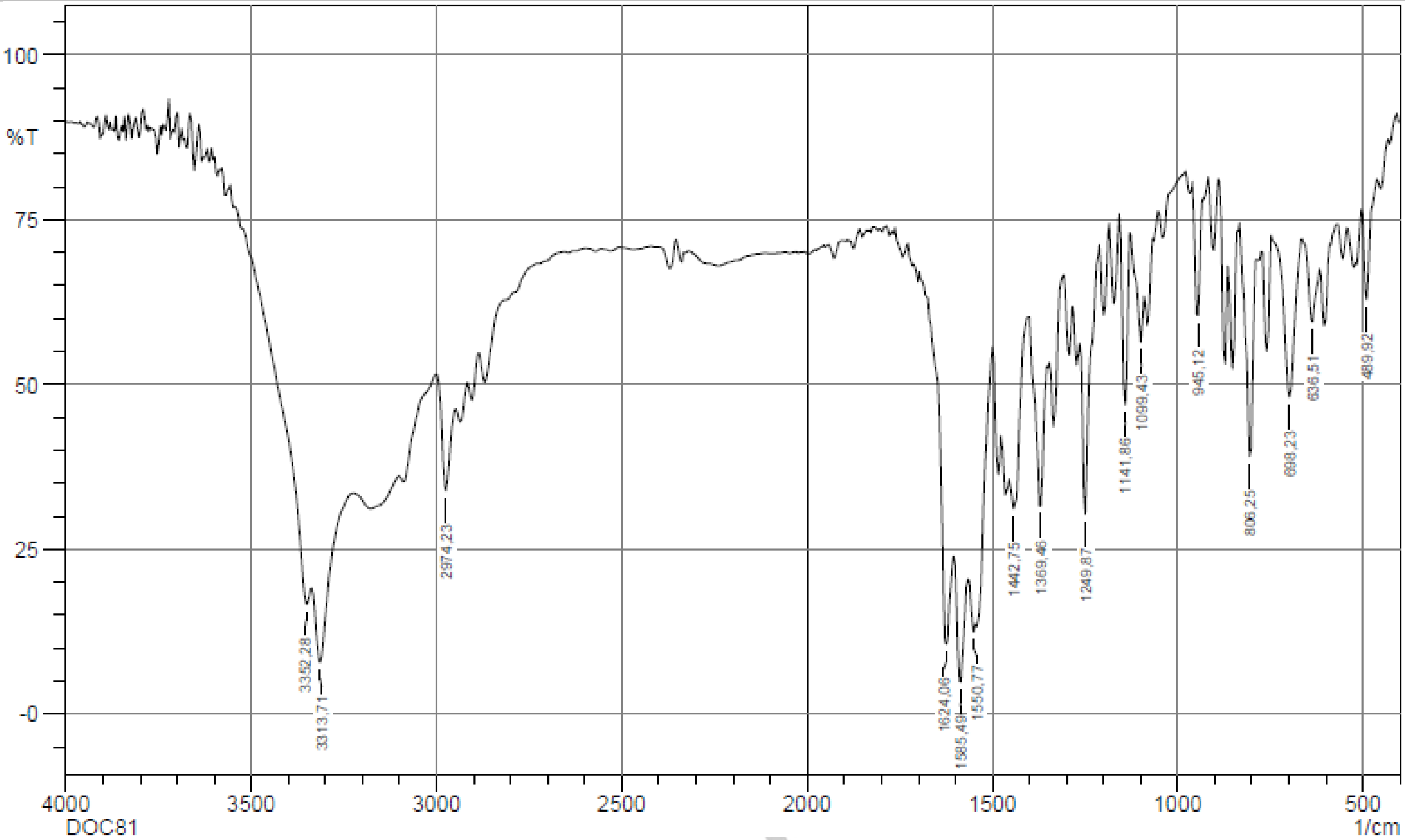

FIGURE S55 IR spectrum (KBr) of compound **10l**.

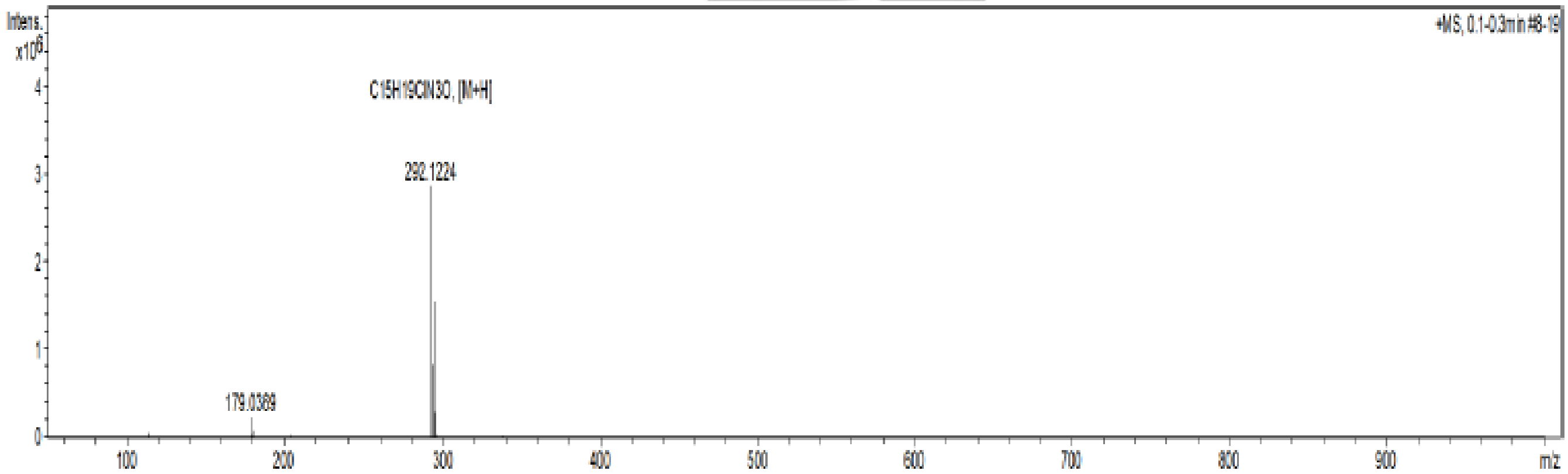

Meas. m/z	#	Ion Formula	m/z	err [ppm]	mSigma	# mSigma	Score	rdb	e⁻ Conf	N-Rule
292.1224	1	C15H19ClN3O	292.1211	-4.5	118.6	1	100.00	7.5	even	ok

FIGURE S56 HRMS spectrum (ESI) of compound **10l**.

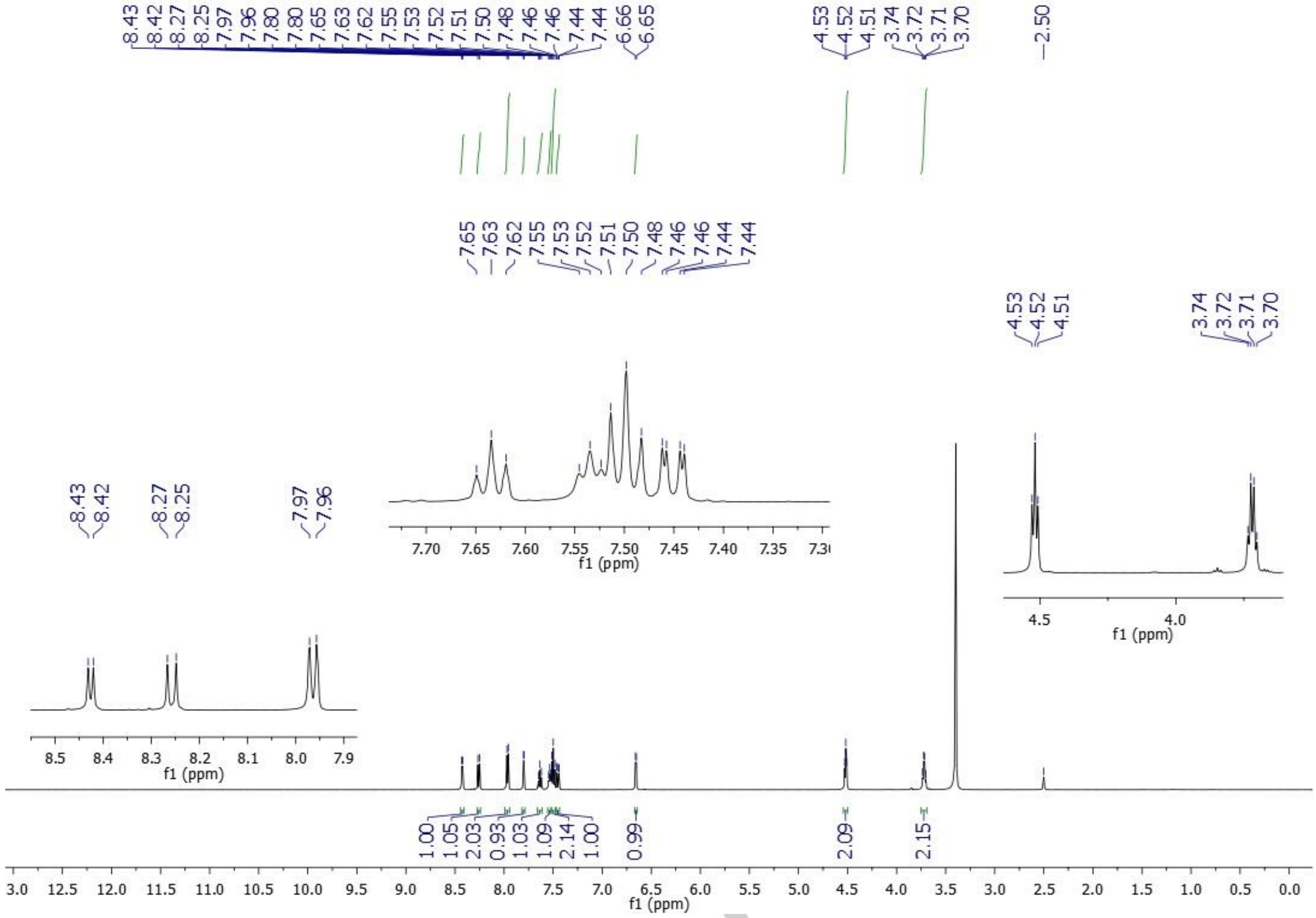

FIGURE S57 ^{1}H NMR spectrum (DMSO-d_6, 500 MHz) of compound **11**.

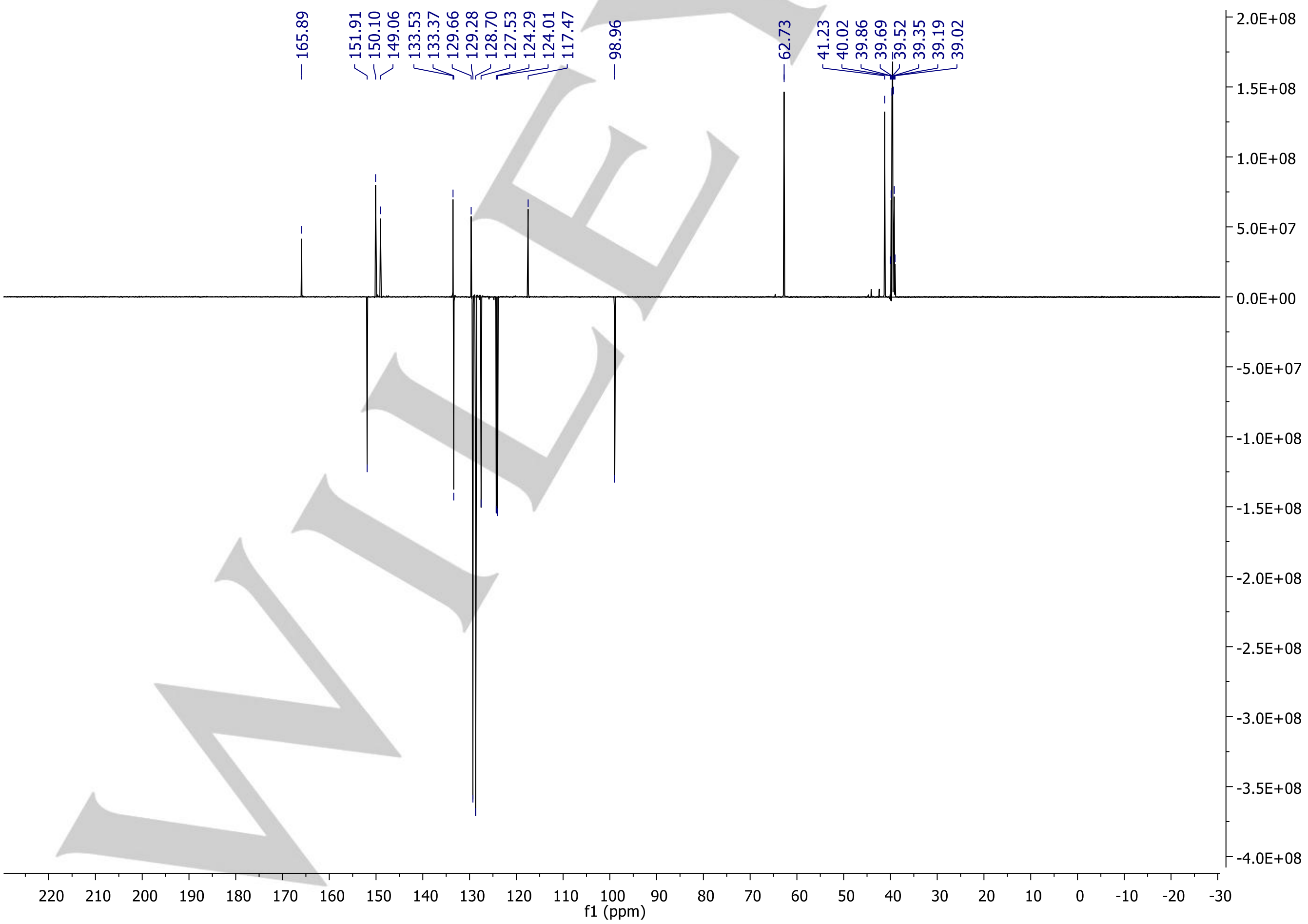

FIGURE S58 ^{13}C NMR APT spectrum (DMSO-d_6, 126 MHz) of compound **11**.

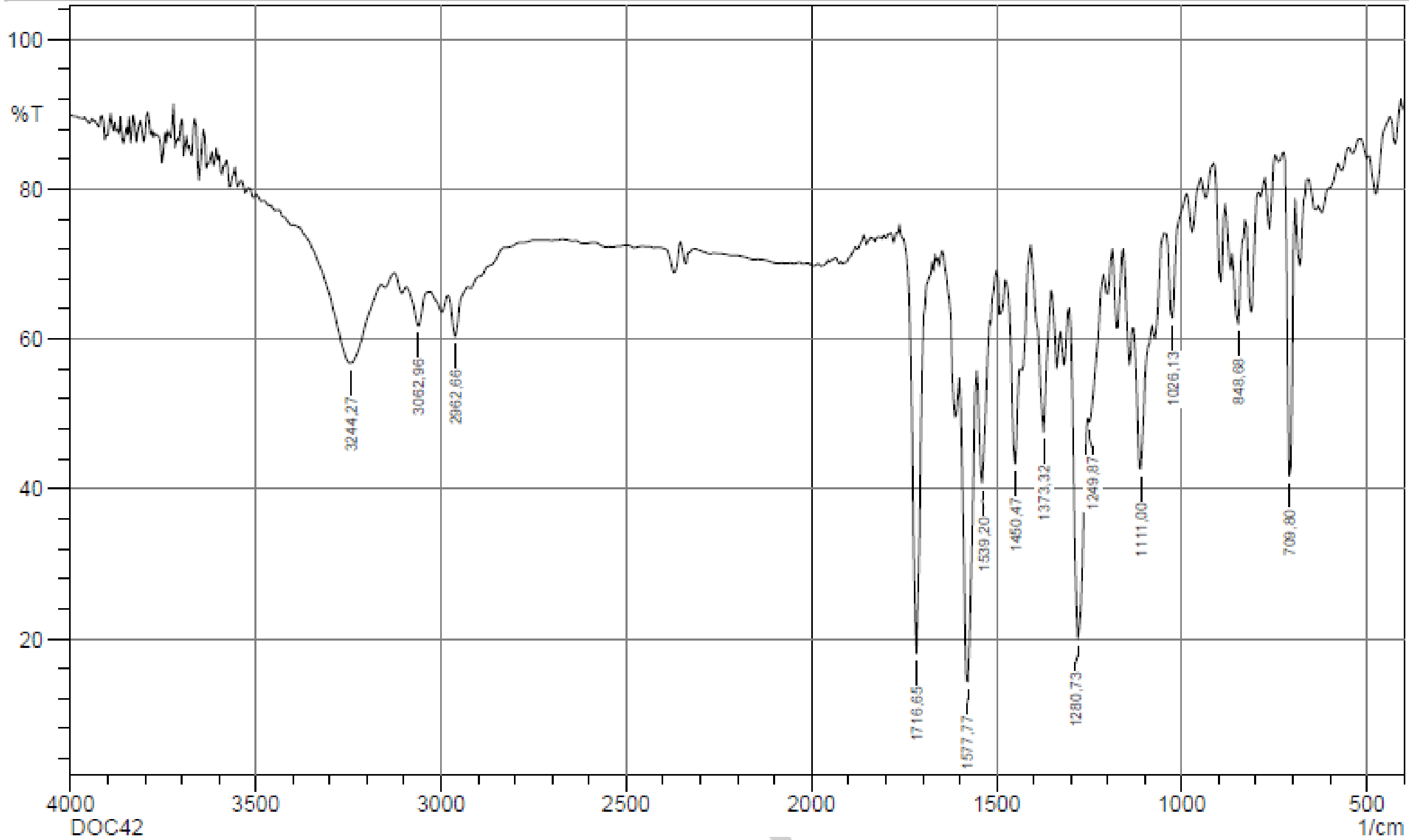

FIGURE S59 IR spectrum (KBr) of compound **11**.

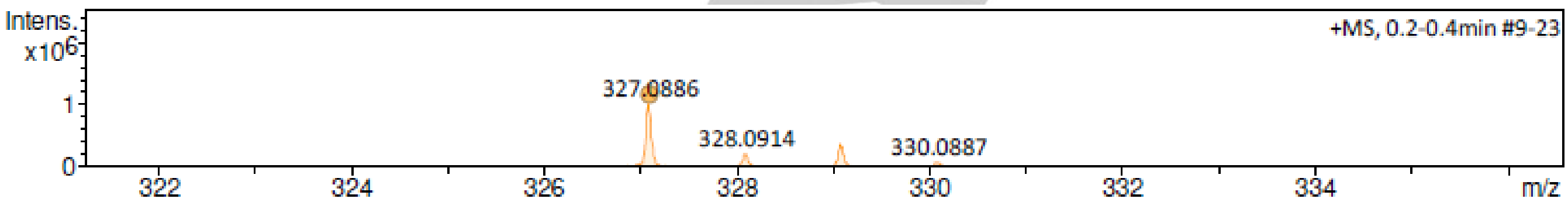

Meas. m/z	#	Ion Formula	m/z	err [ppm]	mSigma	# mSigma	Score	rdb	e⁻ Conf	N-Rule
327.0886	1	C18H16ClN2O2	327.0895	2.7	1.7	1	100.00	11.5	even	ok

FIGURE S60 HRMS spectrum (ESI) of compound **11**.

WILEY

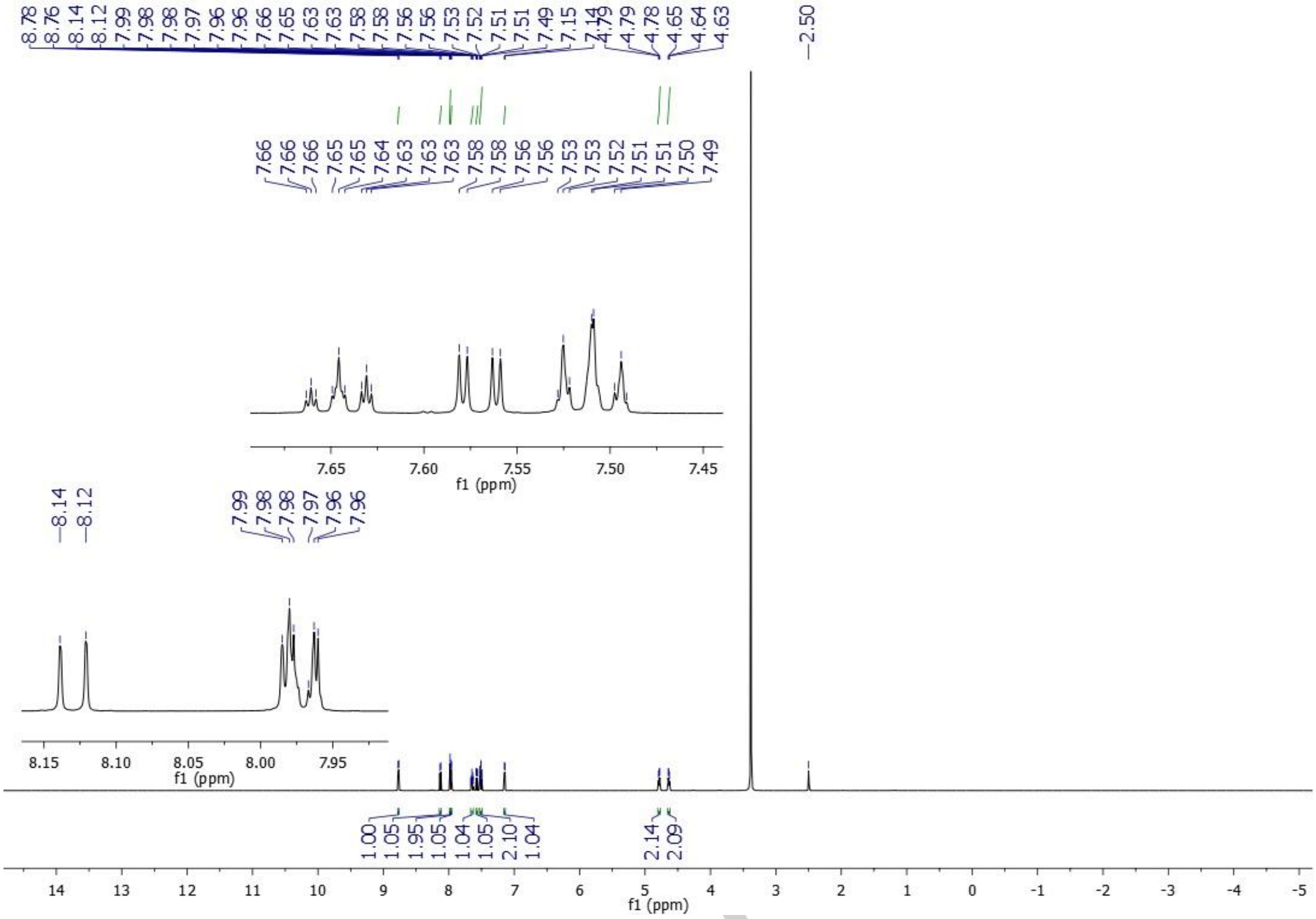

FIGURE S61 ^{1}H NMR spectrum (DMSO-d_6, 500 MHz) of compound **12**.

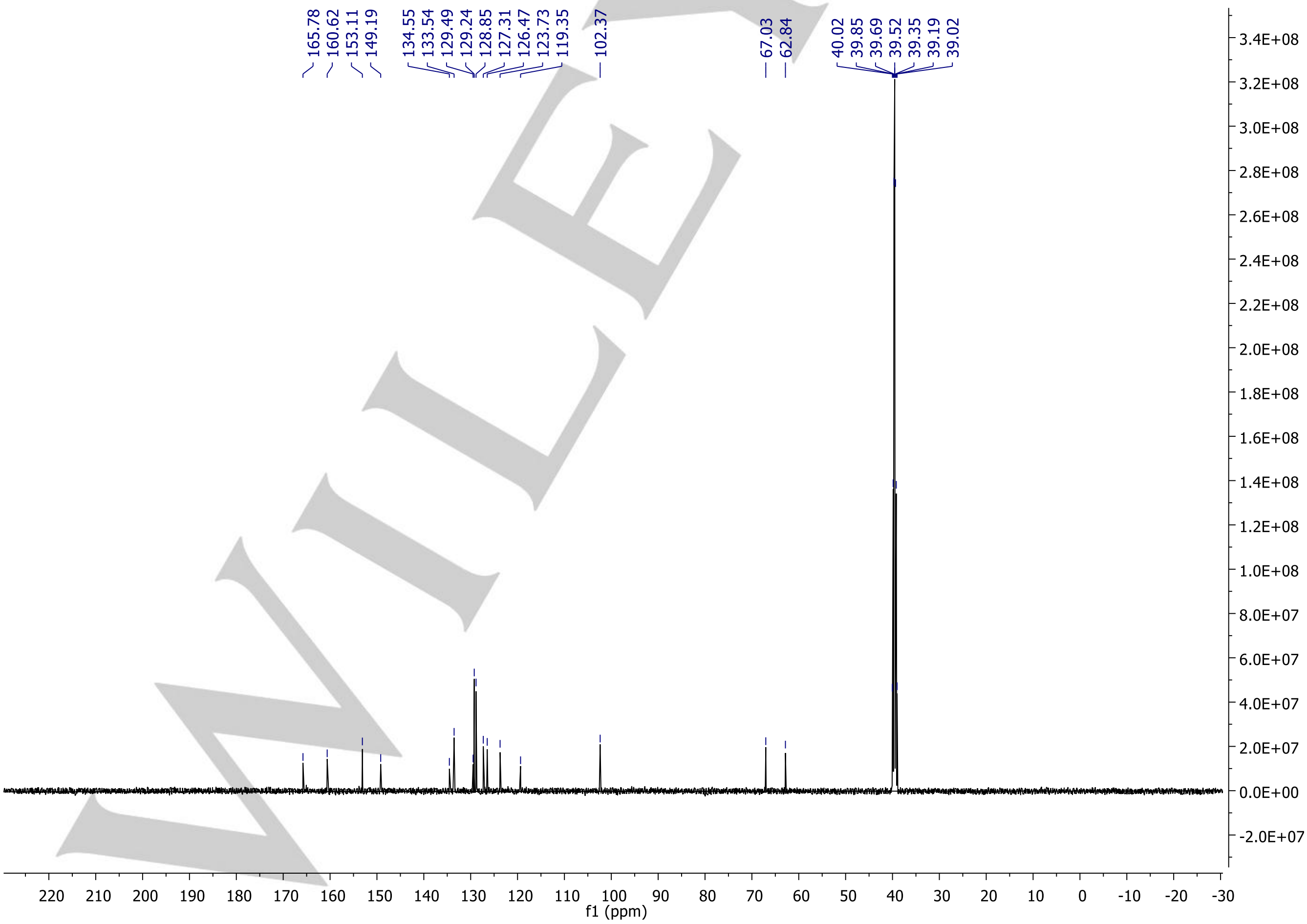

FIGURE S62 ^{13}C NMR spectrum (DMSO-d_6, 126 MHz) of compound **12**.

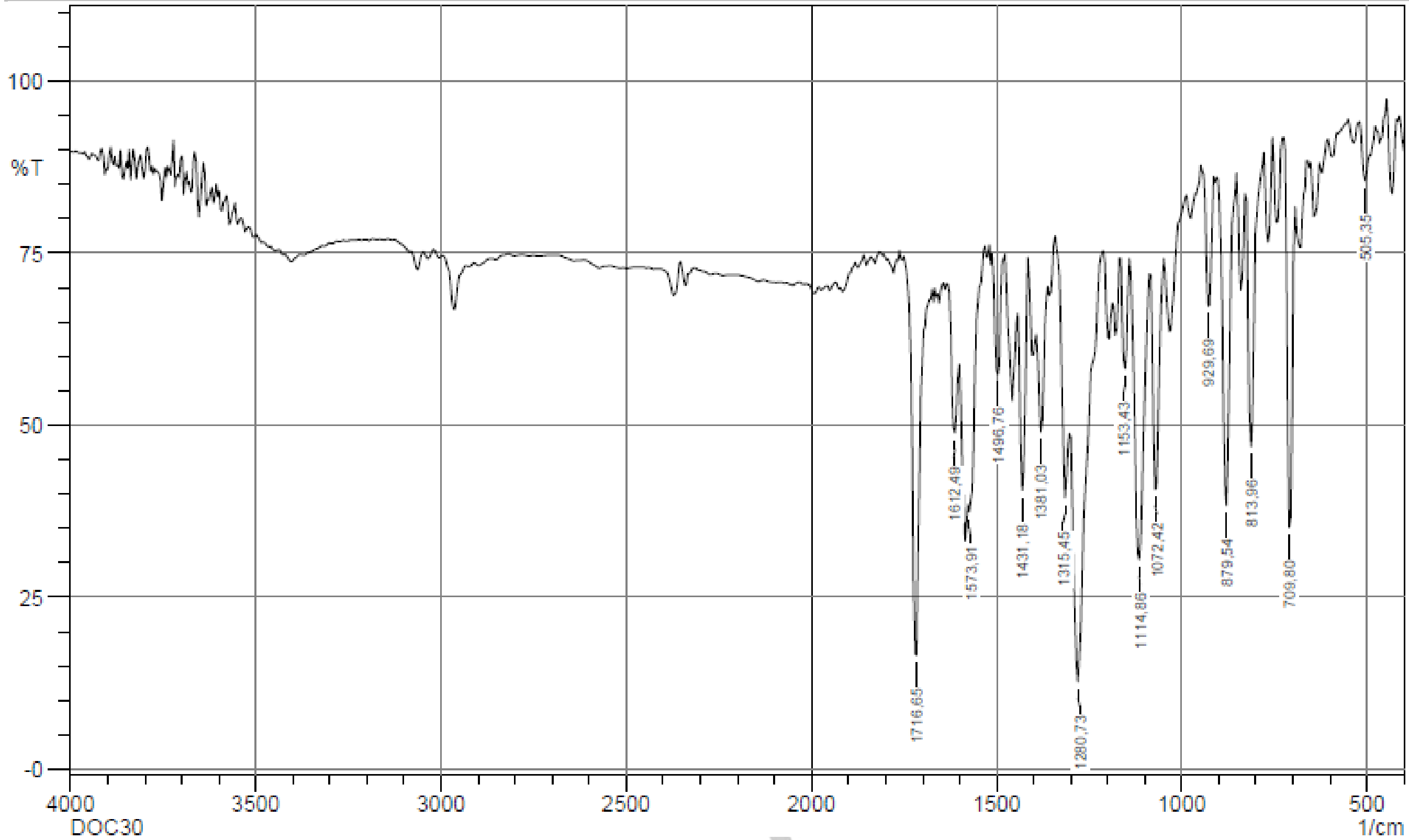

FIGURE S63 IR spectrum (KBr) of compound **12**.

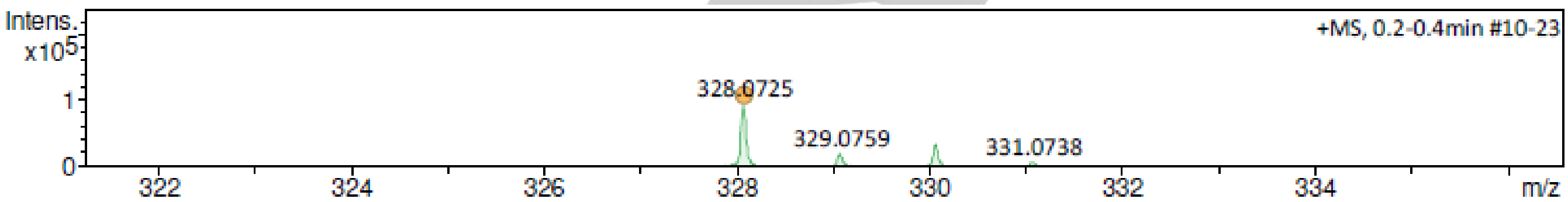

Meas. m/z	#	Ion Formula	m/z	err [ppm]	mSigma	# mSigma	Score	rdb	e⁻ Conf	N-Rule
328.0725	1	C18H15ClNO3	328.0735	3.1	3.9	1	100.00	11.5	even	ok

FIGURE S64 HRMS spectrum (ESI) of compound **12**.
